# The combination of DNA methylation and positive regulation of anthocyanin biosynthesis by MYB and bHLH transcription factors contributes to the petal blotch formation in Xibei tree peony

**DOI:** 10.1093/hr/uhad100

**Published:** 2023-05-19

**Authors:** Jin Zhu, Yizhou Wang, Qianyu Wang, Bing Li, Xiaohan Wang, Xian Zhou, Hechen Zhang, Wenzhong Xu, Shanshan Li, Liangsheng Wang

**Affiliations:** Key Laboratory of Plant Resources, Institute of Botany, Chinese Academy of Sciences, Beijing 100093, China; China National Botanical Garden Beijing 100093, China; University of Chinese Academy of Sciences, Beijing 100049, China; Key Laboratory of Plant Resources, Institute of Botany, Chinese Academy of Sciences, Beijing 100093, China; China National Botanical Garden Beijing 100093, China; University of Chinese Academy of Sciences, Beijing 100049, China; Key Laboratory of Plant Resources, Institute of Botany, Chinese Academy of Sciences, Beijing 100093, China; China National Botanical Garden Beijing 100093, China; University of Chinese Academy of Sciences, Beijing 100049, China; Key Laboratory of Plant Resources, Institute of Botany, Chinese Academy of Sciences, Beijing 100093, China; China National Botanical Garden Beijing 100093, China; University of Chinese Academy of Sciences, Beijing 100049, China; Key Laboratory of Plant Resources, Institute of Botany, Chinese Academy of Sciences, Beijing 100093, China; China National Botanical Garden Beijing 100093, China; University of Chinese Academy of Sciences, Beijing 100049, China; Key Laboratory of Plant Resources, Institute of Botany, Chinese Academy of Sciences, Beijing 100093, China; China National Botanical Garden Beijing 100093, China; University of Chinese Academy of Sciences, Beijing 100049, China; Horticulture Research Institute, Henan Academy of Agricultural Sciences, Zhengzhou 450002, China; Key Laboratory of Plant Resources, Institute of Botany, Chinese Academy of Sciences, Beijing 100093, China; China National Botanical Garden Beijing 100093, China; University of Chinese Academy of Sciences, Beijing 100049, China; Key Laboratory of Plant Resources, Institute of Botany, Chinese Academy of Sciences, Beijing 100093, China; China National Botanical Garden Beijing 100093, China; University of Chinese Academy of Sciences, Beijing 100049, China; Key Laboratory of Plant Resources, Institute of Botany, Chinese Academy of Sciences, Beijing 100093, China; China National Botanical Garden Beijing 100093, China; University of Chinese Academy of Sciences, Beijing 100049, China

## Abstract

Xibei tree peony is a distinctive cultivar group that features red–purple blotches in petals. Interestingly, the pigmentations of blotches and non-blotches are largely independent of one another. The underlying molecular mechanism had attracted lots of attention from investigators, but was still uncertain. Our present work demonstrates the factors that are closely related to blotch formation in *Paeonia rockii* ‘Shu Sheng Peng Mo’. Non-blotch pigmentation is prevented by the silencing of anthocyanin structural genes, among which *PrF3H*, *PrDFR*, and *PrANS* are the three major genes. We characterized two R2R3-MYBs as the key transcription factors that control the early and late anthocyanin biosynthetic pathways. PrMYBa1, which belongs to MYB subgroup 7 (SG7) was found to activate the early biosynthetic gene (EBG) *PrF3H* by interacting with SG5 member PrMYBa2 to form an ‘MM’ complex. The SG6 member PrMYBa3 interacts with two SG5 (IIIf) bHLHs to synergistically activate the late biosynthetic genes (LBGs) *PrDFR* and *PrANS*, which is essential for anthocyanin accumulation in petal blotches. The comparison of methylation levels of the *PrANS* and *PrF3H* promoters between blotch and non-blotch indicated a correlation between hypermethylation and gene silencing. The methylation dynamics of *PrANS* promoter during flower development revealed a potential early demethylating reaction, which may have contributed to the particular expression of *PrANS* solely in the blotch area. We suggest that the formation of petal blotch may be highly associated with the cooperation of transcriptional activation and DNA methylation of structural gene promoters.

## Introduction

Red–purple petal blotch is a typical trait of Xibei tree peony, which was inherited from the species *Paeonia rockii* and is widespread across various cultivar groups [1, 2]. The blotch trait gives flowers rich variations in colors and effectively enhances the ornamental value. Tree peony has long served as the representative flower of China and has been praised as ‘king of flowers’. As its popularity continues to soar, the king flower now attracts worldwide attention. Therefore, breeding for flower color, particularly the innovation of dual color on which the Xibei tree peony depends, is highly promising. However, the mystery of blotch formation has baffled horticultural breeders and researchers for a long time. It is very challenging to do pertinent research in the absence of tree peony genetic bases and transgenic systems, and thus the mechanism of blotch formation remains unsolved.

In actuality, petals with spotted or striped pigmentation patterns are common in plants [3–6]. It is presumed that flower color patterns provide important signals to advertise rewards for the pollinators, and have even evolved to match specific preferences of pollinators based on their visual systems [7–9]. For instance, monkeyflower (*Mimulus*) species with pink or red flowers attract bumblebees or hummingbirds as pollinators, respectively [10].

Flavonoids, especially anthocyanins, are the major pigments that confer a wide range of colors on flowers or other reproductive organs of angiosperms [11]. Dicotyledon flavonoid biosynthesis is mainly controlled by two separate subsets of structural genes that have been divided into two groups: early biosynthetic genes (EBGs, including *CHS*, *CHI*, *F3H*, *F3′H*, and *F3′5′H*) and late biosynthetic genes (LBGs, including *DFR*, *ANS*, and *UF3GT*) [12]. Extensive research has been done on the functions of these structural genes and almost all of them have previously been cloned and characterized for maize, snapdragon, *Petunia*, *Arabidopsis*, and many other angiosperms [13]. It has been discovered that the EBGs produce precursors for the whole of the flavonoid pathways, while the LBGs encode enzymes specific to the anthocyanin and proanthocyanidin pathways.

The gorgeous flowers of tree peony are mostly colored by anthocyanin products, which are composed of cyanidin (Cy), peonidin (Pn), and pelargonidin (Pg) derivatives [14–16]. Only a small number of structural genes, including *PsCHS-1*, *PsAOMT*, and *PsF3′H*, have been characterized in tree peony according to previous reports [17–19]. In recent years, a large number of transcriptome investigations have been conducted, which has laid a good foundation for research on flavonoid biosynthesis in tree peony [20–23]. Nonetheless, there are significant variations among tree peony cultivars, and pertinent studies are typically unfocused. Current studies are still unable to offer reliable foundations for thorough and systematic research on the molecular basis of flower pigmentation. Therefore, a proper investigation of structural gene functions is required for elucidating the variation of flower color in tree peony.

The molecular basis of color patterning has recently been determined in several plant species. In most cases it depends primarily on the regulation of flavonoid (anthocyanin) biosynthesis by transcription factors. Certain R2R3-MYB and bHLH family members, as well as some WD-repeat (WDR) proteins, specifically activate the anthocyanin biosynthetic genes via MBW core activation complex or through alternative mechanisms with spatiotemporal specificity [5, 6, 24–28]. Among the three types of transcription factor, MYB is crucial for controlling flavonoid metabolism. The R2R3-MYB transcription factors related to flavonoid biosynthesis were assumed to have conservative functions among subgroups, e.g. subgroup 6 (SG6) for anthocyanin biosynthesis, SG7 for flavonol, and SG5 for proanthocyanidin biosynthesis [29]. Although the R2R3-MYB transcription factors have been intensively studied and have even been used in plant molecular breeding [30, 31], little is known about how they regulate anthocyanin biosynthesis in tree peony, particularly the Xibei tree peony. We summarize in Table 1 all transcription factor genes previously identified by researchers that may be involved in flavonoid biosynthesis in tree peony. They all reportedly act on one or several target genes to activate the flavonoid biosynthetic pathway. PsMYB12 was reported to interact with bHLH and WD40 to activate *PsCHS* expression, which contributes to the anthocyanin accumulation in petals of *Paeonia suffruticosa* ‘Qing Hai Hu Yin Bo’ [32]. A recent study reported that PsMYB30 may activate *PsANS* expression to promote blotch pigmentation in *P. suffruticosa* ‘High Noon’ [33]. Except for *PsMYB12* and *PsMYB30*, it has not been entirely proven how the remaining genes function, according to the reports.

**Table 1:** Identified transcription factors genes associated with flavonoid biosynthesis in *P. suffruticosa*.

***Cultivar***	**Gene**	**Proven/potential target genes**	**Reference**
‘Qing Hai Hu Yin Bo’	*PsMYB12*	*PsCHS*	[32]
‘ShimaNishiki’	*PsMYB114L*	*PsFLS*, *PsANR*	[46]
	*PsMYB12L*	*PsDFR*, *PsANS*, *PsF3′H*	
‘Er Qiao’	*PsMYB57*		[47]
	*PsMYB58*		[48]
‘High Noon’	*PsMYB111*	*PsFLS*	[22]
	*PsMYB30*	*PsANS*	[33]
‘Hei Hua Kui’	*PsbHLH1*	*PsDFR*, *PsANS*	[49]
‘Er Qiao’	*PsbHLH1*		[48]
	*PsbHLH3*		

In addition to the transcriptional regulation, DNA methylation has been revealed to be a general mechanism controlling gene expression to regulate plant secondary metabolism [34–36]. It has been discovered in horticultural plants that DNA methylation affects the activity of the anthocyanin biosynthetic pathways. For instance, MdMYB10 is a transcription factor that upregulates the anthocyanin production of apple, and the methylation of *MdMYB10* promoter results in variable color patterns of apple peel [37]. The hypermethylation of the *PcMYB10* promoter is closely related to the formation of green-skinned sport in the ‘Max Red Bartlett’pear [38]. In addition, the regulation of anthocyanin biosynthesis by DNA methylation has also been reported in radish, peach, orange, and many other species [39–41]. DNA methylation was also considered to be a regulatory mechanism which inhibits the flower pigmentation in ornamental plants, relevant studies have been carried out in *Oncidium* ‘Gower Ramsey’, lotus, peach and chrysanthemum [42–45].

In this study, the potential mechanisms of petal blotch formation in the Xibei tree peony were discussed. The EBGs and LBGs of anthocyanin biosynthesis were activated by R2R3-MYB and bHLH transcriptional regulators in certain modes, which provides the precondition for blotch pigmentation. We presents the new idea that the absence of anthocyanin accumulation in non-blotched area may be attributed to the inactivation of structural genes, which is primarily caused by the hypermethylation of their promoters.

## Results

### The flavonoid compounds in petals of *Paeonia rockii* ‘Shu Sheng Peng Mo’

**Figure 1:**
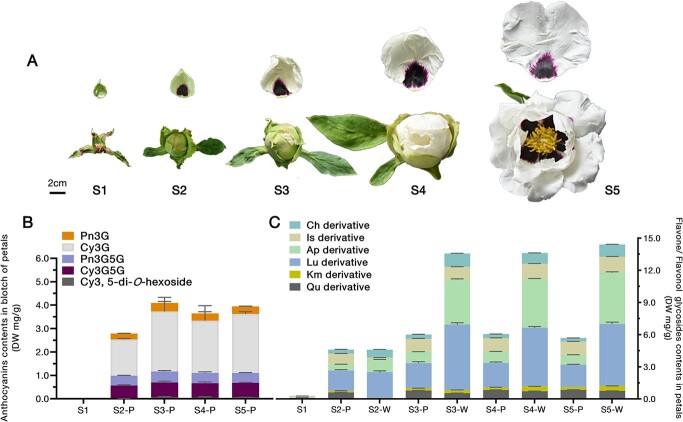
Flavonoid composition in petals during flower development. (**A**) Five developmental stages of *P. rockii* ‘Shu Sheng Peng Mo’ flower and petal. S1, ~25 days before bloom, petal without blotch; S2, ~15 days before bloom, dark purple blotch has appeared; S3, ~7 days before bloom, blotch has grown and is still dark; S4, ~2 days before bloom, blotch has grown in size and is still dark; S5, bloom time, blotch is bright and showy, but no change in size. Scale bars = 2 cm. (**B**) Anthocyanin contents in blotch area during the S1–S5 stages. (**C**) Flavone and flavonol glycoside contents in two areas of petal during the S1–S5 stages. Pn, peonidin; Cy, cyanidin; Ch, chrysoeriol; Is, isorhamnetin; Ap, apigenin; Lu, luteolin; Km, kaempferol; Qu, quercetin. P or W represents blotch or non-blotch area of petal. DW, dry weight.

Five explicit stages were determined during flower development (Fig. 1A); we investigated the flavonoid compositions in two different areas (blotch and non-blotch) of the petals, which were designated as stage 1 (S1) to stage 5 (S5). Anthocyanins were only found in blotches, and four definite anthocyanins were identified: peonidin 3-*O*-glucoside (Pn3G), cyanidin 3-*O*-glucoside (Cy3G), peonidin 3,5-di-*O*-glucoside (Pn3G5G), and cyanidin 3,5-di-*O*-glucoside (Cy3G5G). A Cy-type double-glycoside that was indeterminate was identified as cyanidin-3,5-di-*O*-hexoside (Fig. 1B). Flavones and flavonols were found in both blotch and non-blotch areas, including five flavone glycosides and six flavonol glycosides (Fig. 1C; Supplementary Data Table S1). Results showed that the predominant anthocyanin compositions in blotches were cyanidin glycosides (relative content = 91.85%, S5), and the content of flavone glycosides in the non-blotch area was significantly higher than in blotches, of which apigenin and luteolin glycoside were the main components (Supplementary Data Table S2; Fig. 1). The anthocyanin abundance began to increase sharply at S2, then remained steady or even decreased slightly. In the non-blotch area, flavone and flavonol glycosides showed significant accumulation at S2, followed by a rapid increase from S2 to S3. The HPLC-DAD (HPLC with diode array detection) chromatogram is displayed in Supplementary Data Fig. S1.

### Anthocyanin biosynthetic genes were specifically expressed in blotches

**Figure 2:**
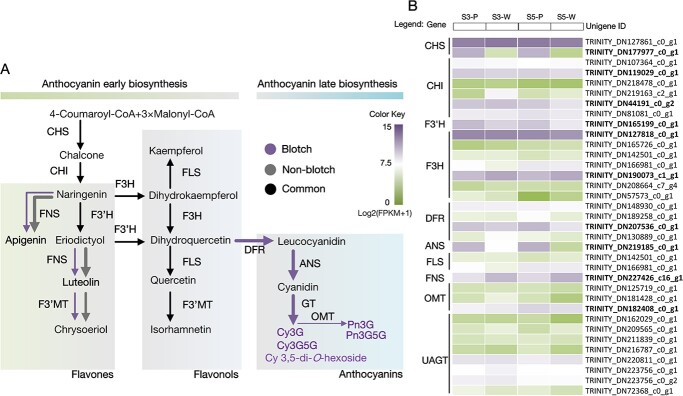
Putative metabolic flux of the flavonoid biosynthetic pathway and transcriptome profiles of structural genes in blotch and non-blotch areas. (**A**) Flavonoid biosynthetic pathway in blotch and non-blotch areas. Black arrows represent the common direction of metabolic flow and the purple and gray arrows represent blotch and non-blotch areas, respectively. The thickness of purple and gray arrows represents the relative size of metabolic flux, which was set based on the content of the end products we quantified. (**B**) Transcriptome profiles of structural genes in blotched (P) and non-blotched (W) areas. Data are based on FPKM values; S3 and S5 represent petals of stage 3 and stage 5. IDs of potential DEGs are marked in bold.

According to the relative content of the end flavonoid products, the flavonoid biosynthetic pathways in blotch and non-blotch areas of petal were presumed to show differences in metabolic flux (Fig. 2A). To investigate why anthocyanins accumulated only in blotches, we performed RNA sequencing (RNA-seq) of the blotch and non-blotch areas (petals of S1, S3, and S5 as samples) to identify the candidate genes for petal pigmentation. Data analyses were focused on the unigenes putatively involved in flavonoid biosynthetic pathways, especially in the anthocyanin branch. Based on the functional annotations, all the unigenes encoding enzymes had been identified, including *CHS*, *CHI*, *F3′H*, *F3H*, *FNS*, *FLS*, *DFR*, *ANS*, *OMT*, and *UAGT*. Potential differentially expressed genes (DEGs) in the blotch and non-blotch areas were selected based on the fragments per kilobase million (FPKM) value, including *CHS* (TRINITY_DN177977_c0_g1), *CHI* (TRINITY_DN119029_c0_g1, TRINITY_DN44191_c0_g2), *FNSII* (TRINITY_DN227426_c16_g1), *F3′H* (TRINITY_DN165199_c0_g1), *F3H* (TRINITY_DN127818_c0_g1, TRINITY_DN190073_c1_g1), *DFR* (TRINITY_DN207536_c0_g1), *ANS* (TRINITY_DN219185_c0_g1), and *OMT* (TRINITY_DN182408_c0_g1) (Fig. 2B). To validate the RNA-seq data, the spatiotemporal relative expression of these unigenes was analyzed using quantitative real-time PCR (qRT–PCR). The four unigenes *CHS* (TRINITY_DN177977_c0_g1), *F3H* (TRINITY_DN127818_c0_g1), *DFR* (TRINITY_DN207536_c0_g1), and *ANS* (TRINITY_DN219185_c0_g1) showed blotch-specific high expression, while unigenes *F3H-2* (TRINITY_DN190073_c1_g1) and *FNSII* (TRINITY_DN227426_c16_g1) were the opposite (Supplementary Data Fig. S2). The results of qRT–PCR were consistent with the RNA-seq data.

### Identification of MYB transcription factors involved in anthocyanin biosynthesis

A total of 53 full-length R2R3-MYB genes were identified in petals based on information on functional annotations and the *de novo* assembly. In order to screen the MYB regulators responsible for the regulation of flavonoid biosynthesis, a phylogenetic tree was constructed with the neighbor-joining method, using the 53 proteins from *P. rockii* ‘Shu Sheng Peng Mo’ and 87 proteins from *Arabidopsis* (Supplementary Data Fig. S3). The majority of the tree peony R2R3-MYBs were clustered into 23 subgroups with the MYBs from *Arabidopsis*, of which 14 members belonged to the subgroups SG4, SG5, SG6, and SG7, which are reported to be involved in the flavonoid biosynthesis [29, 31]. As a previous study has shown that PsMYB12 directly activates *PsCHS* expression but represents a distinct subgroup [32], the two PrMYBs that clustered with PsMYB12 and the PsMYB12 (a highly homologous protein in *P. rockii* ‘Shu Sheng Peng Mo’) were also taken into consideration. Accordingly, a total of 17 genes that encode R2R3-MYBs were used for subsequent research. We eventually retained 12 genes and removed the four genes that had low abundance at S1, S3, and S5 based on the transcriptome profiles (Supplementary Data Fig. S4A). The full-length cDNA sequences of the 12 genes were cloned from the blotch and non-blotch areas. It was discovered that there is no variation in the sequences of the 12 genes between the two areas, and all of the sequences roughly agree with the assembly data (Supplementary Data Fig. S5). The 12 PrMYB proteins ranged from 193 (TRINITY_DN184394_c0_g1) to 379 (TRINITY_DN22164_c0_g2) amino acids in length and contained the highly conserved R2R3 DNA-binding domain at the N-terminal and the bHLH-interacting motif ([D/E] L_x2_[R/K]_x3_L_x6_L_x3_R) in the R3 region [29, 50]. The C-terminal of the 12 protein sequences varied greatly; five of them (TRINITY_DN80390_c0_g2, TRINITY_DN45879_c0_g1, TRINITY_DN22762_c0_g1, TRINITY_DN186385_c0_g1, and TRINITY_DN210991_c0_g1) contained the two conserved motifs, C1 motif (LIsrGIDPxT/SHRxI/L) and C2 motif (pdLNLD/ELxiG/S), which were identified as the characteristics of R2R3-MYB repressors (Supplementary Data Fig. S4B) [31]. In subsequent investigations, we focused on the transcriptional regulation of the structural genes *PrF3H*, *PrDFR*, and *PrANS* to explore the reasons behind their differential expressions in blotch and non-blotch areas.

### PrMYBa1 directly binds to the promoter of EBG *PrF3H* and activates its transcription

**Figure 3:**
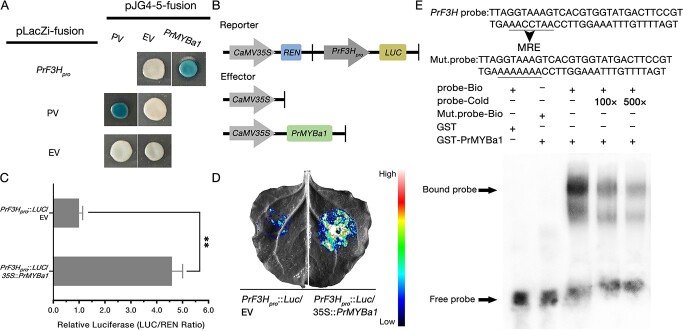
PrMYBa1 binds to the *PrF3H* promoter and activates its transcription. (**A**) Result of Y1H assay showing that PrMYBa1 binds to the *PrF3H* promoter. EV, empty vector; PV, positive vector. pLacZi-*AtLDOX_pro_* (−1702 to −1 bp) and pJG4–5-*AtPAP1* were used as positive control. (**B**) Schematic representation of the reporter and effectors. (**C**) Relative luciferase activities (LUC/REN ratio) for co-expressed *PrF3H_pro_*::*LUC + 35S*::*PrMYBa1* and *PrF3H_pro_*::*LUC +* EV. Values represent mean ± standard deviation (*n* = 4). The value of the negative control was used as the reference and set to 1, error bars denote standard deviations, and asterisks indicate a statistically significant difference (two-sided Student’s *t*-test; ^**^P < .01). (**D**) Representative image of an *N. benthamiana* leaf 3 days after co-infiltration. (**E**) EMSA showing the binding affinity of PrMYBa1 to the promoter of *PrF3H*. Mult. Probe represents the mutated probe, in which the bases of MBS or MRE elements were all replaced by A. ‘+’ and ‘−’ indicate the presence and absence of the corresponding probe or protein, respectively. All the experiments were performed more than three times independently and similar results were obtained.

We used the genome walking method to clone the promoter sequence of *PrF3H* (TRINITY_DN127818_c0_g1) in order to identify the transcription factors that are working on this blotch-specific high-transcription EBG. The putative *cis*-acting regulatory elements were identified using the PlantCARE database (http://bioinformatics.psb.ugent.be/webtools/plantcare/html/). An MYB-binding site (MRE) and a CCAAT-box (MYB recognition site) [51] were discovered in the region from −509 to −496 bp (Supplementary Data Fig. S6). In order to further ascertain if *PrF3H* is regulated by one or several R2R3-MYBs of the 12 PrMYBs mentioned above, the complete fragment of the *PrF3H* promoter was cloned into a pLacZi plasmid to generate bait constructs for a yeast one-hybrid (Y1H) assay. The outcome revealed that six PrMYB proteins were bound to the promoter fragment (Supplementary Data Fig. S7A). We then validated whether they operate as transcriptional activators or not using a dual-luciferase (dual-LUC) reporter assay in *Nicotiana benthamiana* leaves. A fragment of the *PrF3H* promoter was fused to the *LUC* gene to generate a reporter vector, and the overexpressing vector pSN1301 carrying one of the *PrMYB*s was used as effector; the empty vector was used as the negative control. When the reporter construct was co-transfected with the effector plasmid, we found that one of the six PrMYBs activated the reporter gene via acting on the promoter of *PrF3H* (Supplementary Data Fig. S7B). The activator was then named PrMYBa1 (TRINITY_DN22164_c0_g2). By using effective Y1H and dual-LUC reporter assays, PrMYBa1 was found to act on the promoter of *PrF3H* (Fig. 3A–D). Then, an electrophoretic mobility shift assay (EMSA) was used to test the binding affinity of PrMYBa1 and the *PrF3H* promoter. The recombinant protein PrMYBa1-GST was expressed in *Escherichia coli* cells and purified, and the promoter fragment that contained the MRE element was used as a probe. A band shift was observed when the PrMYBa1-GST protein was mixed with biotin-labeled probe, while the intensity of the shifted bands was reduced by increasing concentrations of cold competitor probe (non-biotin-labeled) (Fig. 3E). This evidence confirms that PrMYBa1 directly binds to the *PrF3H* promoter to activate its transcription.

### PrMYBa2 interacts with PrMYBa1 to form an ‘MM’ complex and enhances the activation of *PrF3H*

**Figure 4:**
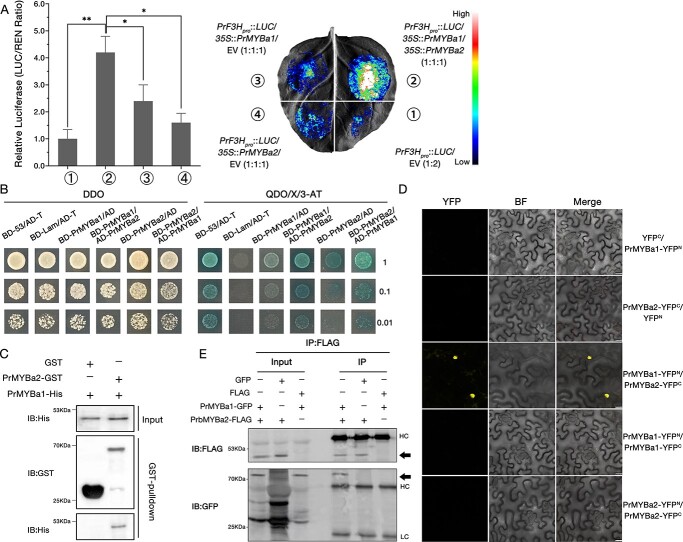
PrMYBa2 interacts with PrMYBa1 and enhances the activation of *PrF3H*. (**A**) Dual-LUC reporter assay showing that activation of the reporter gene was enhanced with the addition of PrMYBa2. Relative luciferase activities for co-expressed *PrF3H_pro_*::*LUC + 35S*::*PrMYBa1*, *PrF3H_pro_*::*LUC + 35S*::*PrMYBa2*, *PrF3H_pro_*::*LUC + 35S*::*PrMYBa1 + 35S*::*PrMYBa2*, and *PrF3H_pro_*::*LUC +* EV (left). Image of an *N. benthamiana* leaf 3 days after co-infiltration (right). All components are in equal proportions (1:1:1). Values represent means ± standard deviation (*n* = 4) and asterisks indicate statistically significant differences (two-sided Student’s *t*-test; ^*^P < .05, ^**^P < .01). (**B**) Y2H assay showing the interaction between PrMYBa1 and PrMYBa2 proteins. The positive control was pGBKT7–53 + pGADT7-T while the negative control was pGBKT7-lam + pGADT7-T. AD, pGADT7 vector; BD, pGBKT7 vector; DDO, SD medium without Trp and Leu; QDO/X/3-AT, SD medium without Trp, Leu, His, Ade, supplemented with X-*α*-gal and the right amount of 3-AT. Transformed yeast cells were dotted at 10^−1^ dilution on the selective media. (**C**) Pull-down assay of PrMYBa1 and PrMYBa2. PrMYBa1-His protein was incubated with PrMYBa2-GST and GST proteins, respectively. Proteins pulled down by GST resin beads were then detected using anti-GST and anti-His antibodies. Predicted molecular mass of proteins: GST, 26 kDa; PrMYBa1-His, 45 kDa; PrMYBa2-GST, 64 kDa. (**D**) BiFC showing the interaction between PrMYB1 and PrMYB2 in *N. benthamiana* leaves. PrMYB1 and PrMYB2 were fused with either the C- or N-terminus of yellow fluorescent protein (named YFP^C^ or YFP^N^), respectively. YFP, YFP fluorescence; BF, white light; Merge, YFP and white light combined signal. Scale bars = 25 μm. (**E**) Co-IP assay of PrMYBa1 and PrMYBa2 in *N. benthamiana* leaves. Arrows indicate FLAG and GFP fusion proteins detected in the immunoprecipitation (IP) group. Predicted molecular mass of proteins: GPF, 27 kDa; FLAG, 3 kDa; PrMYBa1-GFP, 75 kDa; PrMYBa2-FLAG, 35 kDa. Total proteins were extracted 3 days after infiltration. Anti-GFP and anti-FLAG antibodies were used for immunoprecipitation. IB, immunoblotting; HC, heavy chain; LC, light chain.

To avoid the impact of potential false-negative results in Y1H analysis, a dual-LUC reporter assay was employed as a complementary validation of the functions of all the 12 PrMYBs. Accidentally, we discovered a second PrMYB, designated as PrMYBa2 (TRINITY_DN210213_c0_g1), which marginally affected the *PrF3H* promoter in repeated dual-LUC reporter assays (Supplementary Data Fig. S8). What is notable is that the Y1H analytical result for PrMYBa2 was negative (Supplementary Data Fig. S7A). In order to ascertain the role of PrMYBa2, we used a multi-component dual-LUC reporter assay to investigate its function. *Agrobacterium tumefaciens* was mixed 1:1 in volume with the vectors pSN1301-PrMYBa1 and pSN1301-PrMYBa2 to serve as effectors simultaneously. An extremely strong signal was detected on the *N. benthamiana* leaf co-transfected with the double effectors, which were obviously different from that transfected with single effector (Fig. 4A). Therefore, we propose that PrMYBa2 improved the activation of reporter gene *LUC*. To further investigate the synergy of the two PrMYBs, a yeast two-hybrid (Y2H) assay was used to evaluate the interaction between PrMYBa1 and PrMYBa2 proteins. As expected, PrMYBa2 was found to interact with PrMYBa1 in yeast cells (Fig. 4B). In order to further support this conclusion, PrMYBa1-His and PrMYBa2-GST fusion proteins were expressed in *E. coli* cells and purified for a pull-down assay. According to the result of western blotting, PrMYBa1 was pulled down by PrMYBa2 (Fig. 4C). In addition, the bimolecular fluorescence complementation (BiFC) assay revealed that the yellow fluorescent protein (YFP) fluorescence signals were captured when PrMYBa1-YFP^N^ and PrMYBa2-YFP^C^ were co-expressed in cells. The result of the BiFC assay suggested that PrMYBa2 interacted with PrMYBa1 in the cell nuclei of *N. benthamiana* leaves (Fig. 4D). To further confirm the interaction of PrMYBa1 and PrMYBa2 *in vivo*, we performed co-immunoprecipitation (Co-IP) assays of the two PrMYBs. PrMYBa1-GFP and PrMYBa2-FLAG were co-expressed in *N. benthamiana* leaves; the proteins were precipitated with anti-FLAG antibody and then probed with anti-GFP antibody. The result showed that PrMYBa1-GFP protein was only detected in samples that co-expressed PrMYBa1-GFP and PrMYBa2-FLAG, confirming that PrMYBa1 interacts with PrMYBa2 *in planta* (Fig. 4E).

### PrMYBa3 binds to the promoters of LBGs *PrDFR* and *PrANS* and directly activates their transcription

**Figure 5:**
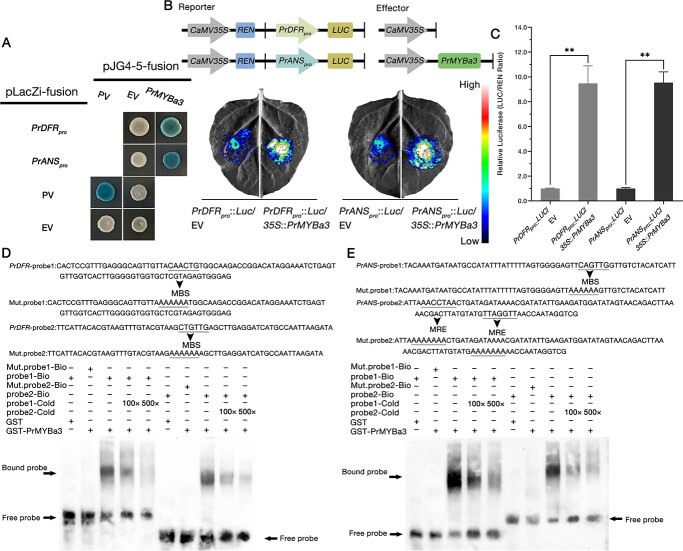
PrMYBa3 binds to the promoters of *PrDFR* and *PrANS* and activates their transcription. (**A**) Y1H assay showing that PrMYBa3 bound to the promoters of *PrDFR* (−1464 to −1 bp) and *PrANS* (−1500 to −1 bp). EV, empty vector; PV, positive vector. pLacZi-*AtLDOX_pro_* (−1702 to −1 bp) and pJG4–5-*AtPAP1* were used as the positive control. (**B**) Schematic representation of reporters and effectors (top), images of *N. benthamiana* leaves 3 days after co-infiltration (bottom). (**C**) Relative luciferase activities for co-expressed *PrDFR_pro_*::*LUC +* EV, *PrDFR_pro_*::*LUC + 35S*::*PrMYBa3*, *PrANS_pro_*::*LUC +* EV, and *PrANS_pro_*::*LUC + 35S*::*PrMYBa3*. Combinations containing EV were used as negative controls. Values represent mean ± standard deviation (*n* = 4). Asterisks indicate statistically significant differences (two-sided Student’s *t*-test; ^**^P < .01). (**D**) EMSA assay showed the binding of PrMYBa3 to the promoter of *PrDFR*. Two fragments containing MBS elements were used as probes. (**E**) EMSA assay showing the binding of PrMYBa3 to the promoter of *PrANS*. Two fragments containing MBS and MRE elements were used as probes. Mult. probe represents the mutated probe, in which the bases of MBS or MRE elements were all replaced by A. ‘+’ and ‘−’ indicate the presence and absence of the corresponding probe or protein, respectively. All the experiments were performed more than three times independently, with similar results.

Both *DFR* and *ANS* are crucial for flower pigmentation since they are the key structural genes in the anthocyanin late biosynthetic pathway. They are considered to convert dihydroflavonols to leucoanthocyanidins cooperatively, and then the colored anthocyanidin could be synthesized [52, 53]. According to the floral phenotype of *P. rockii* ‘Shu Sheng Peng Mo’ and our qRT–PCR analysis, we found that the differences in pigmentation between blotch and non-blotch areas may be closely correlated with the blotch-specific expression of *PrDFR* (TRINITY_DN207536_c0_g1) and *PrANS* (TRINITY_DN219185_c0_g1). Therefore, we cloned the promoter sequences of *PrDFR* and *PrANS* (Supplementary Data Fig. S9) to identify the transcription factors affecting these two LBGs. It was found that six and five members of the 12 PrMYBs bound to the promoters of *PrDFR* and *PrANS*, respectively, according to the Y1H assay results (Supplementary Data Fig. S10). Additional dual-LUC reporter assays revealed that only one of these PrMYBs activated the reporter gene through the promoters of both *PrDFR* and *PrANS* (Supplementary Data Fig. S11). The PrMYB was named PrMYBa3 (TRINITY_DN216927_c0_g1); according to the results of Y1H and dual-LUC reporter assays, PrMYBa3 works on the promoters of both *PrDFR* and *PrANS* (Fig. 5A–C). Further EMSA assays confirmed that PrMYBa3 binds to the promoter fragments of *PrDFR* and *PrANS* that contain MBS or MRE elements (Fig. 5D and E).

### Two PrbHLHs (PrbHLH1 and PrbHLH2) interact with PrMYBa3, acting as synergistic factors

**Figure 6:**
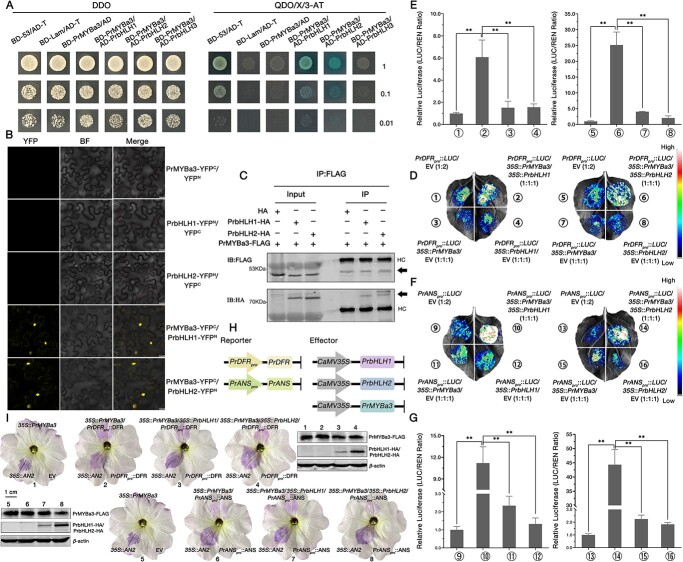
PrMYBa3 interacts with both PrbHLH1 and PrbHLH2 to activate the expressions of *PrDFR* and *PrANS* by protein complexes. (**A**) Y2H assays showing the interaction between PrMYBa3 and PrbHLH1/PrbHLH2 proteins. (**B**) BiFC assays showing the interaction between PrMYBa3 and PrbHLH1/PrbHLH2 proteins *in vivo*. Scale bars = 25 μm. (**C**) Co-IP assays showing the interaction between PrMYBa3 and PrbHLH1/PrbHLH2 proteins *in vivo*. (**D**, **F**) Images of *N. benthamiana* leaves 3 days after co-infiltration, showing that the double effectors enhanced activation of the *PrDFR* promoter and *PrANS* promoter. (**E**, **G**) Relative luciferase activities for co-expressed *PrDFR*/*ANS_pro_*::*LUC +* EV, *PrDFR*/*ANS_pro_*::*LUC + 35S*::*PrMBa3*, *PrDFR*/*ANS_pro_*::*LUC + 35S*::*PrMBa3 + 35S*::*PrbHLH1* or *PrDFR*/*ANS _pro_*::*LUC + 35S*::*PrMBa3 + 35S*::*PrbHLH2*, *PrDFR*/*ANS_pro_*::*LUC + 35S*::*PrbHLH1* or *PrDFR*/*ANS_pro_*::*LUC + 35S*::*PrbHLH2.* Values represent mean ± standard deviation (*n* = 4). Asterisks indicate statistically significant differences (two-sided Student’s *t*-test; ^**^P < .01). (**H**) Schematic representation of reporters and effectors used in transient overexpression of petunia. (**I**) Images of petunia ‘W59 × *axi*’ flowers 3 days after infiltration. *AN2*, which restored the mutant phenotype, was used as the positive control. When PrMYBa3-FLAG and PrbHLH1-HA/PrbHLH2-HA were transiently co-expressed, anthocyanin was observed to be accumulated. *PrDFR* and *PrANS* were used as reporters, respectively. Anti-FLAG and anti-HA antibodies were used to detect PrMYBa3 and PrbHLH1/PrbHLH2 proteins in ‘W59 × *axi*’ flowers. Scale bars = 1 cm.

Previous research claimed that the anthocyanin branch of flavonoid biosynthesis was commonly regulated by MBW (MYB-bHLH-WDR) transcription complexes [24, 28, 54–57]. In order to determine whether PrMYBa3 activates *PrDFR* and *PrANS* expressions solely or via protein complexes, we performed a phylogenetic analysis of all members of the bHLH family in petals of *P. rockii* ‘Shu Sheng Peng Mo’. Based on the RNA-seq data, a total of 53 full-length *PrbHLH* genes were identified. Three PrbHLHs were found to be clustered with members of the fifth subgroup (IIIf) in the phylogenetic tree, which was constructed from 196 bHLHs (143 from *Arabidopsis* and 53 from *P. rockii* ‘Shu Sheng Peng Mo’). SG5 (IIIf) was reported to be responsible for the regulation of anthocyanin biosynthesis (Supplementary Data Fig. S12) in plants [58–60]. We designated these three *PrbHLH* genes as *PrbHLH1* (TRINITY_DN212133_c0_g1), *PrbHLH2* (TRINITY_DN216979_c0_g1), and *PrbHLH3* (TRINITY_DN212853_c2_g1), then analyzed their transcriptional abundance and characteristic sequences (Supplementary Data Fig. S13A and B). The Y1H and dual-LUC reporter assays were firstly carried out to determine if PrbHLH1–3 could directly control *PrF3H*, *PrDFR*, and *PrANs*. The findings showed that PrbHLH1–3 were not able to bind to the promoters of these structural genes and may not have been able to control their transcription directly (Supplementary Data Fig. S14). Therefore, using the Y2H assay, we tried to confirm the relationship between PrbHLH1–3 and PrMYBa1–3. The findings demonstrated that none of these three PrbHLHs interacted with PrMYBa1 or PrMYBa2 in yeast cells (Supplementary Data Fig. S15A–D). However, PrbHLH1 and PrbHLH2 interacted with PrMYBa3, but not PrbHLH3 (Fig. 6A; Supplementary Data Fig. S15E). The interaction between PrMYBa3 and either PrbHLH1 or PrbHL2 *in vivo* was then validated by the BiFC assay (Fig. 6B). The Co-IP assays that transiently expressed PrMYBa3-FLAG and PrbHLH1-HA or PrbHLH2-HA in *N. benthamiana* leaves revealed that PrbHLH1 and PrbHLH2 were co-immunoprecipitated with PrMYBa3 (Fig. 6C). Our findings demonstrated that PrMYBa3 might interact with both PrbHLH1 and PrbHLH2 *in planta*.

We then conducted a multi-component dual-LUC reporter assay to investigate the functions of PrbHLH1 and PrbHLH2. *LUC* was driven by the promoters of *PrDFR* and *PrANS*, and *A. tumefaciens* carrying pSN1301-*PrMYBa3* and pSN1301-*PrbHLH1* or pSN1301-*PrbHLH2* plasmids were mixed 1:1 by volume for co-infiltration. Results showed that, compared with a single effector, the double effectors PrMYBa3-PrbHLH1 and PrMYBa3-PrbHLH2 activated *LUC* more efficiently (Fig. 6D–G). To confirm whether PrMYBa3 and PrMYBa3-PrbHLH1/2 complexes promote anthocyanin production *in vivo*, particularly in flowers, we transiently overexpressed PrMYBa3 and PrMYBa3-PrbHLH1/2 protein complexes in petunia ‘W59 × *axi*’ (*an2* – mutant). ‘W59 × *axi*’ has a floral transient transgene and recovery verification system that is technically immature and has the advantages of making it possible to verify the function of genes involved in anthocyanin biosynthesis. Firstly, *PrMYBa3* was transiently overexpressed in petals, then *PrDFR* and *PrANS* were driven by their own promoters and co-expressed with *PrMYBa3* or *PrMYBa3-PrbHLH1/2.* Results showed that *PrMYBa3* could not solely activate anthocyanin biosynthesis in flowers of ‘W59 × *axi*’. However, this was not the case when *PrMYBa3* was co-expressed with *PrDFR* or *PrANS*. Similar to the positive control (*AN2* overexpressed), an accumulation of anthocyanins was observed in infiltrated areas. Our findings demonstrated the effect of PsMYBa3 in activating the floral anthocyanin biosynthesis. Since the pigmentation of the petal did not significantly darken after the addition of PrbHLH1 or PrbHLH2, we speculate that the transformation of exogenous genes in petunia is limited. Consequently, we are unable to conclusively determine whether the synergistic factors worked in petunia ‘W59 × *axi*’ to improve the activation of anthocyanin biosynthesis. (Fig. 6H and I).

### Overexpression of *PrMYB*s and *PrbHLH*s promotes anthocyanin biosynthesis in corollas of tobacco

**Figure 7:**
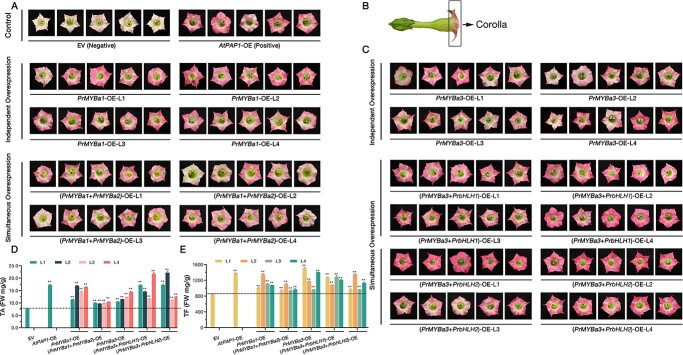
Analysis of transgenic tobacco plants. (**A**, **C**) Images of independent *T*_0_ transgenic lines. Each line is shown with five flowers. Empty vector was used as negative control and *AtPAP1* as positive control. (**B**) Corolla tissue definition. (**D**) Content of total anthocyanins (TA) in each transgenic line. (**E**) Content of total flavones and flavonols (TF) in each transgenic line. FW, fresh weight. For negative and positive controls, the corollas of different lines were mixed into one sample. Values represent mean ± standard deviation (*n* = 3). Asterisks indicate statistically significant differences (two-sided Student’s *t*-test; ^*^P < .05, ^**^P < .01).

To confirm the function of PrMYBa1, PrMYBa3, and their co-activators, we obtained transgenic tobacco plants, including lines that independently overexpressed *PrMYBa1* or *PrMYBa3* and simultaneously overexpressed *PrMYBa1* + *PrMYBa2* or *PrMYBa3* + *PrbHLH1*/*2*. Based on the expression level of transgenes, four independent *T*_0_ transgenic lines from each construct were chosen for further analysis (Supplementary Data Fig. S16A–C). Compared with the negative control, the corolla pigmentation of *PrMYBa1*-overexpressing (OE) lines showed obvious promotion under the same greenhouse culture condition. Unusually, anthocyanins were found unevenly distributed in the corolla, to be partially concentrated or to form a heart-shaped pattern in the corolla. However, in contrast to the *PrMYBa1*-OE lines, the corolla color of the *T*_0_ (*PrMYBa1* + *PrMYBa2*)-OE lines did not clearly intensify (Fig. 7A and B). HPLC analysis revealed that the contents of total anthocyanin (TA) and total flavone/flavonol (TF) were much higher in PrMYBa1-OE lines than in (*PrMYBa1* + *PrMYBa2*)-OE lines (Fig. 7D and E). The expressions of *NtF3H* and other EBGs were upregulated in *PrMYBa1*-OE lines and significantly upregulated in (*PrMYBa1* + *PrMYBa2*)-OE lines, according to the qRT–PCR analysis (Supplementary Data Fig. S16D). These results indicate that PrMYBa1 activated the expression of EBGs in tobacco flowers alone or in combination with PrMYBa2, with PrMYBa1 appearing to contribute to anthocyanin biosynthesis while PrMYBa2 did not promote anthocyanin production.

The *PrMYBa3*-OE lines were also visually different from the negative control, corollas displaying substantially deeper hues (similar to the positive control: *AtPAP1*-OE lines). The pigmentation was concentrated at the tips or edges of the petals, and some flowers even presented color patterns like pinwheels or five-pointed stars. However, the pigments were evenly distributed in the corollas of the *T*_0_ (*PrMYBa3* + *PrbHLH1*)-OE and (*PrMYBa3* + *PrbHLH2*)-OE lines, which had a dark pink color. The phenotype of lines with simultaneous overexpression of *PrbHLH1* and *PrbHLH2* implies that they may have promoted the pigmentation of the corolla. (Fig. 7C). According to the HPLC analysis, the single and double transgenic lines all had considerably higher TA and TF levels. When genes were simultaneously overexpressed, TA increased more while TF remained unchanged (Fig. 7D and E). According to the qRT–PCR analysis, PrMYBa3 and PrMYBa3-PrbHLH1 or PrMYBa3-PrbHLH2 complexes greatly promoted the expression of LBGs *NtDFR* and *NtANS* (Supplementary Data Fig. S16E). These results suggest that PrMYBa3 activated the expression of LBGs in tobacco flowers either alone or in combination with PrbHLH1 and PrbHL2, and PrbHLHs encouraged the accumulation of anthocyanins. The HPLC-DAD chromatograms of hydrolyzed extract of tobacco flowers are displayed in Supplementary Data Fig. S16F and G.

### DNA methylation of *PrF3H* and *PrANS* promoters was discrepant in blotch and non-blotch areas

**Figure 8:**
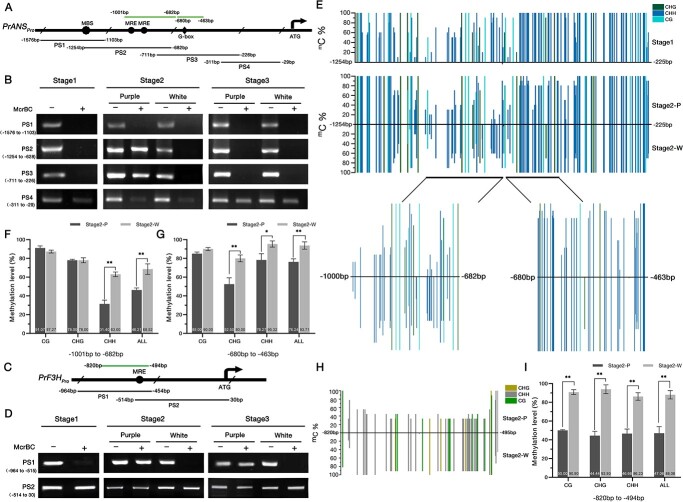
Cytosine methylation analysis of *PrANS* and *PrF3H* promoters in purple and white areas of petal. (**A**, **C**) Structural representations of the *PrANS* and *PrF3H* promoters. Fragments of ~500 bp were designated as PS1, PS2, PS3, and PS4, and their relative positions are marked. ATG represents the initiation codon; the relative positions of MBS and MRE elements are marked. (**B**, **D**) McrBC–PCR analysis of the *PrANS* and *PrF3H* promoters from S1 to S3. ‘+’ and ‘–’ indicate whether genomic DNA was treated with McrBC or not. Absence of strips implies high methylation of DNA. (**E**) Cytosine methylation level of *PrANS* promoter estimated by BSP. The two discrepant regions between the purple and white areas are enlarged and displayed at the bottom. The methylation percentage of each cytosine in the region is indicated by the vertical bars; data are presented as the mean. (**F**, **G**) Cytosine methylation levels of the −1001 to −628 bp and −680 to −463 bp regions in the *PrANS* promoter at S2. (**H**) Cytosine methylation level of region −820 to −495 bp in *PrF3H* promoter. Data are presented as the mean. (**I**) Cytosine methylation levels of the −820 to −495 bp region in the *PrF3H* promoter at S2. P and W represent purple and white areas of petal, respectively; ^m^C% represents the percentage methylation of each cytosine. CG, CHG, and CHH-type cytosine methylations are represented by lines with different colors. Values represent mean ± standard deviation (*n* = 3). Asterisks indicate statistically significant differences (two-sided Student’s *t*-test; ^*^P < .05, ^**^, *P* < .01).

To investigate the cause of the blotch-specific high transcription of *PrF3H*, *PrDFR*, and *PrANS*, we analyzed the expression patterns of the transcription factor genes mentioned above using qRT–PCR. The results revealed no clear relationship between the relative expression levels of transcription factor genes and structural genes. In detail, *PrF3H*, *PrDFR*, *PrANS *were strongly expressed in the blotch area, but *PrMYBs* and *PrbHLHs *were varied (Supplementary Data Fig. S17). Hence, we suspected that some factors would have an impact on the promoter functions of these anthocyanin structural genes in the white area of petals. As earlier studies have demonstrated, the DNA methylation which occurs at the carbon-5 position of cytosine (C) is widely distributed in the genome and impacts the expression of genes [38, 39, 61–63]. The differential expression of *PrF3H*, *PrDFR*, and *PrANS* in blotch and non-blotch areas may result from the methylation of their promoters; we thus performed DNA methylation analysis to verify this conjecture. The methylation of *PrANS_pro_* during flower development stages S1–S3 was first qualitatively detected by a methylation-specific endonuclease (McrBC)–PCR. The result showed that the degrees of methylation in the regions PS2 (−1254 to −628 bp) and PS3 (−711 to −226 bp) were quite different in purple and white areas at S2. However, the methylation of regions PS1 (−1576 to −1103 bp) and PS4 (−311 to −29 bp) showed no difference. This result implied a hypermethylation of the *PrANS* promoter in the non-blotch area during flower development (Fig. 8A and B). The result of *PrF3H_pro_* analysis showed that PS1 (−964 to −515 bp) was highly methylated in the white area, while PS2 (−514 to −30 bp) did not differ significantly between the two areas (Fig. 8C and D). Interestingly, we observed that DNA methylation seemed to have occurred since the early stage, in which anthocyanins had not begun to accumulate.

To verify this result, we analyzed the cytosine methylation level of *PrANS_pro_* and *PrF3H_pro_* using the bisulfite sequencing PCR (BSP) method. At S1 and S2, the *PrANS_pro_* PS1 (−1576 to −1103 bp) and PS4 (−311 to −29 bp) regions showed hypermethylation in both the white and purple areas of the petal. At S2, the regions −1001 to −682 bp and −680 to −463 bp, which belong to PS2 and PS3 respectively, showed a fairly pronounced difference in the purple and white areas (Fig. 8E). In the white area, 68.52% of the cytosines were methylated in the region −1001 to −682 bp and 93.71% in the region −680 to −463 bp, while only 46.21 and 76.24% of the cytosines were methylated in the two regions in the purple area. At S2, the CG- and CHG-type (H = A, C, or T) methylation levels of the *PrANS_pro_* −1001 to −682 bp region had no significant difference in the purple and white areas, while the CHH-type and total methylation levels were 31.6 and 22.3% higher in the white area than in the purple area (Fig. 8F). In the region −680 to −463 bp, only the CG-type methylation levels had no significant difference in the two areas; the CHH-, CHG- type, and total methylation levels in the white area were 27.5, 17, and 17.5% higher than those in the purple area, respectively (Fig. 8G). The raw data are presented in Supplementary Data Table S3. In order to investigate the variety of DNA methylation in *PrANS_pro_* during flower development, we detected the methylation levels of these two regions (−1001 to −682 bp and − 680 to −463 bp) in petals of S1, S2, and S3. The results indicated that the CHG-type, CHH-type, and total methylation levels of the two regions decreased in the purple area from S1 to S2 (Supplementary Data Fig. S18), then returned to a higher level close to S1 at S3. The results suggested that methylation levels of regions −1001 to −682 bp and −680 to −463 bp in *PrANS_pro_* were modifiable during flower development.

Analogously, we found that the methylation level of the region −820 to −495 bp, which belongs to PS1 of *PrF3H_pro_*, was different between the purple and white areas of the petal (Fig. 8H). The total methylation level of the region in the white area was 41% higher than that in the purple area, and the CG-, CHG-, and GHH-type cytosine methylation levels were all nearly doubled in the white area (Fig. 8I). We suggest that the DNA methylation of *PrANS* and *PrF3H* promoters might be associated with their silencing in the non-blotch area of petals, which is a potential mechanism to regulate the formation of the petal pigmentation pattern in Xibei tree peony.

## Discussion

For years, researchers have been intrigued by how petal blotches are formed in tree peony. As a preceding study indicated, the differential accumulation of flavonoids in two areas of a petal is an immediate cause [16]. However, the molecular mechanism remains undiscovered. In this study, we characterized three MYB and two bHLH transcription factors that activate the expression of anthocyanin structural genes. We found that the hypermethylation of structural gene promoters in the white area could be a potential regulatory mechanism associated with the formation of petal blotches in Xibei tree peony.

### Spatiotemporal expressions of structural genes result directly in pigmentation patterning

Depending on the phenotype observed, the blotch appeared for the first time on petals during S1 to S2 (Fig. 1A). The expression of key structural genes, such as *PrCHS*, *PrCHI*, *PrFNSII*, *PrF3H*, *PrDFR*, and *PrANS*, rose rapidly from S1 to S2 (Supplementary Data Fig. S2). The accumulation of flavonoids also increased rapidly during S1 to S2, but lagged slightly behind gene expression (Fig. 1B and C). Clearly, the expression of these genes was closely related to the accumulation of flavonoid products in petals.

Significant differences exist in the accumulation of flavonoids in the blotch and non-blotch areas of the petal, which is the common characteristic of Xibei tree peony [16]. It was obvious that differences in anthocyanin composition or content determined the different colorations of the two petal areas. In the typical Xibei tree peony cultivar ‘Shu Sheng Peng Mo’, anthocyanin products accumulated only in the blotch area, which is convenient for detecting and identifying genes that associated with anthocyanin biosynthesis. Virtually all flavonoid biosynthesis structural genes in the petals of *P. rockii* ‘Shu Sheng Peng Mo’ were identified from the RNA-seq database based on the annotation. In addition to the *PsCHS* we have previously reported [32], only *PrF3H* (TRINITY_DN127818_c0_g1), *PrDFR* (TRINITY_DN207536_c0_g1), and *PrANS* (TRINITY_DN219185_c0_g1) showed blotch-specific high transcription. In the anthocyanin biosynthetic pathway, the reaction for the production of dihydroflavonol is one of the important branches of anthocyanin biosynthesis, which depends on F3H catalysis. The continuous catalytic reactions by DFR and ANS are essential for anthocyanin products, and therefore the spatiotemporal differential expression of these structural genes is most likely to be the immediate cause of blotch formation. For the non-blotch area, flavones rather than flavonols are the main incremental products, and therefore *PrFNSII* (TRINITY_DN227426_c16_g1) is a key structural gene worth considering. Since flavone biosynthesis is in competition with anthocyanin biosynthesis for common intermediates, the anthocyanin deficiency in the non-blotch area implied a potential precise distribution of metabolic flux in the two areas of the petal. We speculate that *PrFNSII* and *PrF3H-2* (TRINITY_DN190073_c1_g1), which is a contrary expression model of *PrF3H*, serve the early biosynthetic pathway in the non-blotch area.

The relationship between the differential flavonoid accumulation and structural gene expressions was confirmed through spatial and temporal expression analyses. To disentangle the molecular basis of blotch formation in tree peony, we should first investigate why the key structural genes are differently expressed in blotch and non-blotch areas of the petal.

### Regulation of flavonoid biosynthesis by R2R3-MYB in Xibei tree peony is conservative

We have previously attempted to identify the transcription factors involved in flavonoid biosynthesis by comparing DEGs between blotched and non-blotched areas. However, the difference was less pronounced, and *PsMYB12* was the only ideal candidate gene for further research. In this study, we firstly verified whether PsMYB12 acts on promoters of the three structural genes mentioned above, but the findings revealed that it had no influence on either their activation or inhibition (Supplementary Data Figs S7A, S10, and S19). Moreover, PsMYB12 demonstrated specificity in phylogenetic analyses [32]. Consequently, we reasoned that it would be worthwhile to further discuss whether PsMYB12 directly controls anthocyanin biosynthesis.

In this study, our strategy for gene screening was different. As has been widely investigated, many R2R3-MYBs are involved in the regulation of flavonoid biosynthesis. In angiosperms, SG7 of MYB regulators controls flavonol biosynthesis [64], SG6 controls anthocyanin biosynthesis [65], SG5 controls proanthocyanidin and anthocyanin biosynthesis [6, 66], and SG4 encodes transcriptional repressors [67]. Protein structure and phylogenic analysis would help to predict the functions of newly discovered MYBs, because members with analogous protein structures and biological functions were typically clustered within the same subgroups. We firstly identified PrMYBa1as an SG7 R2R3-MYB regulator in *P. rockii* ‘Shu Sheng Peng Mo’ (Supplementary Data Fig. S20), which directly combines with the promoter of EBG *PrF3H*, and solely activate the transcription of *PrF3H* strongly (Fig. 3). We characterized *PrMYBa1* as the key gene for the regulation of the anthocyanin early biosynthetic pathway. PrMYBa2, which is a member of SG5, is the second R2R3-MYB discovered to activate *PrF3H *(Supplementary Data Fig. S20). Our research suggests that PrMYBa2 does not appear to be a potent activator of the target gene *PrF3H*. (Supplementary Data Fig. S7). We concentrated on the SG6 members for anthocyanin biosynthesis. PrMYBa3 was found to be the only reliable transcription factor clustered with the members controlling anthocyanin biosynthesis in different tissues of *Arabidopsis* (AtMYB75/PAP1, AtMYB90/PAP2, AtMYB113, and AtMYB114) (Supplementary Data Fig. S3) [29, 55]. Our findings demonstrated that PrMYBa3 promotes the expression of both *PrDFR* and *PrANS*, which may influence the accumulation of anthocyanin compounds in petals (Fig. 5).

In *Arabidopsis*, EBGs are activated by functionally redundant R2R3-MYB regulators (MYB11, MYB12, MYB111), whereas the activation of LBGs requires a transcription complex composed of MYB-bHLH-WD40 [55–57]. The conserved motif [D/E]Lx2[R/K]x3Lx6Lx3R of PrMYBa1, PrMYBa2, and PrMYBa3 (Supplementary Data Fig. S4B) implied that they could combine with the bHLH regulators. The interactions between PrMYBa3 and PrbHLH1/2 were validated by a series of assays in our study (Fig. 6, Supplementary Data Fig. S15E), however, PrMYBa1 and PrMYBa2 did not interact with any PrbHLHs (Supplementary Data Fig. S15A–D). This result suggests that, as with the *Arabidopsis* system, the anthocyanin early biosynthetic pathway of tree peony may depend solely on regulation by the MYB family. The cooperative regulation of LBGs by PrMYBa3 and PrbHLHs reveals the conservative function of ‘MB’ transcription complexes in the anthocyanin branch of the Xibei tree peony.

### Functions of synergistic factors

According to the results of assays *in vitro* (Fig. 3), PrMYBa2 acts as a synergistic factor of PrMYBa1 to activate the EBGs, while the interaction of PrMYBa1 and PrMYBa2 seems not to be essential. In comparison, the PrMYBa1-PrMYBa2 complex has a stronger activation effect on *PrF3H* than a single PrMYBa1 or PrMYBa2 (Fig. 4). However, the anthocyanin accumulation of *T*_0_ (*PrMYBa1* + *PrMYBa2*)-OE transgenic tobacco lines were less than those of *PrMYBa1*-OE lines. We suspect that PrMYBa2 is a co-activator that contributes to the early biosynthetic pathway, and has the potential to regulate anthocyanin biosynthesis indirectly. PrMYBa2 grouped with AtMYB123 (TT2), which acts as a key determinant for the synthesis of proanthocyanidins in *Arabidopsis* seeds (Supplementary Data Fig. S3) [66]. Therefore, the addition of PrMYBa2 to tobacco may contribute to downstream proanthocyanidin biosynthesis and reduce anthocyanin accumulation in the corolla. Currently, whether PrMYBa2 is a target for the biosynthesis of anthocyanin or proanthocyanidin has not yet been determined, but it is clearly an activator of the flavonoid early biosynthetic pathway in Xibei tree peony. The main functions of PrMYBa2 are expected to be explored in the future.

The bHLH SG5 (IIIf) members PrbHLH1 and PrbHLH2 were found to form heterodimers with PrMYBa3 and strongly activate the expressions of the LBGs *PrDFR* and *PrANS*. However, it appears that the interaction is non-essential for PrMYBa3 in the activation of LBGs, according to the assays *in vitro* and in tobacco (Figs 5 and 7C). So what is the primary function of bHLH, especially in the process of blotch formation? As previously reported, the formation of some pigmentation patterns is simply dependent on positional information. Examples include the molecular basis of stripe or venation formation in snapdragon and petunia flower, which was found to be a conserved process based on the overlapping expression domains of *R2R3-MYB* and *bHLH* genes, in which bHLH factors particularly provided the epidermal specificity [68–70]. In our study, PrbHLH1 and PrbHLH2 were discovered to promote the accumulation of anthocyanin products in plants (Figs 6 and 7). As observed, the corolla color of *T*_0_ (*PrMYBa3* + *PrbHLH1*)-OE and (*PrMYBa3* + *PrbHLH1*)-OE lines differs significantly from that of *PrMYBa3*-OE lines in transgenic tobacco. Through phenotypic observation on a large quantity of materials, we discovered that the anthocyanin products appeared more evenly distributed in the epidermis of the corolla. This is a fascinating finding, which opens up for discussion the topic of the function of bHLH factors in pigmentation patterning. We have confirmed that the activation of SG6 MYB in anthocyanin biosynthesis does not necessarily rely on the synergistic effect of bHLH and other complex members. The synergistic factor, for example, functioning as a wizard to offer positional information, may play a crucial part in the formation of color patterns. Nevertheless, the petal blotch of Xibei tree peony is not exactly the same as the pigmentation patterns of other species. Therefore, the molecular mechanism behind the floral color pattern may not necessarily be universal.

### DNA methylation may mediate gene silencing and be associated with blotch formation

According to the qRT–PCR analysis, *PrMYBa1*, *PrMYBa3*, and the synergistic factor genes *PrMYBa2*, *PrbHLH1*, and *PrbHLH2* did not express regularity between blotch and non-blotch areas during S1–S5 (Supplementary Data Fig. S17). The active expressions of *PrMYBa1* and *PrMYBa2* in early S1 may be related to their regulation of the early biosynthetic pathway. However, their relative expression is higher in the non-blotch than in the blotch area. As the key activator of the anthocyanin late biosynthetic pathway, *PrMYBa3* displayed a temporal expression trend comparable to that of *PrDFR* and *PrANS*, while the spatial expression pattern was the opposite. The spatiotemporal expression patterns of structural genes in response to these activators appear to be challenging to understand. Therefore, it is worthwhile investigating the reason why these structural genes are silenced in non-blotch areas. Flower color and aroma are considered to be under a synergistic regulation mechanism. It was reported that MYB controls the production of flavonoids and volatile benzenoid-phenylpropanoids in a synergistic manner [64, 71, 72]. Consequently, the potential functions of *PsMYBs* in aromatic metabolism might be implied by their special spatial expression, particularly *PsMYBa3*.

As expected, the methylation levels of the *PrANS_pro_* regions −1001 to −682 bp and −680 to −463 bp were significantly higher in the white area than in the purple area. In the white area, the CHH-type methylation level was higher in the −680 to −463 bp region and the CHG-type methylation level was higher in both the −1001 to −682 bp and −680 to −463 bp regions (Fig. 8E). Given that the two regions of the *PrANS* promoter contain three MYB binding sites and a G-box element, we suspect that the silence of *PrANS* in non-blotch areas may result from the methylation modification. Similar findings were found in the analysis of the *PrF3H* promoter; the −820 to −495 bp region, which contains the MRE element revealed higher CG-type, CHG-type, and CHH-type methylation levels in the white area (Fig. 8H). It is reported that the DNA methylation level changes dynamically during development to provide a maximal beneficial environmental response or reproductive and developmental regulation *in planta* [35, 73–76]. We discovered that methylation of the *PrANS* promoter clearly decreased from S1 to S3, and the dynamics of DNA methylation implied a process of demethylation during S1 and S2. Therefore, based on the accumulation of anthocyanins, we speculate that a demethylation during S1–S2 may have restored the expression of *PrANS* in the blotch area. During the growth of the flower, demethylation and gradual remethylation may alternate, leading to the localized differential expression of anthocyanin structural genes in the petal. Our results suggest that epigenetic modification plays a role in the regulation of anthocyanin biosynthesis, which may directly contribute to the development of the blotch. To confirm the influences of DNA methylation in *PrF3H*, *PrANS*, and other structural genes of Xibei tree peony, more experiments and analyses are required in future studies.

### Conclusions

In Xibei tree peony *P. rockii* ‘Shu Sheng Peng Mo’, the key structural genes *PrF3H*, *PrDFR* and *PrANS* are only strongly expressed in the blotch area of petal, the silence of *PrDFR* and *PrANS* in the non-blotch area directly prevents the accumulation of anthocyanin products. We confirmed that the PrMYBa1-PrMYBa2 and PrMYBa3-PrbHLH1 or PrMYBa3-PrbHLH2 complexes controlled these EBG and LBGs. However, the distinct spatiotemporal expression patterns of the *PrF3H* and *PrANS*in blotch and non-blotch areas might be caused by DNA methylation modification of their promoters.The methylation levels of the *PrANS* and *PrF3H* promoters were found to be significantly different in the purple and white areas of petals at S2, which is the key period for blotch formation.

**Figure 9:**
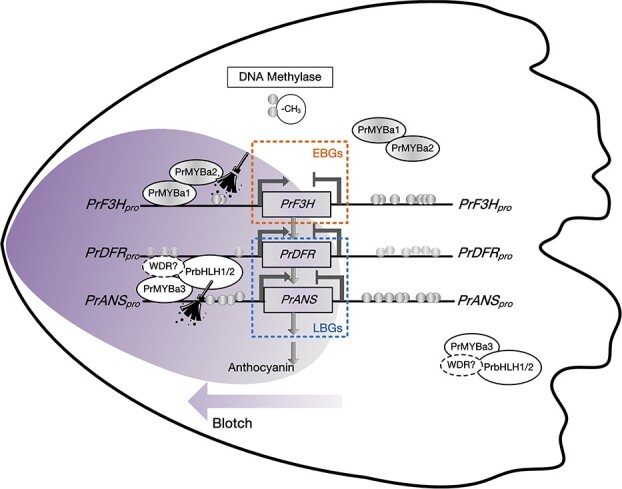
. A model showing the potential molecular mechanism of blotch formation in *P. rockii* ‘Shu Sheng Peng Mo’. High methylation of promoters represses the transcription of anthocyanin EBG *PrF3H* and LBGs *PrDFR* and *PrANS* in non-blotch areas, while the elimination of methylation in the blotch area at S2 facilitated their activation by the PrMYBa1-PrMYBa2 complex and the PrMYBa3-PrbHLH1 or PrMYBa3-PrbHLH2 complexes. As a result, anthocyanin products failed to accumulate in the non-blotched area, causing blotch formation. Thick gray downward arrows indicate the simplified reaction flow of anthocyanin biosynthesis, ovals represent the transcription factors identified, balls indicate -CH_3_ (methyl), and the right arrows and T-shapes indicate normal and repressive expression, respectively.

Throughout flower development, the methylation level of *PrANS* promoter is fluctuant, from S1 to S2 there was a definite reduction. The reduced methylation of structural gene promoters may be associated with the blotch formation in Xibei tree peony (Fig. 9).

## Materials and methods

### Plant materials

The Xibei tree peony cultivar *P. rockii* ‘Shu Sheng Peng Mo’ sampled in our study was cultivated at the Guo Se Tree Peony Garden (Yanqing, Beijing, China). Petals were divided into purple and white areas for analysis, except for the S1 samples (Fig. 1A). Tobacco (*N. benthamiana*) and petunia ‘W59 × *axi*’ used for dual-LUC reporter assays and transient expression assay were grown in greenhouses using artificial light close to natural light at 25°C. Transgenic plants of the early-flowering tobacco cultivar ‘Xinashi’ (*Nicotiana tabacum*) were used in the gene overexpression experiments [77].

### HPLC analysis

Methods for flavonoid extraction and analysis were as previously described [16]. Dried petals were ground into powder; 0.2 g of the powder was dipped in 2% formic acid/methanol (v/v) solution and sonicated for 30 minutes, then centrifuged. Extraction was repeated two or three times and the supernatant was collected and filtered with a 0.22-μm membrane. The flavonoids were quantified using an Agilent 1100 HPLC with a Dionex-DAD; 0.1% formic acid (A) and acetonitrile (B) were used as the mobile phases, and the program was as follows: 0–30 minutes (5–23% B), 30–33 minutes (23–24% B), 33–35 minutes (24–25% B), 35–38 minutes (25–90% B), 38–40 minutes (90–5% B). Cyanidin 3-*O*-glucoside (Cy3G) and rutin were used as standards for linear regression and to build calibration curves. The same system was used for the analysis of the transgenic tobacco plants used. Triple technical replicates were analyzed for each sample.

### Transcriptome sequencing and analyses

Petal samples at S1, S3, and S5 (Fig. 1A) were collected and separated into white and purple areas for and RNA-seq. The Ion Plus Fragment Library Kit (Life Technologies) and the QIAquick Gel Extraction Kit (Qiagen, USA) were used to construct and purify the cDNA libraries, respectively. For quantitative analysis of the effective concentration of the library we used qPCR (effective concentration >2 nM). The libraries were sequenced on an Illumina HiSeq platform. FastQC was used to assess the raw data, and the per base quality was >Q30. Raw data were filtered to obtain high-quality clean data, and the GC contents of the samples were kept at a similar normal distribution, to ensure uncontaminated sequencing data. Then, clean data were used for sequence assembly to obtain the unigene library of these samples. Postcorrection accuracy of >99% was used as the criterion for high-quality full-length transcripts. The coding region sequences (CDSs) of unigenes and the corresponding amino acid sequences were predicted using TransDecoder software. After prediction, the predicted open reading frame (ORF) was compared with known protein databases Pfam [78] and Swiss-Prot [79], and the most likely reliable CDSs encoding the proteins were selected.

BLAST [80] software was used to analyze unigene sequences with the NR, Swiss-Prot, GO [81], COG/KOG [82], and KEGG [83] databases, and KOBAS2.0 [84] was used to obtain KEGG orthology results of unigenes in KEGG. Annotation information on unigenes was obtained using HMMER software and the Pfam database. Gene expression levels were measured based on the FPKM value. The parameters of fold change >2 and q value <0.001 were used as criteria for evaluation of DEGs.

### Identification of *R2R3-MYB*, *bHLH* genes and phylogenetic analysis

To identify the MYB domain-containing sequences, we constructed a local database using the assembled unigenes, and the HMM profile (PF00249) was used to isolate all homologs with HMME 3.0. Then, redundant or incomplete sequences and eliminated sequences that did not contain the R2R3-MYB domain were removed.

Protein sequences were aligned using ClustalW and full-length sequences of the MYB proteins and bHLH proteins were used to generate phylogenetic trees based on the neighbor-joining method using MEGA7.0 software. The tree nodes were evaluated with 1000 bootstrap replicates. All sequences of *Arabidopsis* were retrieved from the NCBI database; all the gene IDs are shown in Suppledmentary Data Figs S3 and S11.

### qRT–PCR analysis

qRT–PCR was performed using the SuperReal PreMix Plus (SYBR Green) Kit (TIANGEN, China) on a Step One Plus™ Real-Time PCR system (Applied Biosystems). The analysis of each sample included three biological replicates and three technical replicates. Relative quantification was obtained using the 2^−ΔΔCt^ method [85], and *β*-*tubulin* (accession number EF608942) was used as the internal control. Transgenic tobacco flowers were analyzed using the same method. Primers are listed in Supplementary Data Table S4.

### Isolation of promoters by genome walking

Due to the lack of genomic information, the promoters of *PrF3H*, *PrDFR*, and *PrANS* were cloned with the method of genome walking PCR using the GenomeWalker kit (TaKaRa, Japan). Specific primers were designed based on the reference sequence. The amplified PCR products were cloned into pMD18-T (TaKaRa, Japan) for subsequent sequencing. Finally, a pair of primers were designed to verify the correctness of the promoter sequence. Primers used are listed in Supplementary Data Table S4.

### Yeast one-hybrid assay

The CDSs of *PrMYB*s and *PrbHLH1*, *PrbHLH2*, and *PrbHLH3* were ligated into the pJG4-5 vector to generate 15 prey constructs. The three promoters of *PrF3H*, *PrDFR*, and *PrANS* containing conserved *cis*-elements were ligated into the pLacZi vector to generate three bait constructs. Paired prey and bait constructs were co-transformed into yeast strain EGY48 cells. Then, microbial medium without tryptophan and uracil but containing X-gal was used for blue color development [86]. Primers used are listed in Supplementary Data Table S4.

### Electrophoretic mobility shift assay

EMSA was carried out with recombinant PrMYBa1-GST and PrMYBa3-GST proteins purified from *E. coli*. We firstly synthesized oligonucleotide probes that were labeled with biotin marker or unlabeled, and used the unlabeled probes as cold competitors and the mutated probes as controls. Then, the Light Shift™ Chemiluminescent EMSA Kit (Thermo Fisher, USA) was used to perform EMSAs, using the steps previously described [87]. Chemiluminescence was detected by the FluorChem R Imaging System (Protein Simple, USA). Primers used in our EMSA are listed in Supplementary Data Table S4.

### Dual luciferase reporter assay

The CDSs of each *PrMYB* and *PrbHLH* were cloned and ligated into the pSN1301 vector to construct effectors, and the promoters of *PrF3H*, *PrDFR*, and *PrANS* were ligated into pGreenII-0800-LUC vector to generate reporters. The paired effector and reporter were co-transfected into *N. benthamiana* leaves using the *Agrobacterium*-mediated approach [88, 89]. LUC and REN activities were analyzed using the Dual Luciferase^®^ Reporter Assay System (Promega, USA) and a GloMax^®^ 20/20 Luminometer (Promega, USA). The luciferase complementation imaging assay was performed using the Tanon 5200 Multi Imaging System (Tanon, China). The primers used in our LUC assays are listed in Supplementary Data Table S4.

### Yeast two-hybrid assay

The CDS of each *PrMYB* and *PrbHLH* was cloned into either pGADT7 or pGBKT7 vector to generate prey or bait constructs. The prey and bait were co-transformed into Y2HGold cells. Transformed yeast cells were dotted on SD medium lacking Trp and Leu (DDO), or lacking Trp, Leu, His, and Ade, but supplemented with X-*α*-gal and 3-AT (QDO/X/3-AT), with a 10× dilution series. Primers used in our Y2H assays are listed in Supplementary Data Table S4.

### Bimolecular fluorescence complementation assay

PrMYBa1and PrMYBa2, PrMYBa3 and PrbHLH1–3 were fused with the N-terminus or C-terminus of YFP for the BiFC assay, and then A-YFP^N^ and B-YFP^C^ were co-transfected into *N. benthamiana* leaves by *A. tumefaciens* [90]. A confocal laser scanning microscope (Leica TCS SP2, Leica, Germany) was used to observe the fluorescence signals. Primers used are listed in Supplementary Data Table S4.

### Pull-down assays

The fused PrMYBa1-His, PrMYBa2-GST, and GST proteins were expressed in *E. coli* cells and purified using Ni-NTA HisBind^®^ Resin (Millipore, USA) and ProteinIso™ GST Resin (Transgen, China) following the manufacturers’ protocols. The purified PrMYBa1-His and PrMYBa2-GST were incubated in binding buffer (0.1 M phosphate, 0.15 M NaCl, pH 7.2) for 4–5 hours at 4°C, and then the protein blend was incubated with GST resin (beads should wash sufficiently with the binding buffer) for 1–2 hours at 4°C. After washing and denaturation, the bound proteins were analyzed by western blotting using anti-His antibody and anti-GST antibody (KangWei, China). Primers used are listed in Supplementary Data Table S4.

### Co-immunoprecipitation assay

For co-immunoprecipitation assays, *A. tumefaciens* carrying PrMYBa1-GFP and PrMYBa2-FLAG, PrMYBa3-FLAG and PrbHLH1-HA or PrbHLH2-HA vector was co-infiltrated into *N. benthamiana* leaves. Three days after infiltration the leaves were homogenized with extraction buffer. The supernatant was incubated with anti-FLAG (Millipore, USA) antibody for 2 hours at 4°C, and then precipitated with 20 μl of Protein A/G Mix Magnetic Beads (Millipore, USA). The beads were washed with TBS buffer. After washing and denaturation, the bound proteins were analyzed using anti-GFP (Lablead, China), anti-FLAG (Millipore, USA), and anti-HA (KangWei, China) antibodies. Primers used in our Co-IP assays are listed in Supplementary Data Table S4.

### Plant transformation

For transient expression in petunia, the CDS of each *PrMYB* fusion with FLAG tag and *PrbHLH* fusion with HA tag was ligated into the pSN1301 vector to construct effectors. The sequences of *PrF3H*, *PrDFR*, and *PrANS* promoters were linked to the CDS of relevant gene and ligated into pCambia1302 vector to generate reporters. All constructed vectors were introduced into GV3101, and then the paired effector and reporter were co-transfected into petunia petals according to the experimental design. The *A. tumefaciens*-mediated transient transformation of petals was performed as described previously [91]. Phenotypes were observed 3 days later.

For transforming tobacco, the CDS of each *PrMYB* was ligated into the pSN1301 vector independently or ligated into the pCambia1302 vector with *PrbHLH* simultaneously. The genetic transformation of tobacco with *PrMYB*s and *PrbHLH*s was accomplished according to previous methods [32]. Hygromycin was used to screen the transgenic plants. Positive transgenic lines were identified by genomic PCR, and then the corollas of transgenic plants were used for further analysis by qRT–PCR and HPLC. Primers used are listed in Supplementary Data Table S4.

### McrBC–PCR and bisulfite sequencing analysis

McrBC cleaves DNA containing methylcytosine on one or both strands. DNA sites recognized by McrBC consist of two half-sites of the form (G/A)^m^C. For McrBC–PCR analysis, 1-μg genomic DNA samples of high quality isolated from petals (separated into purple and white areas) was digested using McrBC (NEB, USA) for 6 hours at 37°C. Complete digestion of the DNA sample being essential, the supplied 4.3 kb linear control DNA was used to indicate the extent of digestion. After being thoroughly digested, the methylated control plasmid DNA produced several fragments between ~700 and 2.3 kb in size (Supplementary Data Fig. S21A). For each sample, the DNA, digested or not, was used as the template for semiquantitative PCR analysis. If the template was cleaved, the yield of PCR products would be lower or nonexistent. We estimated the level of DNA methylation based on this principle, and made a comparison between blotch and non-blotch areas. The yield of amplification products was determined by electrophoresis on 1% agarose gel. The experiments were conducted with three biological replicates. Promoters of *PrANS* and *PrF3H* were divided into four (PS1, PS2, PS3, PS4) and two (PS1, PS2) fragments ~500 bp in size, respectively, and then amplified using specific primers (Supplementary Data Table S5).

BSP was conducted according to the method described previously [39]. 500 ng of high-quality genomic DNA isolated from purple and white areas of petals were treated using the EZ DNA Methylation Gold Kit (Zymo Research, USA) strictly according to the manufacturer’s instructions. Fragments of *PrANS* (−1254 to −225 bp) and *PrF3H* (−820 to −495 bp) promoters were divided into several fragments ~250 bp in size. Then, these fragments were amplified with the respective degenerate primers and using the bisulfite-treated DNA as templates (Supplementary Data Table S6). The PCR products were purified and ligated to pMD18-T vector for sequencing, and 10 clones of each fragment were analyzed using the Kismeth (https://katahdin.girihlet.com/kismeth/revpage.pl) online software. Experiments were conducted with three biological replicates.

To ensure sufficient CT conversion efficiency, we used Lambda DNA (Thermo Fisher, USA) as a control. Lambda DNA was processed in conditions identical to those used with the experimental DNA samples, then employed as the template for a PCR. Two fragments were amplified using normal primers, fully transformed primers (forward C to T, reverse G to A), and degenerated primers (forward C/T to Y, reverse A/G to R), respectively. As a sign of complete transformation, the PCR efficiency from high to low should be fully converted primers, degenerated primers, and normal primers. The control for estimating the BS conversion efficiency was performed as an independent experiment; the products detected by agarose gel electrophoresis are shown in Supplementary Data Fig. S21B. According to the results, the CT conversion of Lambda DNA was found to be sufficient, and the control partially validated our method for BSP. Primers used for the control experiment are listed in Supplementary Data Table S6.

## Acknowledgements

This work was supported by the National Natural Science Foundation of China (No. 32030095). We are particularly grateful to Professor Chunhong Yang (Institute of Botany, Chinese Academy of Sciences) for language polishing. We thank Professor Xiaofeng Cao (Institute of Genetics and Development, Chinese Academy of Sciences) for kindly providing guidance on experimental technique. We also thank the anonymous reviewers and editors for comments that greatly improved the manuscript.

## Author contributions

L.S.W. and J.Z. conceived and planned the study. J.Z. performed the research. Y.Z.W. and Q.Y.W. helped conduct some of the experiments, the remaining six authors also contributed in various ways. L.S.W. and J.Z. wrote the manuscript. All authors read and approved the final manuscript.

## Data availability

All relevant data can be found within the manuscript or the supplementary materials. All data and material reported in this manuscript are available from the corresponding author upon request.

## Conflict of interest statement

The authors declare that they have no competing interests.

## Supplementary data

Supplementary data are available at *Horticulture Research* online.

## Supplementary Material

Web_Material_uhad100
