# Supplementary material for: The combination of DNA methylation and positive regulation of anthocyanin biosynthesis by MYB and bHLH transcription factors contributes to the petal blotch formation in Xibei tree peony
Source: Hortic Res. 2023 May 19;10(7):uhad100. doi: 10.1093/hr/uhad100 (PMC10327543; doi:10.1093/hr/uhad100)
Supplement: Web_Material_uhad100 [file web_material_uhad100.zip › supplementary Fig.S5.docx]

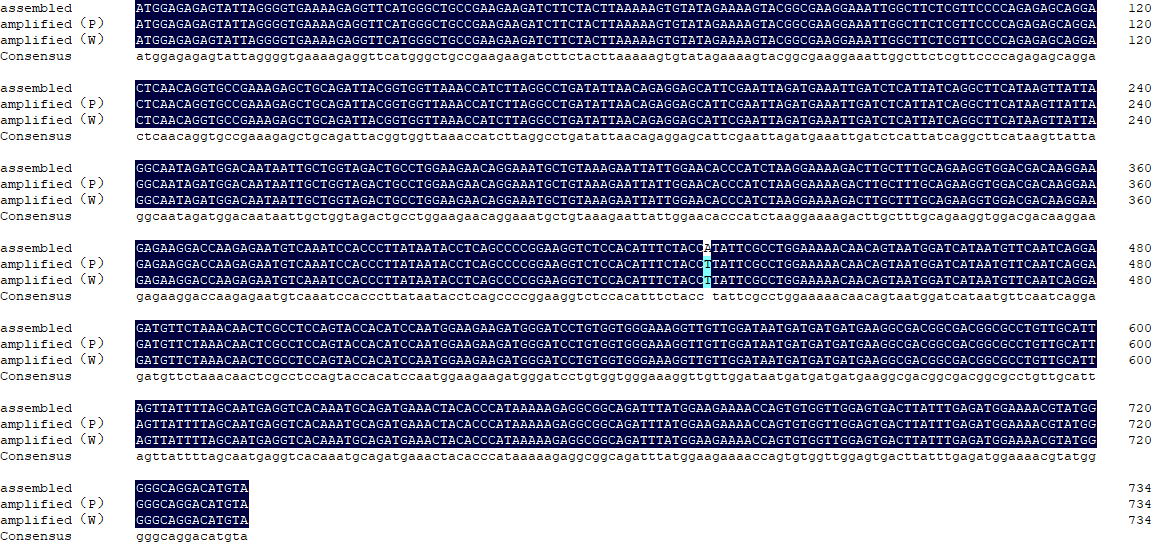
TRINITY_DN216927_c0_g1


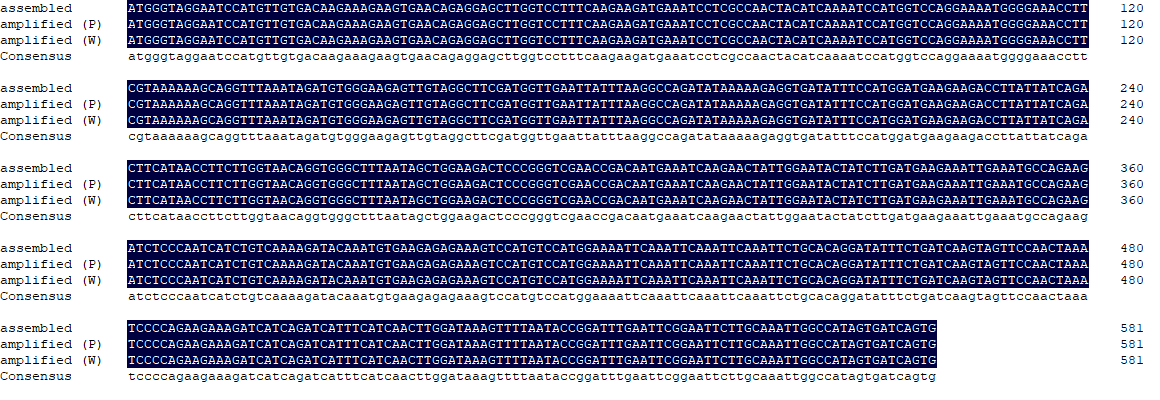
TRINITY_DN184394_c0_g1


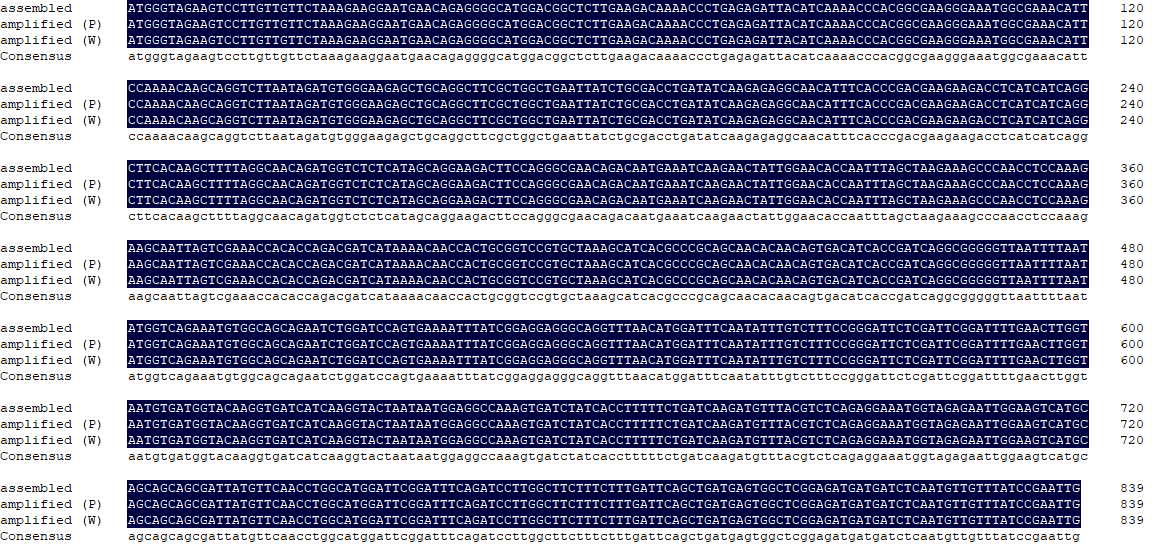
TRINITY_DN210213_c0_g1


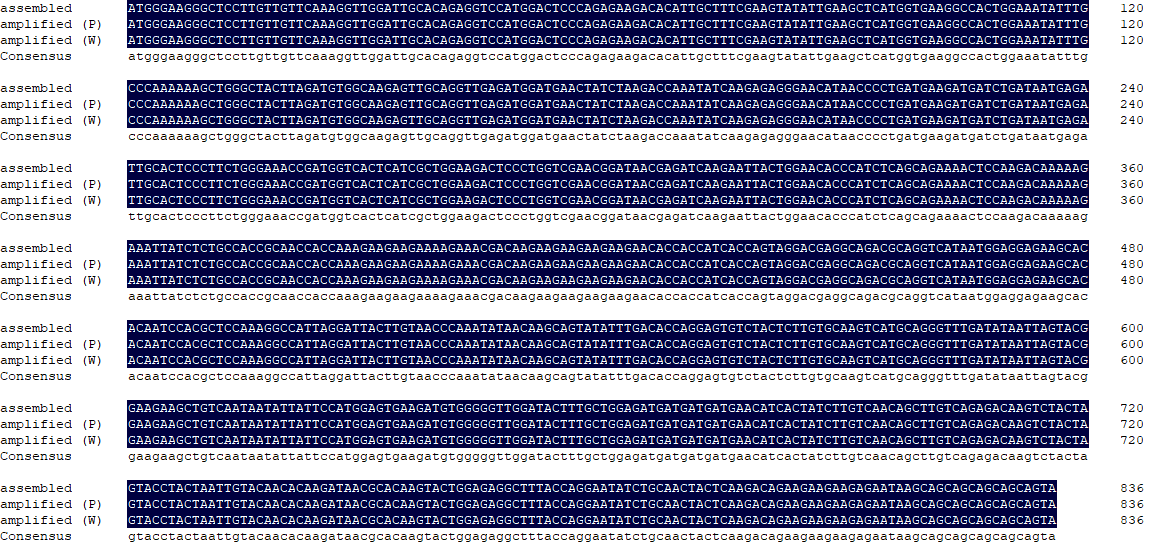
*PsMYB12*


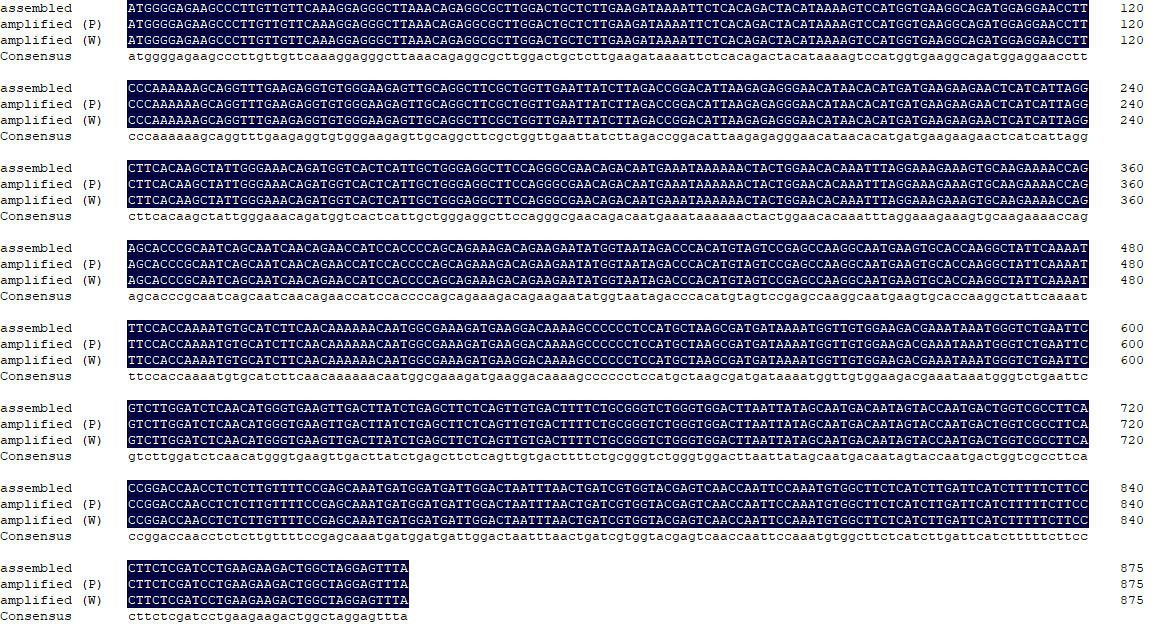
TRINITY_DN126241_c0_g1

*
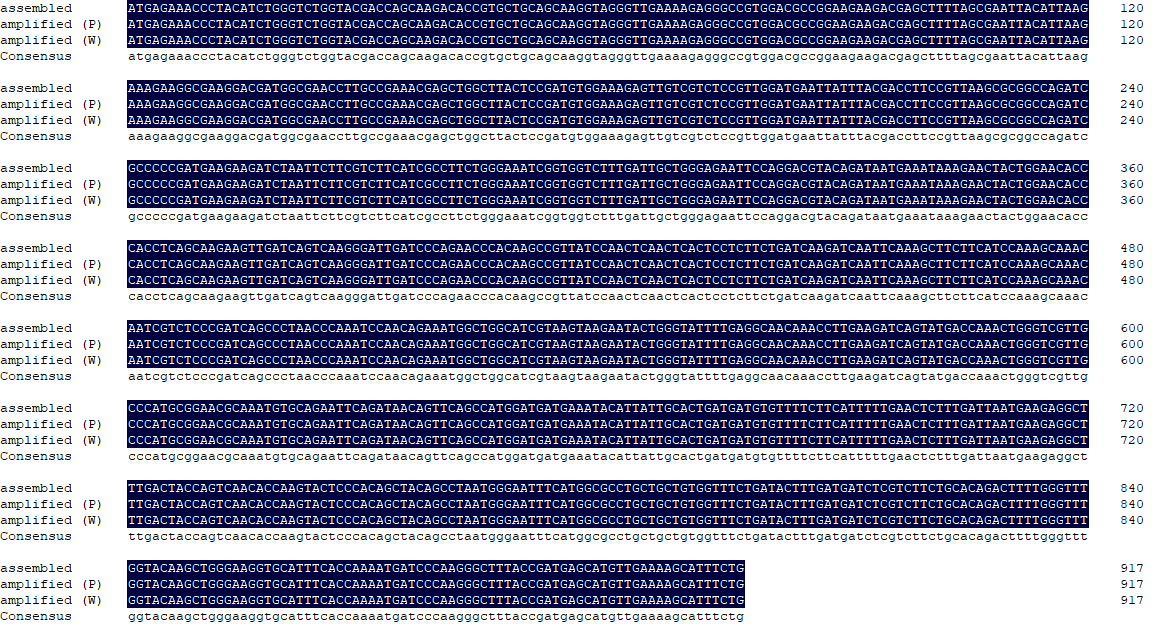
*TRINITY_DN150752_c0_g2

*
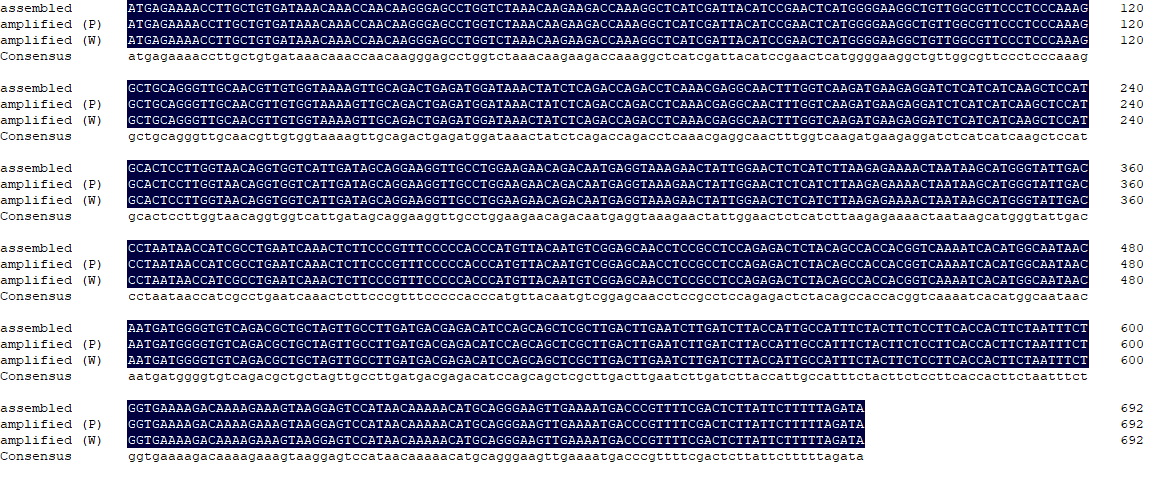
*TRINITY_DN45879_c0_g1

*
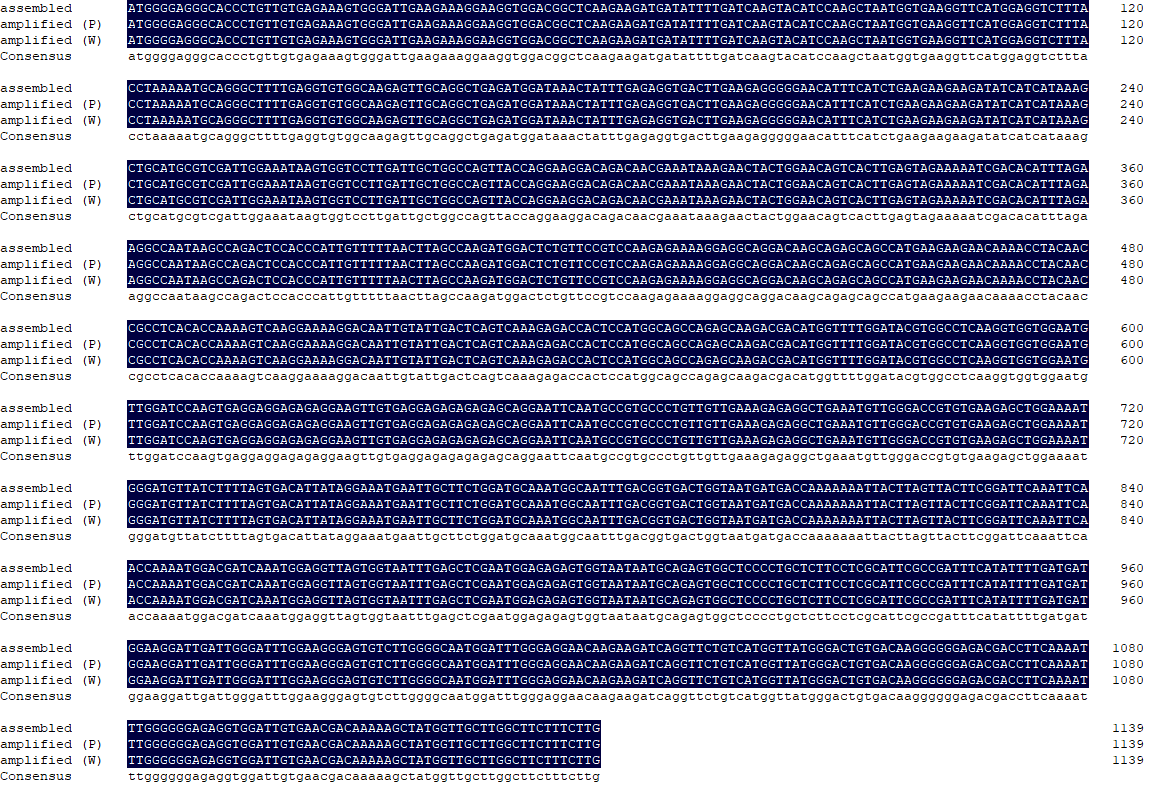
*TRINITY_DN22164_c0_g2

*
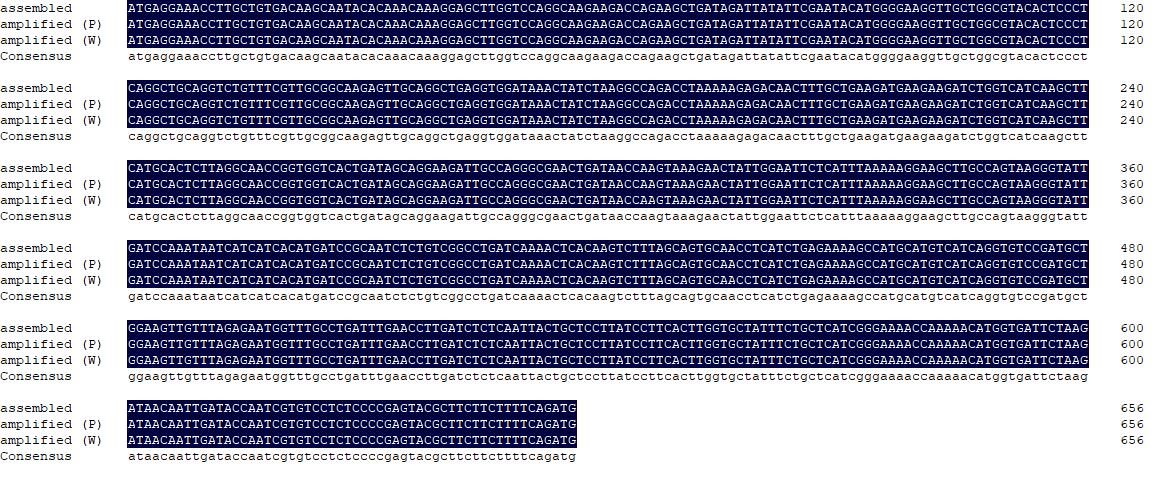
*TRINITY_DN80390_c0_g2

*
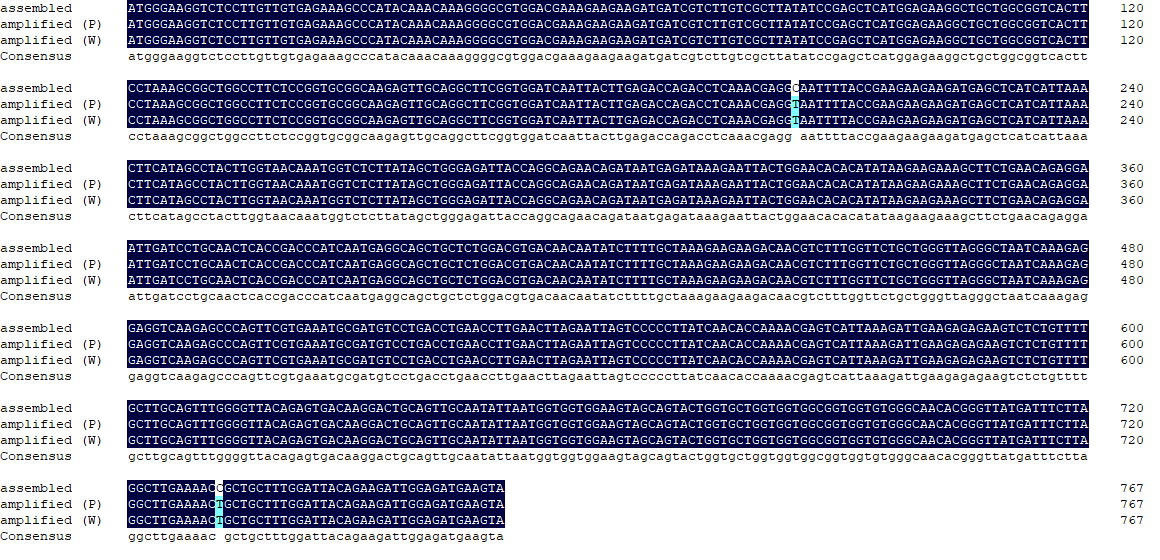
*TRINITY_DN210991_c0_g1

*
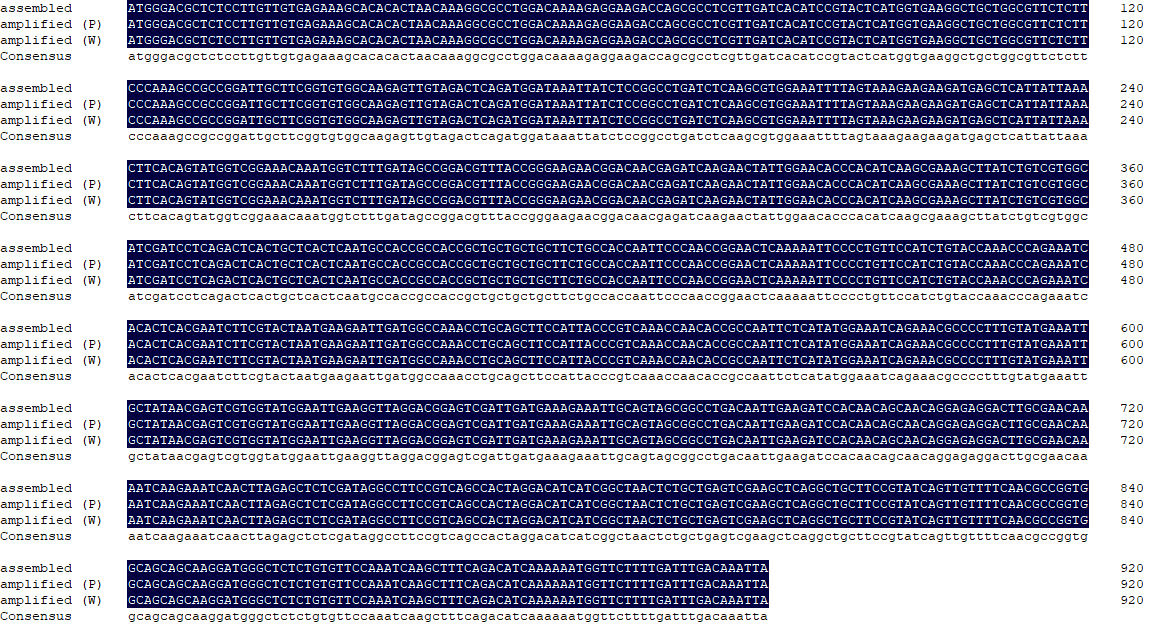
*TRINITY_DN22762_c0_g1

*
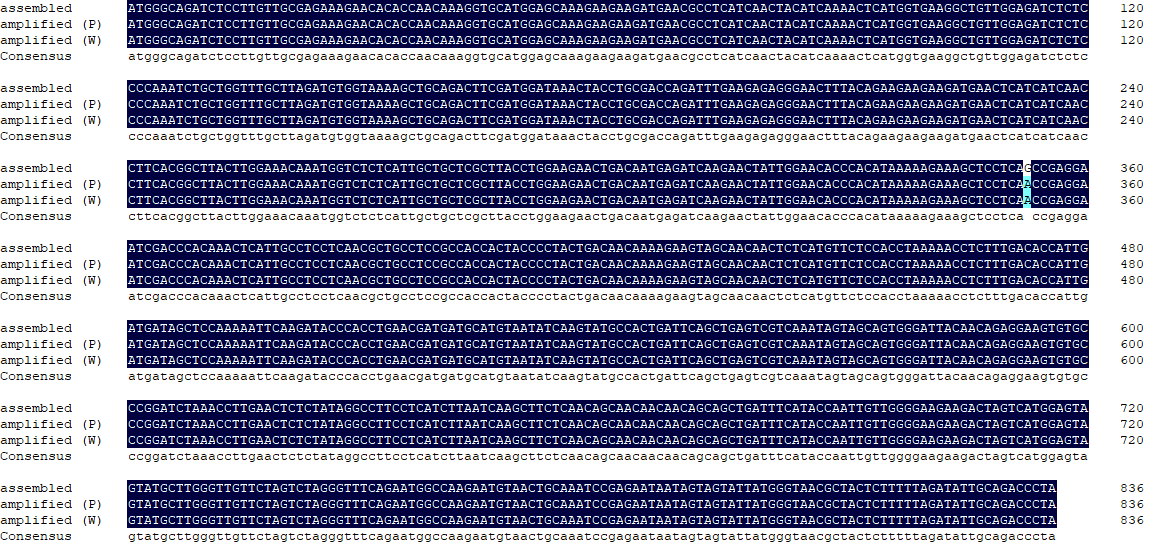
*TRINITY_DN186385_c0_g1

Fig. S5. The sequence alignment of the 12 genes encoding R2R3-MYB. Sequence assembled were got from RNA-Seq data, sequence amplified were obtained by PCR, P or W represents blotch or non-blotch area of petal.
