# Supplementary material for: The combination of DNA methylation and positive regulation of anthocyanin biosynthesis by MYB and bHLH transcription factors contributes to the petal blotch formation in Xibei tree peony
Source: Hortic Res. 2023 May 19;10(7):uhad100. doi: 10.1093/hr/uhad100 (PMC10327543; doi:10.1093/hr/uhad100)
Supplement: Web_Material_uhad100 [file web_material_uhad100.zip › Supplementary-FigureS1-S4,S6-S21.docx]

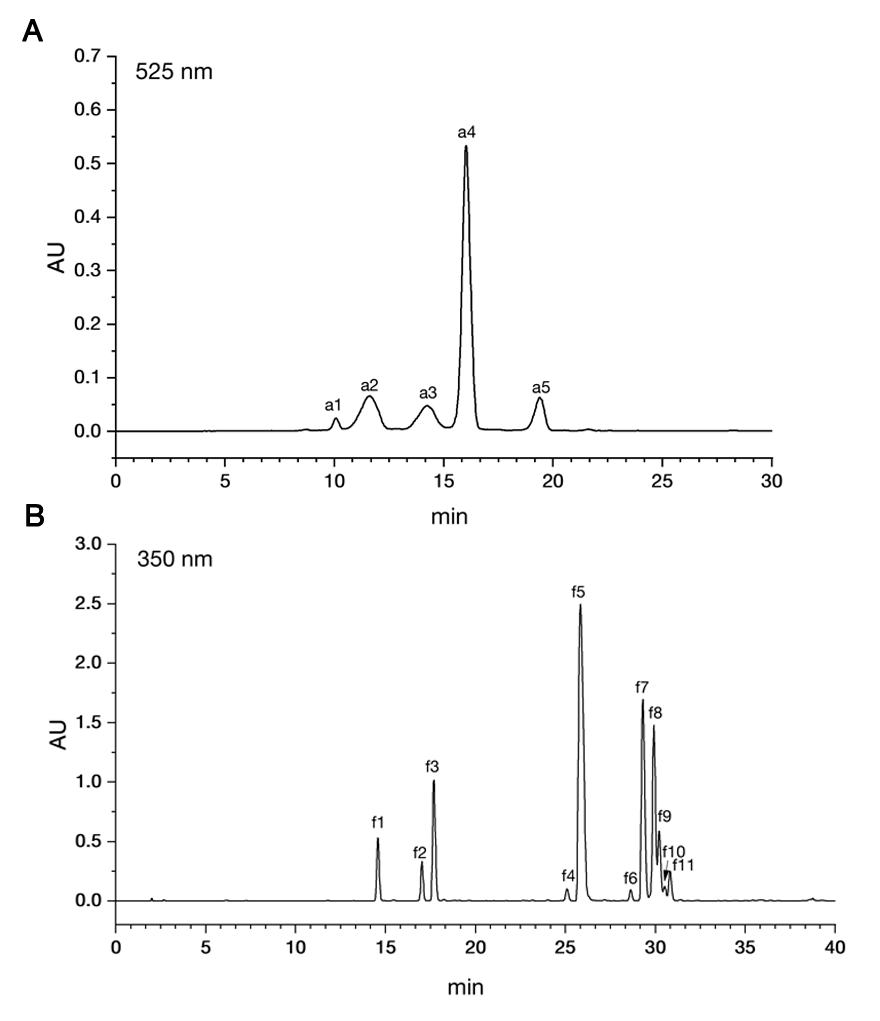
Fig. S1. HPLC-DAD chromatogram of flavonoids in *P. rockii* ‘Shu Sheng Peng Mo’ flowers. Data were recorded at 525 nm (A, for anthocyanins) and 350 nm (B, for flavones and flavonols). Peak numbers refer to the supplementary Table 1, data of S5 sample was used as a guidance.


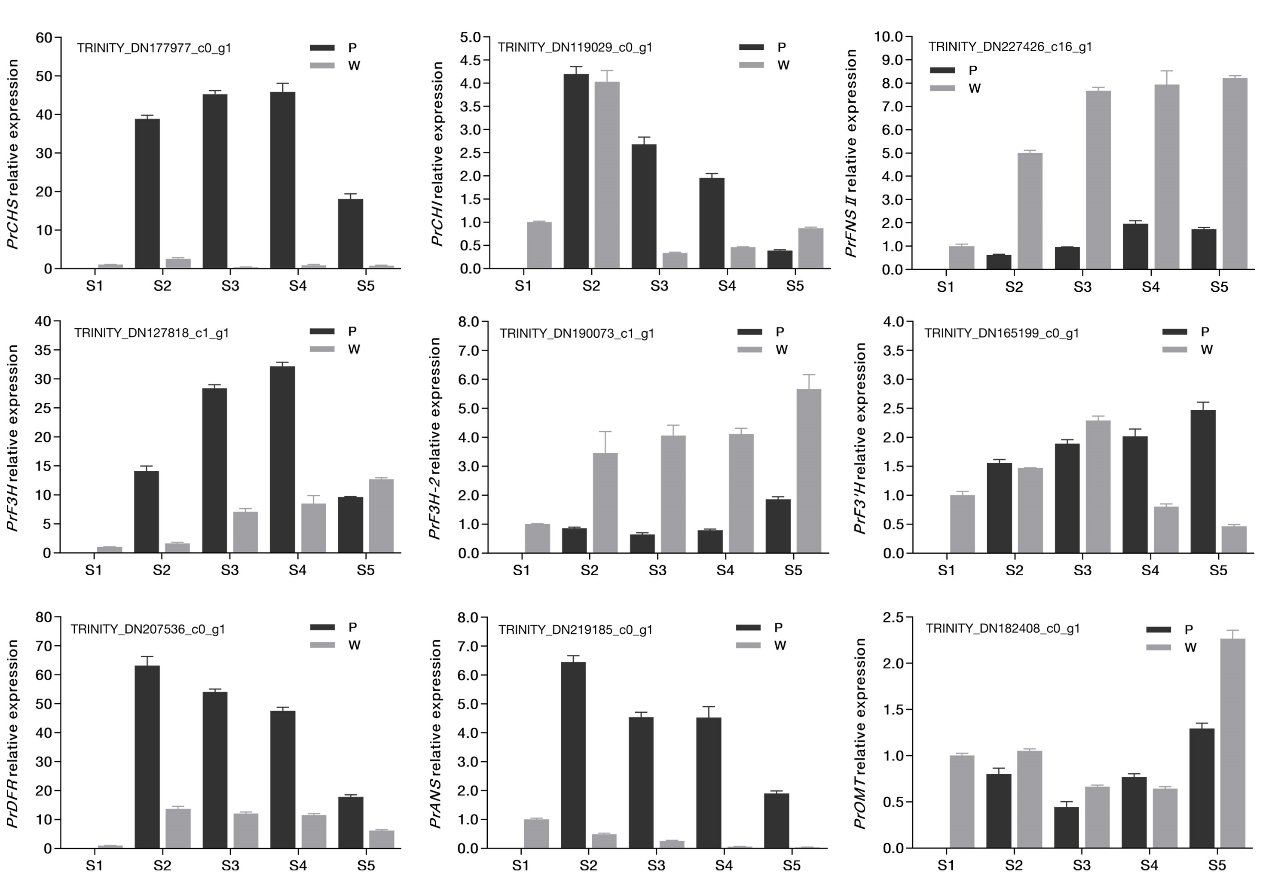
Fig. S2. qRT-PCR analysis of the DEGs. Results were consistent with the RNA-Seq data, P or W represents purple or white area of petal.

Fig. S3.
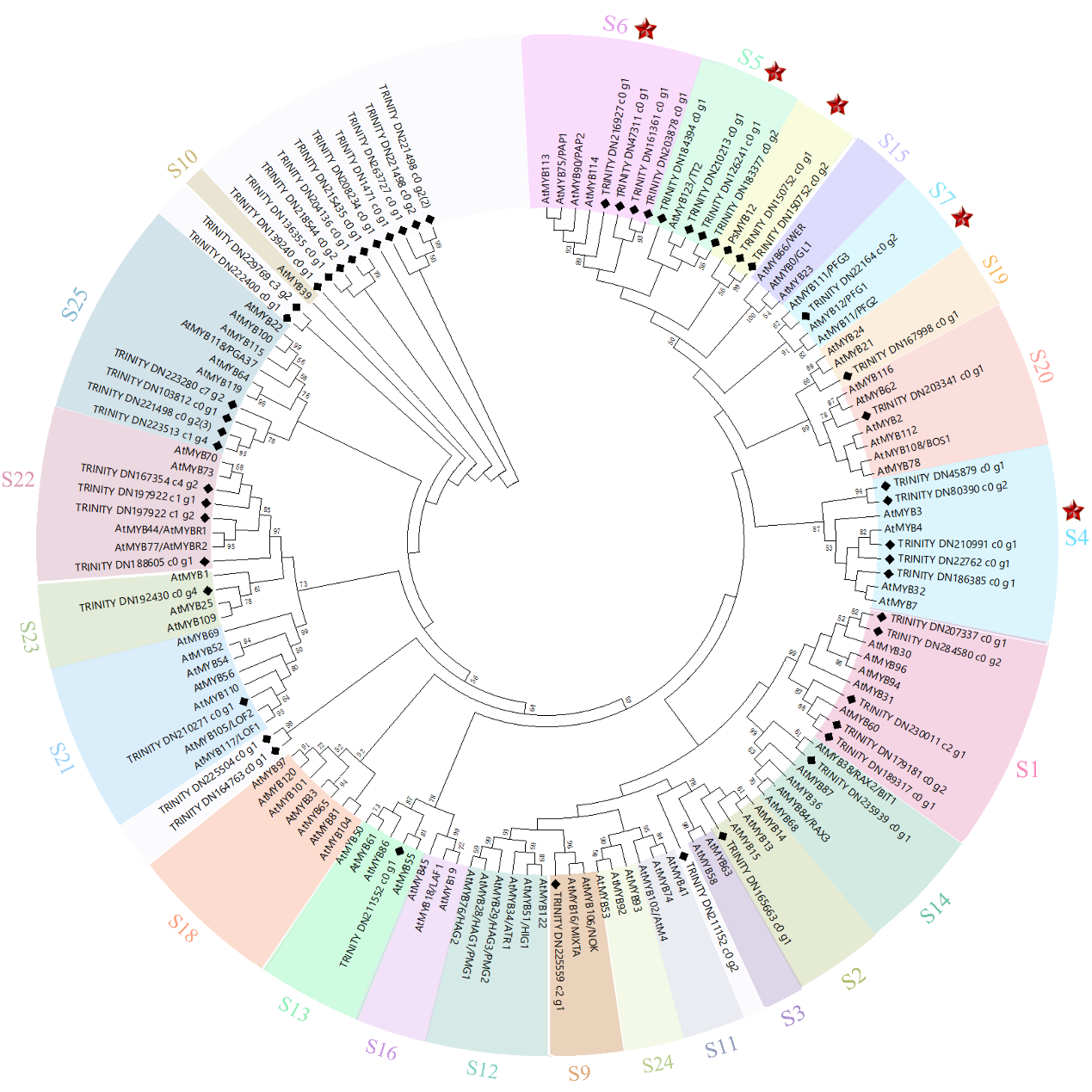
 A phylogenetic tree of 140 MYB proteins (87 from Arabidopsis, 53 from *P. rockii* ‘Shu Sheng Peng Mo’). The phylogenetic tree was constructed by the neighbor-joining method with 1000 bootstrap replications, 23 subgroups were marked with different colors. SG4，SG5，SG6，SG7 and the distinct subgroup that containing PsMYB12 were marked with red stars. Sequences of Arabidopsis were retrieved from the NCBI database (https://www.ncbi.nlm.nih.gov/https://www.ncbi.nlm.nih.gov/), the GenBank accession numbers of the MYB proteins are as follows: AtMYB30 (Q9SCU7.1), AtMYB31 (NP_177603.1), AtMYB60 (Q8GYP5.1), AtMYB94 (Q9SN78.1), AtMYB96 (Q24JK1.1), AtMYB13 (Q9LNC9.1), AtMYB14 (Q9SJX8.1), AtMYB15 (Q9LTC4.1), AtMYB58 (Q9SA47.1), AtMYB63 (Q6R0A6.1), AtMYB3 (Q9S9K9.1), AtMYB4 (Q9SZP1.1), AtMYB7 (Q42379.1), AtMYB32 (O49608.1), AtMYB123 ( Q9FJA2.1), AtMYB113 (Q9FNV9.1), AtMYB114 (Q9FNV8.1), AtMYB75 (Q9FE25.1), AtMYB90 (Q9ZTC3.1), AtMYB11 (Q9LZK4.1), AtMYB12 (O22264.1), AtMYB111 (Q9FJ07.1), AtMYB16 (Q9LXF1.1), AtMYB106 (Q9LE63.1), AtMYB39 (Q8GWP0.1), AtMYB41 (Q9M0J5.1), AtMYB74 (Q9M0Y5.1), AtMYB102 (Q9LDR8.1), AtMYB28 (Q9SPG2.1), AtMYB29 (Q9FLR1.1), AtMYB24 (Q9SPG9.1), AtMYB51 (O49782.2)， AtMYB76 (Q9SPG5.1), AtMYB122 (Q9C9C8.1), AtMYB50 (NP_176068.1), AtMYB55 (NP_001031571.1), AtMYB61 (Q8VZQ2.1), AtMYB86 (Q8LPH6.1), AtMYB36 (Q9FKL2.1), AtMYB38 (Q9SJL7.1), AtMYB68 (NP_201380.1), AtMYB84 (Q9M2Y9.1), AtMYB87 (F4JSU0.1), AtMYB0 (P27900.2), AtMYB23 (Q96276.1), AtMYB66 (Q9SEI0.1), AtMYB18 (Q9M0K4.2), AtMYB19 (NP_200039.1), AtMYB45 (NP_190461.1), AtMYB33 (Q8W1W6.1), AtMYB65 (Q9FR97.1), AtMYB81 (NP_180264.1), AtMYB97 (Q9S773.1), AtMYB101 (O80883.1), AtMYB104 (Q9SM27.3), AtMYB120 (Q94FL7.1), AtMYB21 (Q9LK95.1)，AtMYB24 (Q9SPG9.1)，AtMYB2 (BAA03534.1), AtMYB62 (Q9C9G7.1), AtMYB78 (Q9FGY3.1), AtMYB108 (Q9LDE1.1), AtMYB112 (NP_564519.1), AtMYB116 (NP_001321760.1), AtMYB52 (Q6R0C4.1), AtMYB54 (Q9FX36.1), AtMYB56 (Q6R053.1), AtMYB69 (NP_195071.1), AtMYB105 (Q9SEZ4.1), AtMYB110 (NP_566841.1), AtMYB117 (Q9LQX5.1), AtMYB44 (Q9FDW1.1), AtMYB70 (NP_179910.1), AtMYB73 (O23160.1), AtMYB77 (Q9SN12.1), AtMYB1 (Q42575.1), AtMYB25 (O04192.1), AtMYB109 (NP_191132.1), AtMYB53 (Q9FJP2.1), AtMYB92 (Q9SBF3.1), AtMYB93 (Q9S9Z2.1), AtMYB22 (NP_568582.1), AtMYB64 (Q9FY60.1), AtMYB100 (NP_180095.1), AtMYB115 (Q1PDP9.1), AtMYB118 (Q9LVW4.1), AtMYB119 (Q9FIM4.1).


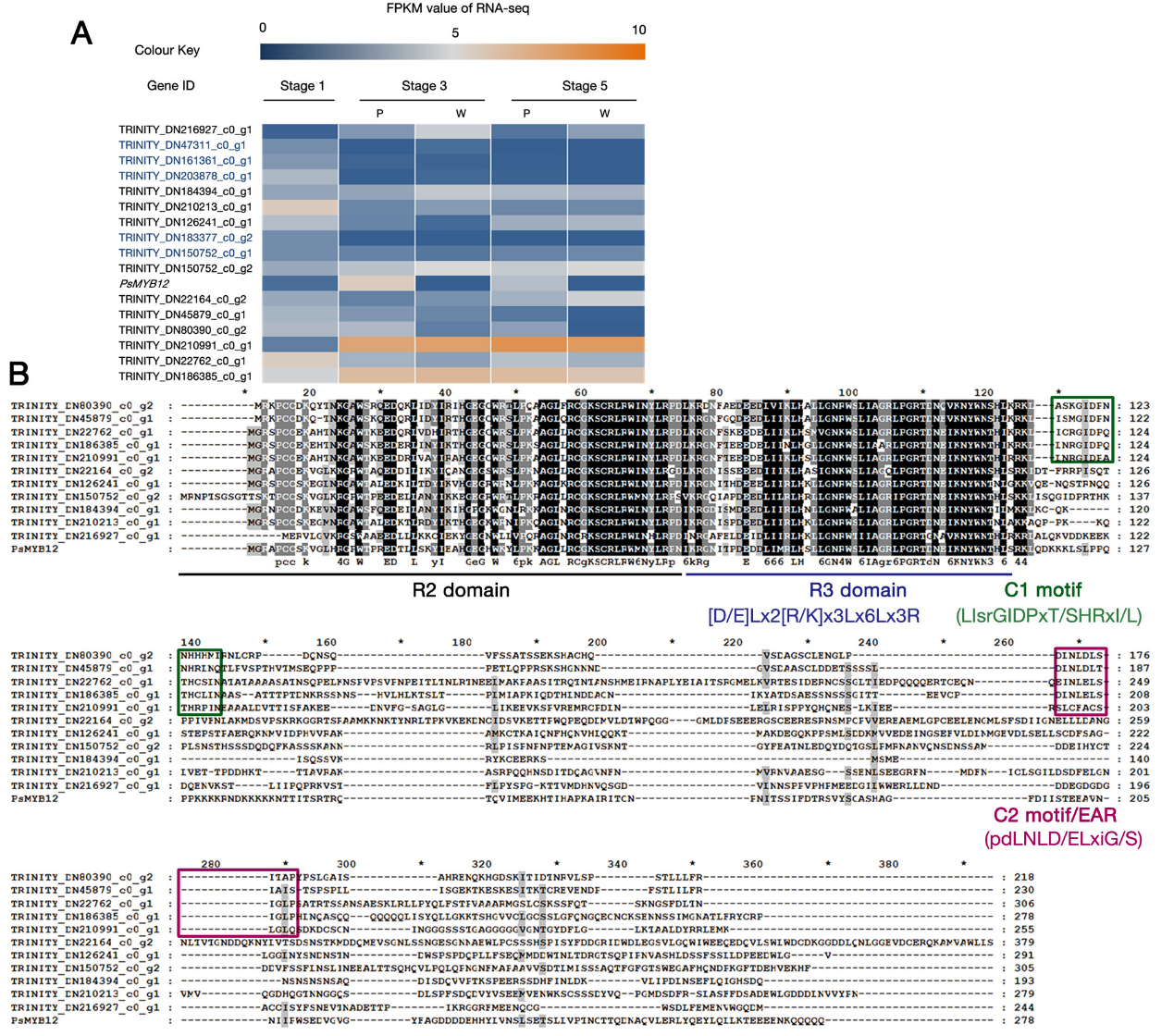
Fig. S4. The analysis of candidate *PrMYB* genes. (A) Transcription pattern of the 16 *PrMYBs* and *PsMYB12* (a highly homologous gene in *P. rockii* ‘Shu Sheng Peng Mo’) that presumed to be involved in flavonoid biosynthesis (based on FPKM values). Orange or blue indicates high or low transcription level, respectively. The members with low abundance were marked in blue font. (B) Multiple sequence alignment of the 12 full-length genes with normally transcriptions. The highly conserved R2 and R3 domain were marked with black and blue underlines. The bHLH-interacting motif ([D/E] L_x2_[R/K]_x3_L_x6_L_x3_R) was in the R3 region, the C1 motif (LIsrGIDPxT/SHRxI/L) and C2 motif (pdLNLD/ELxiG/S) were framed in green and rose red box, respectively.


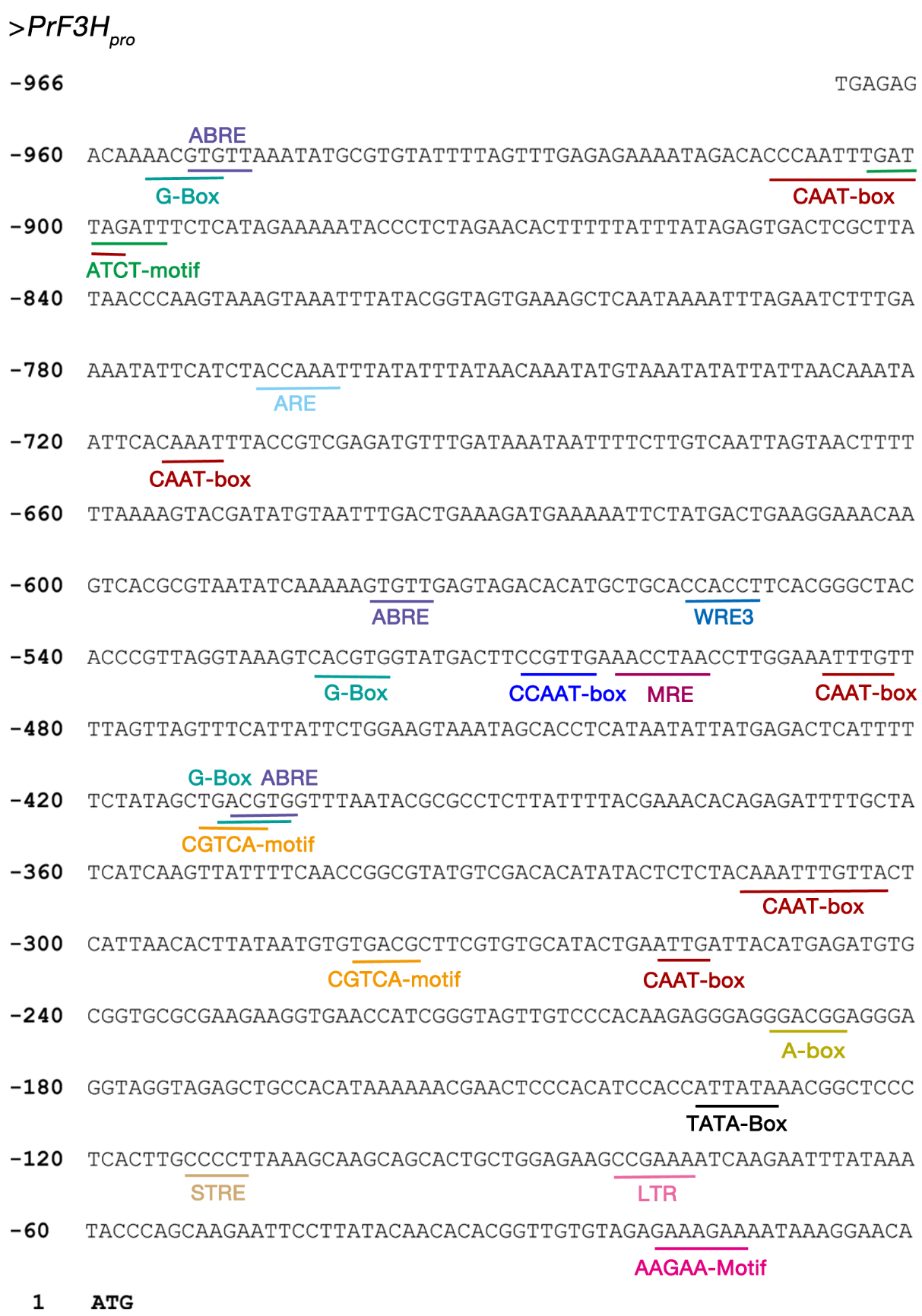
Fig. S6. Sequence of *PrF3H* promoter (from -1 to -996 bp). A of ATG marked for +1, cis-acting elements were labeled with colored underline, a MYB-binding sites (MRE) and a CCAAT-box were located in -509 to -496bp.


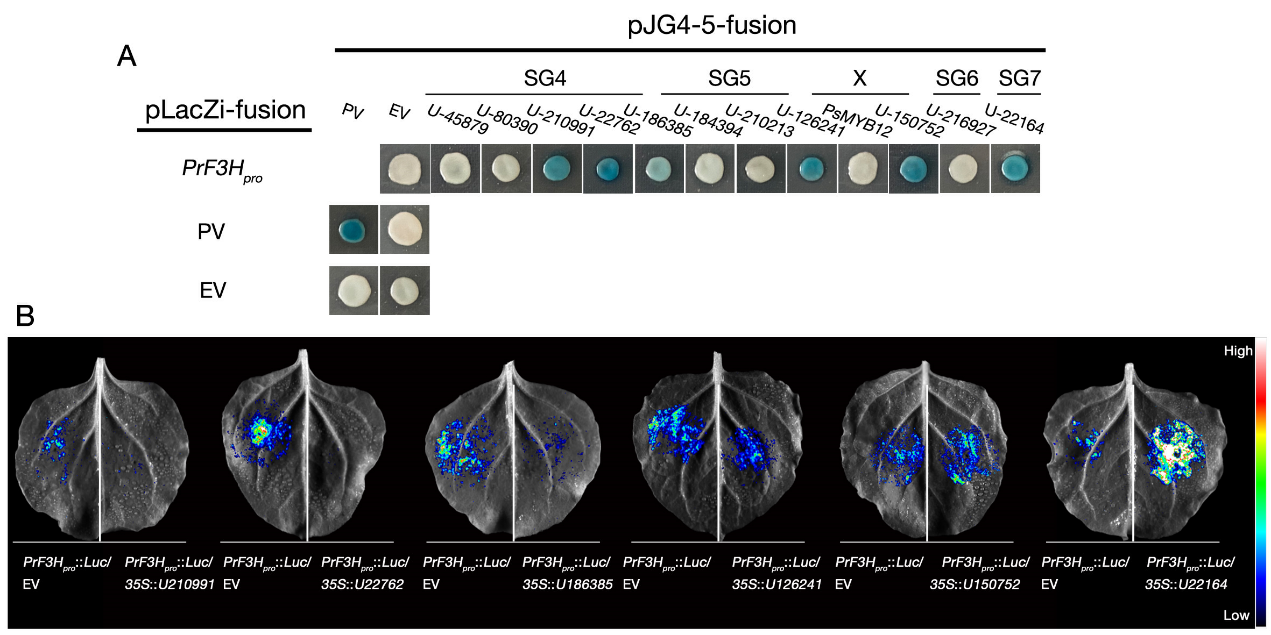
Fig. S7. Y1H assay and dual-LUC reporter assay for screening for the PrMYB transcription factors that regulate *PrF3H* expression. (A) Six candidate MYB transcription factors were obtained by Y1H assay. PrMYB transcription factors are belonging to SG4, SG5, SG6, SG7 and the distinct subgroup (represented by X). EV indicates the empty vector, PV indicates the positive vector, pLacZi-*AtLDOX_pro_* (-1702 to -1 bp) and pJG4-5-*AtPAP1* were used as positive control. Shorter codes were used to replace gene IDs in previous part of the paper, as follows: U-45879 (TRINITY_DN45879_c0_g1), U-80390 (TRINITY_DN80390_c0_g2), U-210991 (TRINITY_DN210991_c0_g1), U-22762 (TRINITY_DN22762_c0_g1), U-186385 (TRINITY_DN186385_c0_g1), U-184394 (TRINITY_DN184394_c0_g1), U-210213 (TRINITY_DN210213_c0_g1), U-126241 (TRINITY_DN126241_c0_g1), U-150752 (TRINITY_DN150752_c0_g2), U-216927 (TRINITY_DN216927_c0_g1), U-22164 (TRINITY_DN22164_c0_g2). (B) One of the six MYB proteins could activates the reporter gene, on the basis of the intensity of luciferase.


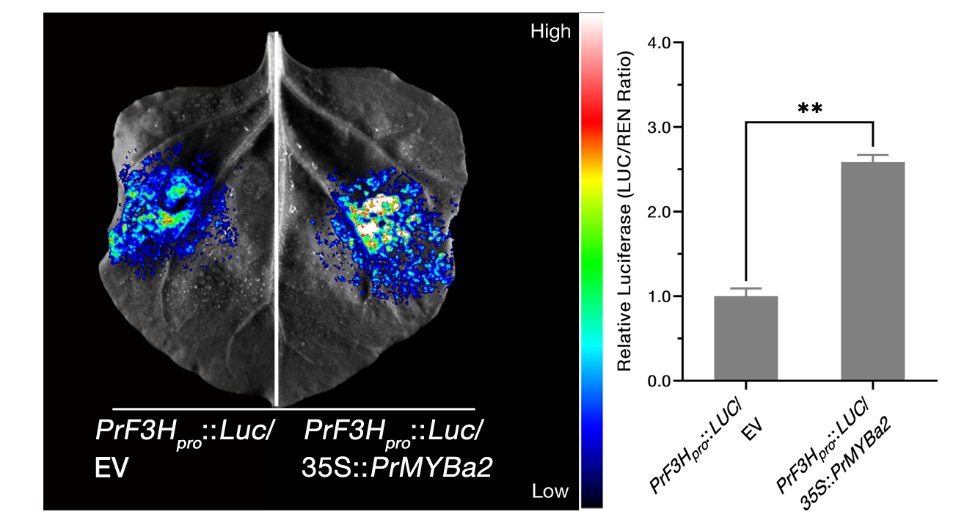
Fig. S8. Dual-LUC reporter assay confirmed that PrMYBa2 effects on *PrF3H* promoter and activates the reporter gene. Values represent means ± SD (n = 4), asterisks indicate statistically significant differences (two-sided Student’s t test; **, *P* < 0.01). EV represents the empty vector.


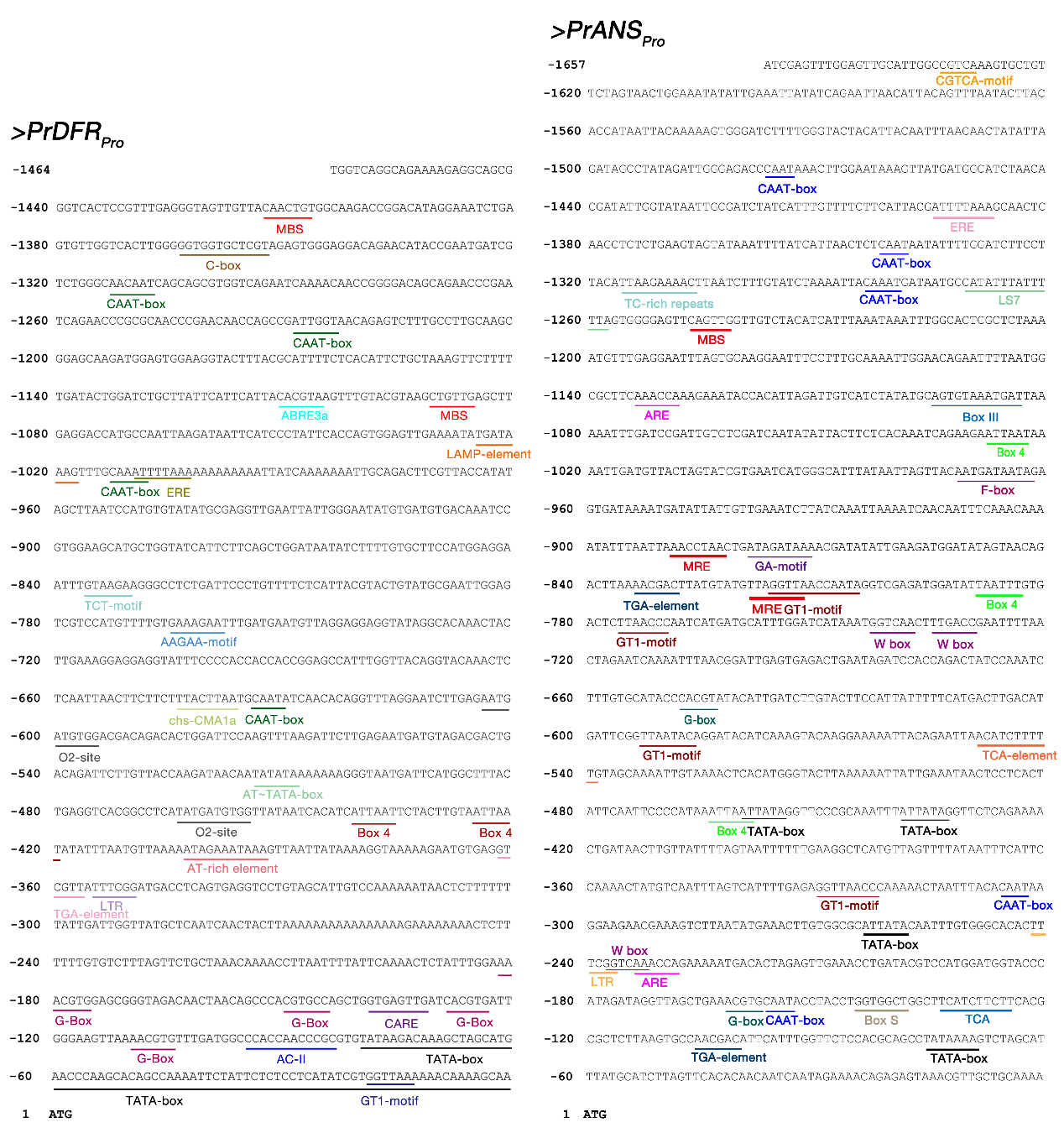
Fig. S9. Sequence of *PrDFR* promoter (from -1 to -1464 bp) and *PrANS* promoter (from -1 to -1657 bp). A of ATG was marked for +1 bp, cis-acting elements were labeled with colored underline.


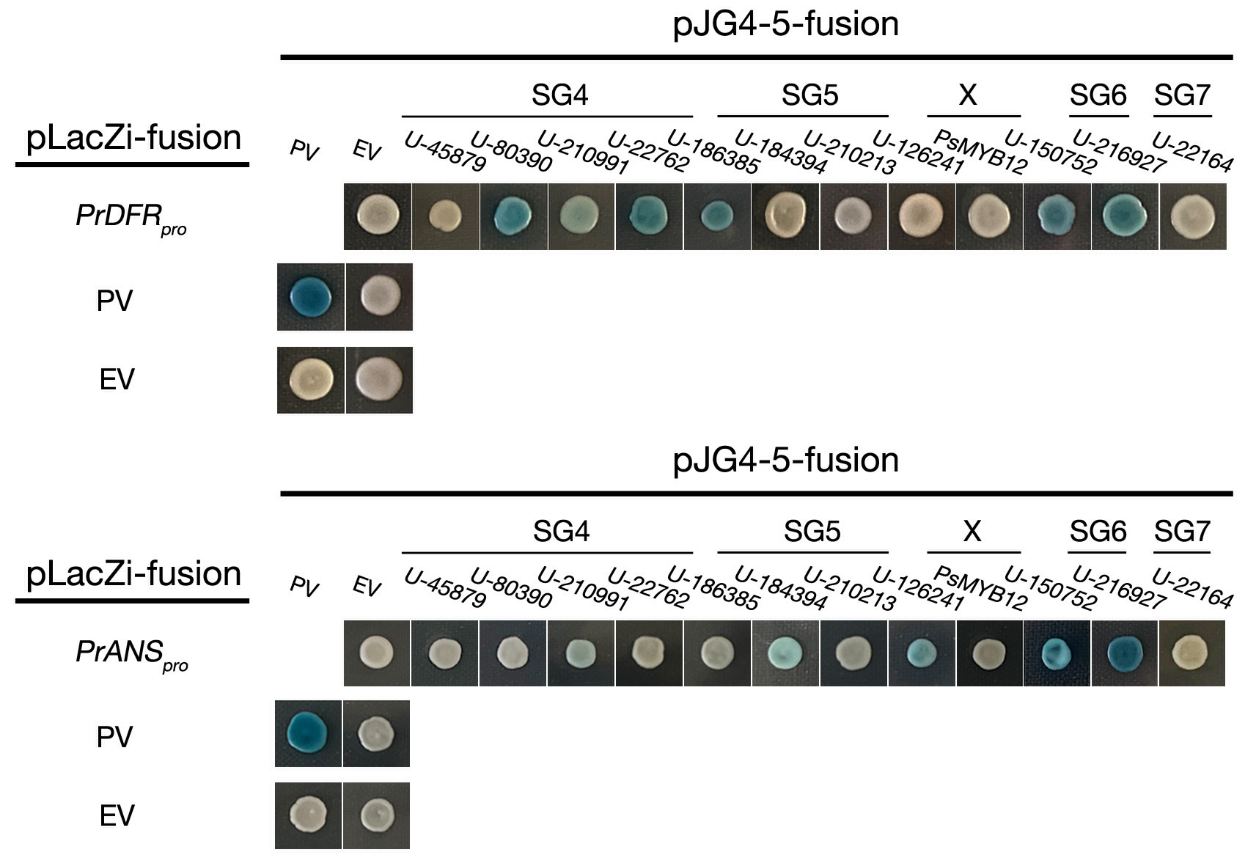
Fig. S10. Y1H assays for screening for the MYB transcription factors that bind to the promoters of *PrDFR* and *PrANS*. EV indicates the empty vector, PV indicates the positive vector, pLacZi-*AtLDOX_pro_* (-1702 to -1 bp) and pJG4-5-*AtPAP1* were used as positive control. Shorter codes were used to replace the gene IDs in previous part of the paper, as follows: U-45879 (TRINITY_DN45879_c0_g1), U-80390 (TRINITY_DN80390_c0_g2), U-210991 (TRINITY_DN210991_c0_g1), U-22762 (TRINITY_DN22762_c0_g1), U-186385 (TRINITY_DN186385_c0_g1), U-184394 (TRINITY_DN184394_c0_g1), U-210213 (TRINITY_DN210213_c0_g1), U-126241 (TRINITY_DN126241_c0_g1), U-150752 (TRINITY_DN150752_c0_g2), U-216927 (TRINITY_DN216927_c0_g1), U-22164 (TRINITY_DN22164_c0_g2).


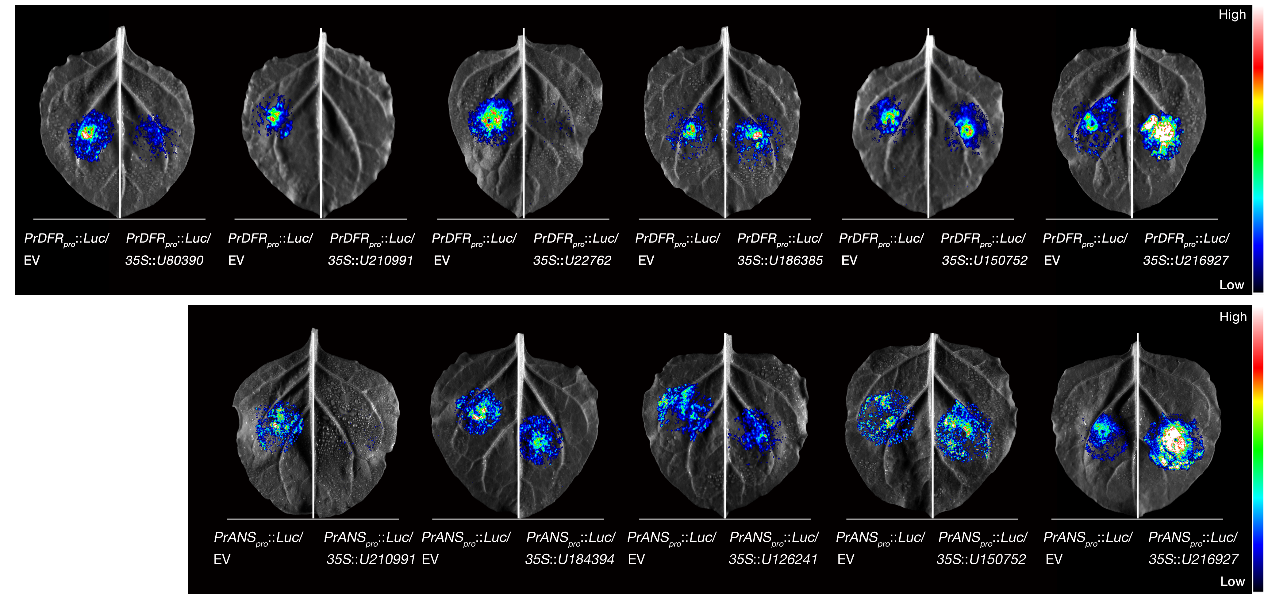
Fig. S11. Dual-LUC reporter assays for further verification of the activators from candidate PrMYBs screened by Y1H assay.



Fig. S12. Phylogenetic analysis of 196 PrbHLH proteins (143 from Arabidopsis, 53 from *P. rockii* ‘Shu Sheng Peng Mo’). The phylogenetic tree was constructed by the neighbor-joining method with 1000 bootstrap replications, 31 subgroups were marked with different colors. PrbHLHs were marked with black diamond, the fifth subgroup (Ⅲf) were marked with red star. Sequences of Arabidopsis were retrieved from the NCBI database (https://www.ncbi.nlm.nih.gov/https://www.ncbi.nlm.nih.gov/), the GenBank accession numbers of the bHLH proteins are as follows: AtbHLH37 (Q9SND4.1), AtbHLH88 (Q9FHA7.1), AtbHLH43 (Q9LXD8.1), AtbHLH40 (O81313.3), AtbHLH87 (Q8S3D2.1), AtbHLH52 (Q9SA82.1), AtbHLH53 (Q84RD0.1), AtbHLH83 (Q9C707.1), AtbHLH86 (Q9FJ00.2), AtbHLH84 (Q7XHI9.1), AtbHLH85 (Q84WK0.1), AtbHLH54 (Q8LEG1.1), AtbHLH139 (Q3E7L7.1), AtbHLH80 (Q9C8P8.1), AtbHLH81 (Q9M0R0.1), AtbHLH129 (Q9ZW81.2), AtbHLH128 (Q8H102.1), AtbHLH122 (Q9C690.1), AtbHLH130 (Q66GR3.1), AtbHLH7 (Q93Y00.1), AtbHLH59 (O22768.2), AtbHLH82 (Q9LSQ3.1), AtbHLH69 (Q8S3D5.2), AtbHLH66 (Q9ZUG9.1), AtbHLH44 (Q8GZ13.1), AtbHLH50 (Q8GWK7.1), AtbHLH75 (A4D998.1), AtbHLH31 (Q0JXE7.1), AtbHLH79 (Q9LV17.1), AtbHLH48 (Q8VZ02.1), AtbHLH60 (Q3EAI1.1), AtbHLH137 (Q93W88.1), AtbHLH58 (Q93VJ4.1), AtbHLH64 (Q9ZPW3.3), AtbHLH49 (Q9CAA9.1), AtbHLH76 (Q9C670.1), AtbHLH77 (Q9LK48.1), AtbHLH78 (Q9FJL4.1), AtbHLH62 (Q9SRT2.1)，AtbHLH109 (Q5XVH0.1), AtbHLH124 (Q8L5W8.1), AtbHLH132 (Q8L5W7.1), AtbHLH26 (Q9FE22.1), AtbHLH127 (Q7XHI7.1), AtbHLH56 (Q9SVU7.2), AtbHLH23 (Q9SVU6.1), AtbHLH119 (Q8GT73.2), AtbHLH16 (Q8GZ38.1), AtbHLH72 (Q570R7.2), AtbHLH24 (Q9FUA4.1), AtbHLH73 (Q9FHA2.1), AtbHLH8 (O80536.1), AtbHLH15 (Q8GZM7.1), AtbHLH9 (Q8W2F3.1), AtbHLH65 (Q84LH8.1), AtbHLH141(Q9FMB6.1), AtbHLH46 (Q9LEZ3.2), AtbHLH102 (Q9CAA4.1), AtbHLH68 (Q8S3D1.2), AtbHLH110 (Q9SFZ3.2), AtbHLH113 (Q9LT67.1), AtbHLH123 (Q8GXT3.1), AtbHLH112 (Q94JL3.1), AtbHLH103 (Q8VZ22.1), AtbHLH114 (Q9M0X8.2), AtbHLH142 (Q9FMF4.1), AtbHLH143 (Q9FY69.1), AtbHLH144 (Q9ASX9.1), AtbHLH156 (Q9XIN0.1), AtbHLH165 (Q9SJH0.1), AtbHLH166 (Q9LXR7.1), AtbHLH158 (Q9SKX1.1), AtbHLH149 (O80482.1), AtbHLH148 (Q9C8Z9.1), AtbHLH147 (Q9LSN7.1), AtbHLH94 (Q9SK91.2), AtbHLH96 (Q9C7T4.1), AtbHLH71 (Q56XR0.1), AtbHLH67 (Q700E4.1), AtbHLH57 (Q9M128.1), AtbHLH70 (O81037.1), AtbHLH97 (Q56YJ8.1), AtbHLH45 (Q9M8K6.1), AtbHLH98 (Q700C7.1), AtbHLH99 (Q9FKQ6.1), AtbHLH1 (Q9FN69.1), AtbHLH2 (Q9CAD0.1), AtbHLH42 (Q9FT81.2), AtbHLH12 (Q8W2F1.1), AtbHLH10 (Q84TK1.1), AtbHLH89 (Q9LND0.1), AtbHLH91 (Q8GX46.1), AtbHLH20 (Q8S3F1.1), AtbHLH19 (Q1PF16.1), AtbHLH18 (Q1PF17.1), AtbHLH25 (Q9T072.2), AtbHLH28 (Q9LUK7.1)， AtbHLH14 (O23090.1), AtbHLH6 (Q39204.2), AtbHLH4 (O49687.1), AtbHLH5 (Q9FIP9.1), AtbHLH13 (Q9LNJ5.1), AtbHLH17 (Q9ZPY8.2), AtbHLH3 (O23487.1), AtbHLH27 (Q700E3.1), AtbHLH35 (Q2HIV9.1), AtbHLH21 (Q9ZVX2.2), AtbHLH22 (O81900.1), AtbHLH61 (Q9LXA9.1), AtbHLH93 (Q9LSL1.1), AtbHLH33 (Q9LPW3.1), AtbHLH116 (Q9LSE2.1), AtbHLH136 (Q9FLE9.1), AtbHLH164 (Q9LJX1.1), AtbHLH13 (Q9LNJ5.1), AtbHLH163 (Q8GW32.1), AtbHLH135 (Q9CA64.1), AtbHLH161 (F4JCN9.1), AtbHLH95 (Q9FXA3.2), AtbHLH41 (Q9LTS4.1), AtbHLH92 (Q9FIX5.1)， AtbHLH38 (Q9M1K1.1), AtbHLH39 (Q9M1K0.1), AtbHLH100 (Q9ZVB5.1), AtbHLH101 (Q9FYE6.1), AtbHLH55 (Q9LN95.1), AtbHLH125 (Q9LQ08.1), AtbHLH120 (Q9FLI0.2), AtbHLH126 (Q9STJ6.1), AtbHLH36 (Q9FLI1.1), AtbHLH118 (Q9STJ7.1)，AtbHLH51(Q9XEF0.1), AtbHLH106 (O80674.1)，AtbHLH107(Q9LET0.1), AtbHLH32 (Q9LS08.1), AtbHLH30 (Q9S7Y1.1), AtbHLH47 (Q9SN74.1), AtbHLH11 (Q8W2F2.2), AtbHLH121 (Q9LT23.1), AtbHLH105 (Q9FH37.1), AtbHLH115 (Q9C682.1), AtbHLH34 (Q9LTC7.1), AtbHLH104 (Q8L467.1).


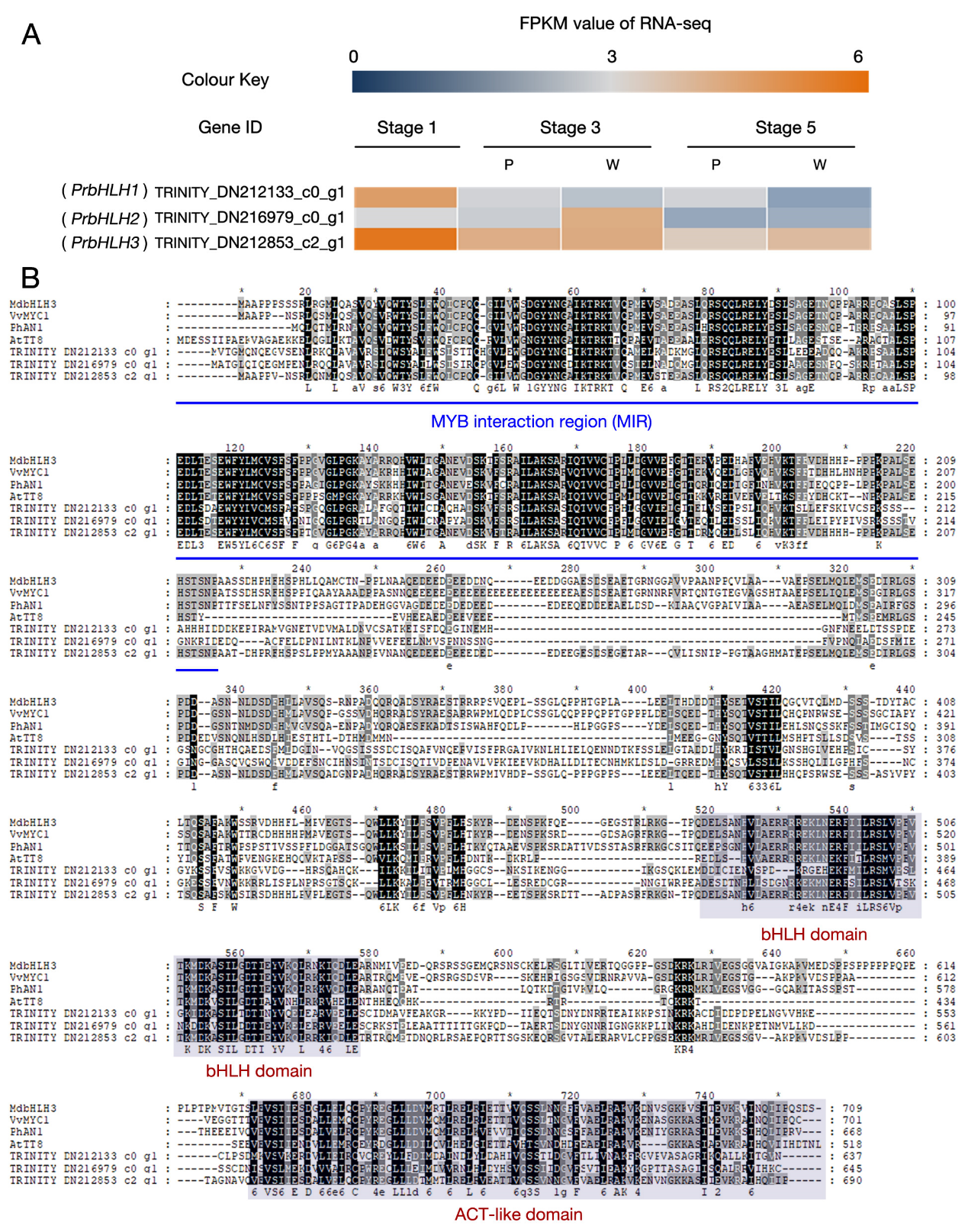
Fig. S13. The analysis of *PrbHLHs*. (A) Transcription patterns of the 3 unigenes encoding *PrbHLHs* that presumed to be involved in the flavonoid biosynthesis (Based on FPKM values). Orange or blue indicates high or low transcription level, respectively. (B) Multiple sequence alignment of the three full-length PrbHLHs and four well known anthocyanin regulators from other species (MdbHLH3, VvMYC1, PhAN1, AtTT8). The MYB interaction region was marked with blue underline, conserved bHLH domain and ACT-like domain were covered by blue boxes.

Fig. S14. Y1H assay and dual-LUC reporter assay for verification of the PrbHLH transcription factors. PrbHLH1-3 were belonged to the fifth subgroup (Ⅲf). (A) PrbHLH1-3 showed unable to bind to the promoters of neither *PrF3H*, *PrDFR* nor
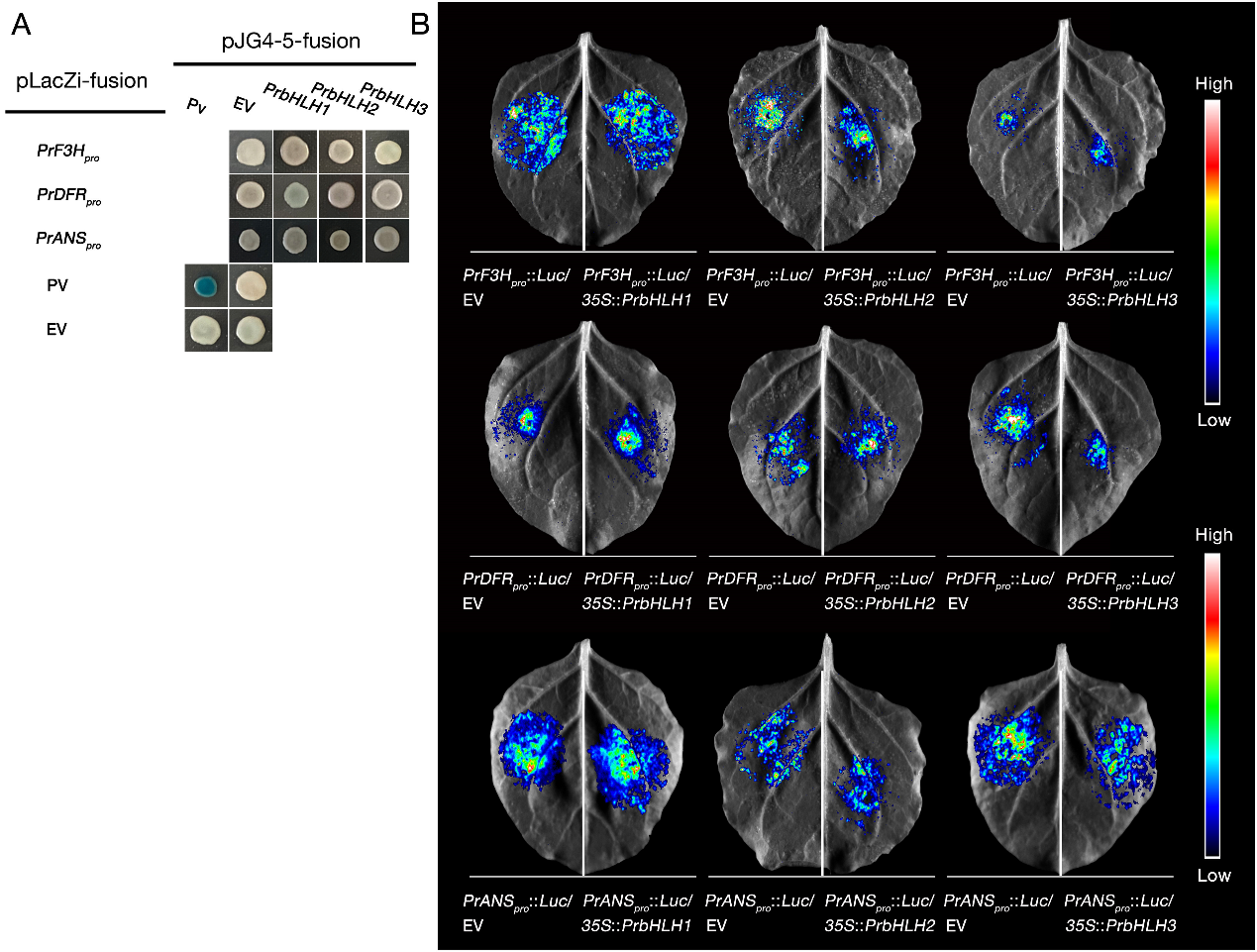
*PrANS*. EV indicates the empty vector, PV indicates the positive vector, pLacZi-*AtLDOX_pro_* (-1702 to -1 bp) and pJG4-5-*AtPAP1* were used as positive control. (B) Images of *N. benthamiana* leaves showed that the reporter gene had not been strongly activated by PrbHLH1-3.



Fig. S15. Y2H assays for verifying the interaction between PrMYBs and PrbHLH1-3. (A) *PrMYBa1* was cloned into vector pGBK-T7 while *PrbHLH1-3* were cloned into vector pGAD-T7. (B) *PrMYBa2* was cloned into vector pGBK-T7, *PrbHLH1-3* were cloned into vector pGAD-T7. (C) *PrbHLH1-3* were cloned into vector pGBK-T7 while *PrMYBa1* was cloned into vector pGAD-T7, results showed that PrMYBa1 do not interact with any of PrbHLH1-3 in yeast cells. (D) *PrbHLH1-3* were cloned into vector pGBK-T7 while *PrMYBa2* was cloned into vector pGAD-T7, results showed that PrMYBa2 do not interact with any of PrbHLH1-3 in yeast. (E) *PrbHLH1-3* were cloned into vector pGBK-T7, *PrMYBa3* was cloned into vector pGAD-T7, results showed that PrMYBa3 interact with both PrbHLH1 and PrbHLH2 in yeast cells. Positive control was pGBKT7-53+pGADT7-T, negative control was pGBKT7-lam+pGADT7-T. AD means empty vector of pGADT7, DDO means SD medium without Trp and Leu. QDO/X means SD medium without Trp, Leu, His, Ade, supplemented with X-*α*-gal. QDO/X/3-AT means SD medium without Trp, Leu, His, Ade, supplemented with X-*α*-gal and a certain concentration of 3-AT. Transformed yeast cells were dotted as 10^–1^ dilution on the selective media.


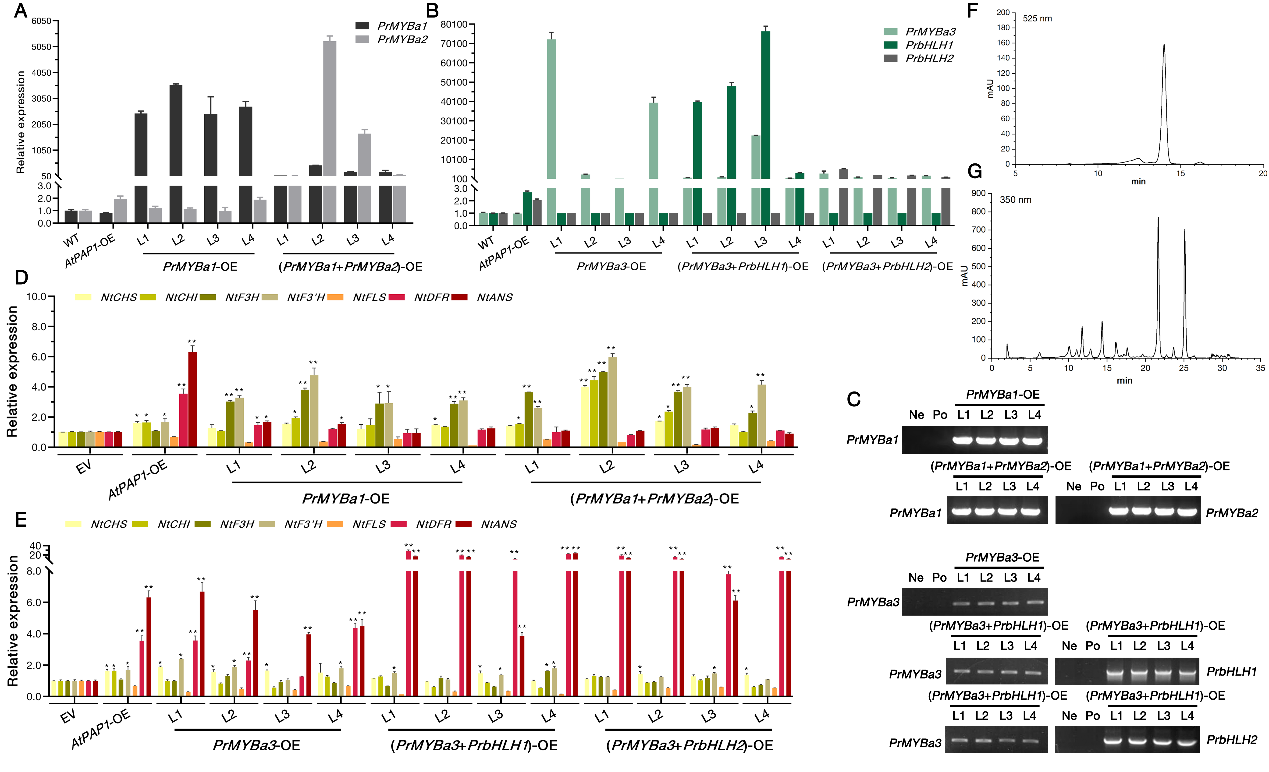
Fig. S16. Analysis of transgenic tobacco plants. (A, B) Transcription levels of *PrMYBa1*, *PrMYBa2*, *PrMYBa3*, *PrbHLH1*, *PrbHLH2* in transgenic tobacco lines and control lines. (C) The PCR amplification of the transgenes in transgenic tobacco plants. (D, E) qRT-PCR analysis of corresponding anthocyanin structure genes in transgenic tobacco lines. Values represent means ± SD (n = 3), asterisks indicate statistically significant differences (two-sided Student’s t test; *, *P* <0.05, **, *P* < 0.01). (F, G) The HPLC-DAD chromatogram of hydrolyzed extract of tobacco flowers (corollas). Detection wavelength were 525nm (for anthocyanins) and 350nm (for flavones and flavonols). Only one definite anthocyanin was identified in tobacco flowers: cyanidin 3-*O*- rutinoside (Cy3R). Flavones and flavonols were not identified.


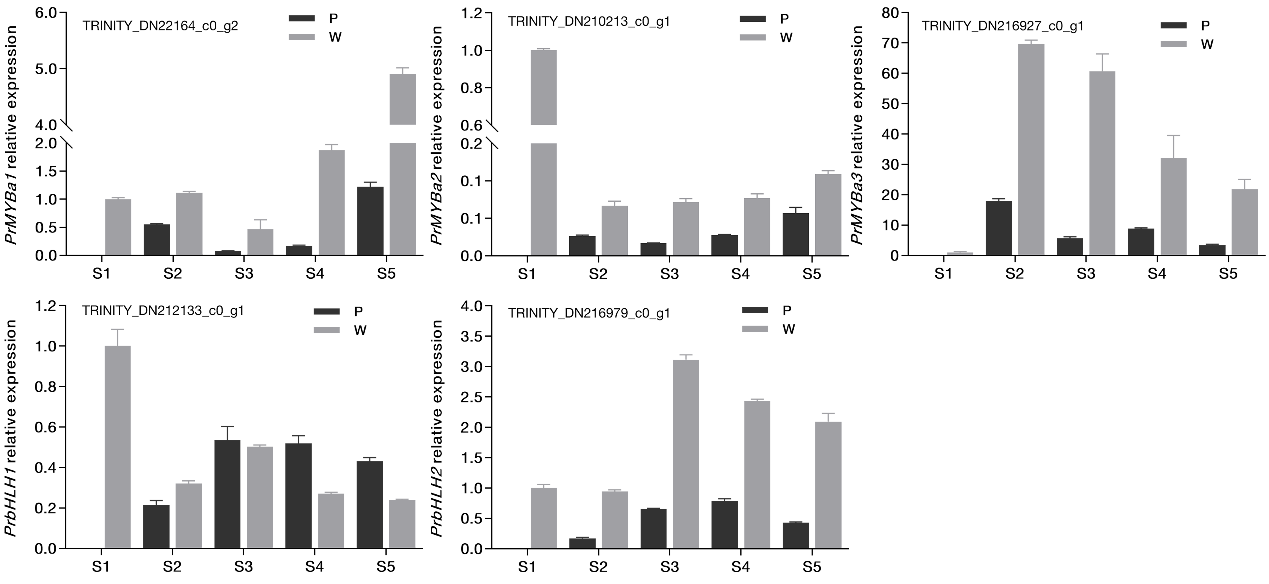
Fig. S17. The spatio-temporal expressions of *PrMYBa1*, *PrMYBa2*, *PrMYBa3*, *PrbHLH1*, *PrbHLH2*. P or W represents purple or white area of petal, respectively.


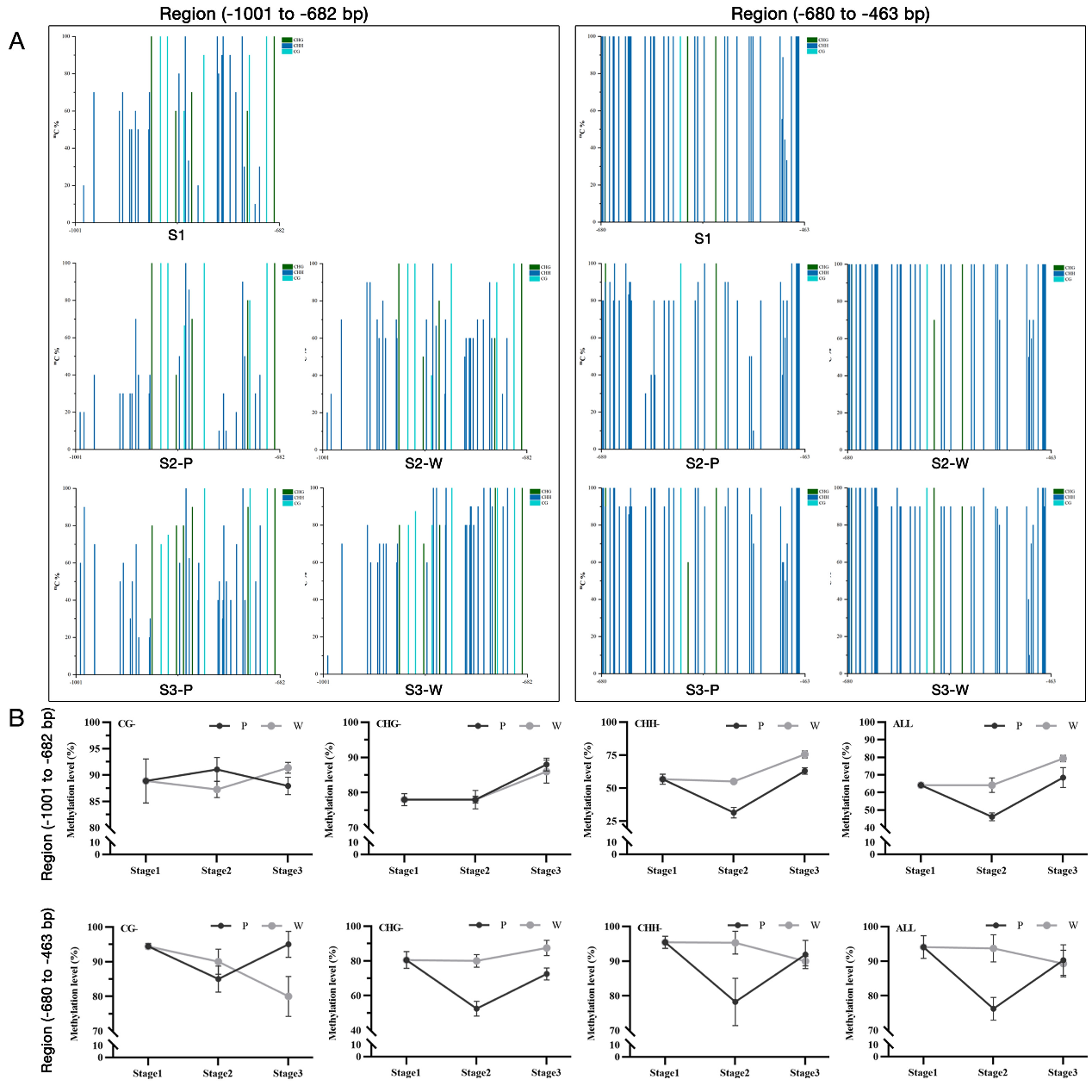
Fig. S18. The dynamics of DNA methylation modification of *PrANS* promoter during flower development. (A) The CG-type, CHG-type and CHH-type methylation levels of -1001 to -682 bp and -680 to -463 bp regions. S1-S3 means stage1 to stage3. (B) The dynamics of CG-type, CHG-type, CHH-type and total methylation levels of the two regions mentioned above. P or W represents purple or white areas of petal.

Fig. S19.

 Dual-LUC reporter assay indicated that PsMYB12 has no effect on the promoters of *PrF3H*, *PrDFR* or *PrANS.* (A) Images of *N. benthamiana* leaves showed that the reporter gene had not been activated by PsMYB12. (B) Relative luciferase activities by co-expressed of *PrF3H_pro_*::*LUC+*EV, *PrF3H_pro_*::*LUC+35S*::*PsMYB12*, and *PrDFR_pro_*::*LUC+*EV, *PrDFR_pro_*::*LUC+35S*:: *PsMYB12* and *PrANS_pro_*::*LUC+*EV, *PrANS_pro_*::*LUC+35S*:: *PsMYB12*. Values represent means ± SD (n = 4), ns indicate statistically not differences (two-sided Student’s t test).

Fig. S20.
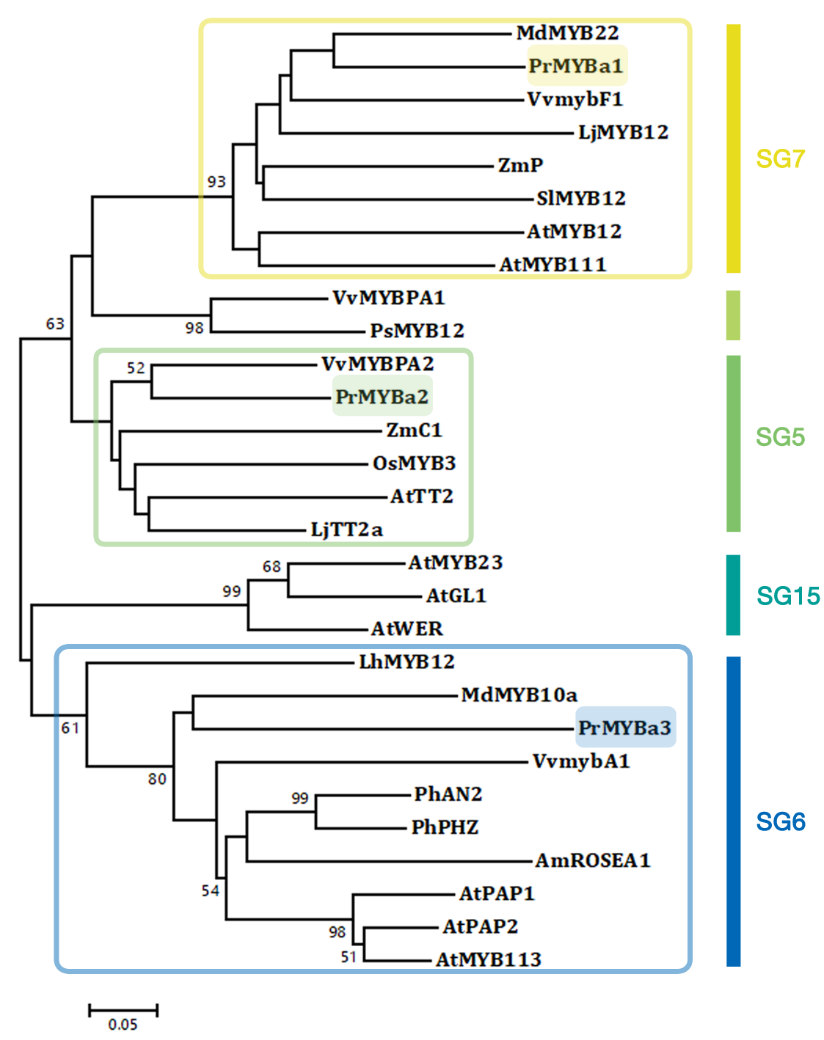
 Phylogenetic analysis of MYB proteins involved in flavonoid biosynthesis. The phylogenetic tree was constructed using the neighbor-joining method, all sequences were retrieved from the NCBI database (https://www.ncbi.nlm.nih.gov/https://www.ncbi.nlm.nih.gov/), the GenBank accession numbers of the MYB proteins are as follows: AtTT2 (NP_198405); LjTT2a (BAG12893.1); VvMYBPA2 (NP_001267953); ZmC1 (AAK09327.1); OsMYB3 (BAA23339.1); AtMYB12 (ABB03913); VvmybF (ACT88298.1); MdMYB22 (AAZ20438.1); LjMYB12 (BAF74782.1); AtMYB111 (NP_199744.1); ZmP (P27898); SlMYB12 (NP_001234401.1); PhAN2 (AAF66727.1); PhPHZ (ADW94951.1); VvmybA1 (BAD18977); AtPAP1 (NP_176057); AtPAP2 (NP_176813.1); LhMYB12 (BAJ05398.1); AtMYB113 (NP_176811.1); MdMYB10a (ABB84754.1); AmROSEA1 (ABB83826); AtMYB23 (NP_198849.1); AtGL1 (NP_189430.1); AtWER (NP_001331610.1); VvMYBPA1 (CAJ90831.1). SG: Sub-group.

Fig. S21.

 The control of McrBC digestion and BSP. (A) The digestion of methylated control plasmid DNA. M, maker. 1, control DNA. 2, digested control DNA. (B) The estimation of BS conversion efficiency. M, maker. N, products amplified using normal primers. T, products amplified using fully transformed primers. D, products amplified using degenerated primers.
