# Supplementary material for: The combination of DNA methylation and positive regulation of anthocyanin biosynthesis by MYB and bHLH transcription factors contributes to the petal blotch formation in Xibei tree peony
Source: Hortic Res. 2023 May 19;10(7):uhad100. doi: 10.1093/hr/uhad100 (PMC10327543; doi:10.1093/hr/uhad100)
Supplement: Web_Material_uhad100 [file web_material_uhad100.zip › Supplementary-TableS1-S2,S4-S6.docx]

**Table S1.** **Putative flavonoids identification in petals of *P. rockii* ‘Shu Sheng Peng Mo’ from the HPLC-MS biochemical analysis**

Petals were separated into purple and white areas for analysis.

Both positive and negative mode ESI MS were employed to identify flavone glycosides and flavonol glycosides, positive mode for anthocyanins.

Identification was based on retention time (tR), maximum absorbance wavelength (λmax) observed in the UV-Vis spectra of the compound that eluted at the tR indicated, molecular mass comparisons with standards and values of previously reported flavonoids for *P. suffruticosa*.

Pn, peonidin; Cy, cyanidin; Ch, chrysoeriol; Is, isorhamnetin; Ap, apigenin; Lu, luteolin; Km, Kaempferol; Qu, quercetin.

| Peaks No. | Putative flavonoid | tR (min) | λ max (nm) | EST-PI MS^2^ (*m/z*) | EST-NI MS^2^ (*m/z*) |
| --- | --- | --- | --- | --- | --- |
| a1 | Cy 3,5-di-*O*-hexoside | 10.1 | 280,516 | 611 [M] ^+^,449[M -162] ^+^,287[Y_0_] ^+^ | - |
| a2 | Cy 3,5-di-*O*- glucoside | 11.6 | 274,514 | 611[M] ^+^,449[M -162] ^+^,287[Y_0_] ^+^ | - |
| a3 | Pn 3,5-di-*O*- glucoside | 14.2 | 278,515 | 625[M] ^+^,465[M +2H-162] ^+^,303[Y_0_+2H] ^+^ | - |
| a4 | Cy 3-di-*O*- glucoside | 16.0 | 279,516 | 449[M] ^+^,287[Y_0_] ^+^ | - |
| a5 | Pn 3-di-*O*- glucoside | 19.4 | 278,518 | 463[M] ^+^,301[Y_0_] ^+^ | - |
| f1 | Qu 3,7-di-*O*-glucoside | 14.6 | 255,353 | 627 [M] ^+^,465[M -162] ^+^,303[Y_0_] ^+^, | 625[M-H]^-^,463[M-H-162] ^-^,301[Y_0_] ^-^ |
| f2 | Km 3,7-di-*O*-hexoside | 17.0 | 264, 348 | 611[M] ^+^,449[M -162] ^+^,287[Y_0_] ^+^ | 609[M-H]^-^,447[M-H-162] ^-^,285[Y_0_] ^-^ |
| f3 | Is 3,7-di-*O*-glucoside | 17.7 | 254,354 | 641[M] ^+^,479[M -162] ^+^,317[Y_0_] ^+^ | 639[M-H]^-^,447[M-H-162] ^-^,315[Y_0_] ^-^ |
| f4 | Qu 7-*O*-glucoside | 25.0 | 254,371 | 465[M] ^+^,303[Y_0_] ^+^ | 463[M-H]^-^,301[M-H-162] ^-^ |
| f5 | Lu 7-*O*-glucoside | 25.8 | 258,350 | 449[M] ^+^,287[Y_0_] ^+^ | 447[M-H]^-^,285[Y_0_] ^-^ |
| f6 | Km 7- *O*-glucoside | 28.6 | 267,338 | 449[M] ^+^,287[Y_0_] ^+^ | 447[M-H]^-^,284[Y_0_-H] ^-^ |
| f7 | Ap 7-*O*-neohesperidoside | 29.3 | 265,338 | 579[M] ^+^,271[Y_0_] ^+^ | 577[M-H]^-^,269[Y_0_] ^-^ |
| f8 | Ap 7-*O*- glucoside | 29.9 | 266,337 | 433[M] ^+^,271[Y_0_] ^+^ | 431[M-H]^-^,269[Y_0_] ^-^ |
| f9 | Ch 7-*O*-neohesperidoside | 30.2 | 253,352 | 609[M] ^+^,301[Y_0_] ^+^ | 607[M-H]^-^,299[Y_0_] ^-^ |
| f10 | Is 7-*O*-glucoside | 30.5 | 254,357 | 479[M] ^+^,317[Y_0_] ^+^ | 477[M-H]^-^,315[Y_0_] ^-^ |
| f11 | Ch 7-*O*-glucoside | 30.8 | 260,349 | 463[M] ^+^,301[Y_0_] ^+^ | 461[M-H]^-^,299[Y_0_] ^-^ |

**Table S2.** **Relative content of individual flavonoids (DW mg/g) in petals**

| Peaks No. | S1 | S2-P | S2-W | S3-P | S3-W | S4-P | S4-W | S5-P | S5-W |
| --- | --- | --- | --- | --- | --- | --- | --- | --- | --- |
| a1 | 0 | 0.009±0.00 | 0 | 0.064±0.03 | 0 | 0.054±0.03 | 0 | 0.064±0.00 | 0 |
| a2 | 0 | 0.565±0.03 | 0 | 0.636±0.05 | 0 | 0.606±0.06 | 0 | 0.616±0.02 | 0 |
| a3 | 0 | 0.412±0.02 | 0 | 0.466±0.04 | 0 | 0.442±0.05 | 0 | 0.423±0.02 | 0 |
| a4 | 0 | 1.55±0.04 | 0 | 2.57±0.6 | 0 | 2.23±0.6 | 0 | 2.52±0.1 | 0 |
| a5 | 0 | 0.251±0.00 | 0 | 0.362±0.07 | 0 | 0.311±0.07 | 0 | 0.321±0.01 | 0 |
| f1 | 0.013±0.00 | 0.445±0.01 | 0.036±0.00 | 0.569±0.03 | 0.422±0.01 | 0.615±0.02 | 0.589±0.01 | 0.627±0.01 | 0.610±0.02 |
| f2 | 0 | 0.153±0.01 | 0.020±0.00 | 0.207±0.01 | 0.202±0.01 | 0.242±0.01 | 0.356±0.01 | 0.262±0.01 | 0.366±0.01 |
| f3 | 0.151±0.00 | 0.828±0.03 | 0.200±0.01 | 1.02±0.05 | 1.07±0.03 | 1.05±0.04 | 1.27±0.02 | 1.03±0.02 | 1.35±0.05 |
| f4 | 0 | 0.165±0.01 | 0.014±0.00 | 0.213±0.01 | 0.111±0.00 | 0.219±0.01 | 0.133±0.00 | 0.221±0.00 | 0.130±0.01 |
| f5 | 0.091±0.00 | 1.89±0.07 | 2.39±0.13 | 2.34±0.13 | 6.10±0.17 | 2.27±0.09 | 5.46±0.13 | 2.07±0.04 | 5.77±0.17 |
| f6 | 0 | 0 | 0.023±0.00 | 0.011±0.00 | 0.099±0.00 | 0.010±0.00 | 0.099±0.00 | 0.006±0.00 | 0.107±0.00 |
| f7 | 0.001±0.00 | 0.328±0.02 | 0.824±0.04 | 0.603±0.05 | 2.48±0.08 | 0.585±0.04 | 2.47±0.05 | 0.503±0.02 | 2.65±0.09 |
| f8 | 0.012±0.00 | 0.242±0.01 | 0.340±0.01 | 0.425±0.03 | 1.71±0.06 | 0.455±0.02 | 2.09±0.04 | 0.411±0.01 | 2.14±0.08 |
| f9 | 0.015±0.00 | 0.294±0.01 | 0.546±0.03 | 0.292±0.02 | 0.892±0.02 | 0.273±0.01 | 0.694±0.02 | 0.243±0.01 | 0.804±0.04 |
| f10 | 0 | 0.17±0.01 | 0 | 0.231±0.01 | 0.123±0.01 | 0.230±0.01 | 0.153±0.00 | 0.223±0.00 | 0.144±0.01 |
| f11 | 0.01±0.00 | 0.100±0.01 | 0.195±0.01 | 0.103±0.01 | 0.352±0.01 | 0.099±0.01 | 0.305±0.01 | 0.088±0.01 | 0.333±0.01 |

Petals are separated into purple and white areas for analysis, labeled as: P or W.

**Table S4.** **Sequence information of PCR primers used in this study**

| **Application** | **Name** | | **Sequence (5′-3′)** |
| --- | --- | --- | --- |
| **qRT-PCR** | qPrCHS | F | CGGCAATCATGGCAATTGGAACAG |
|  |  | R | TGTCTTGTCTTGCGTCCAGTGATG |
|  | qPrCHI | F | CTGGGCCATGGTCTTACCGA |
|  |  | R | GCCAATCGATCCCTCACTGC |
|  | qPrF3H | F | TCCTCAACCGGACCTTACTCTCG |
|  |  | R | GGCTGTAGTTCGAGTTCACCACTG |
|  | qPrF3’H | F | ATTTAGGCGACTTTGTTCCAG |
|  |  | R | CTCTATTCTTCTTATGCTCCTCC |
|  | qPrFNS | F | AGGCAACCGAACTCTCAACCATT |
|  |  | R | CTCCGAAAATCTGTGTCACCTCCCT |
|  | qPrF3H-2 | F | ATGCTGGTTAATCAGTGGCCTCAC |
|  |  | R | TTGTGACGACCAACACCAAGAGC |
|  | qPrDFR | F | ACAGTGCGGGATCCAGAGAA |
|  |  | R | GCCTTGGCACATGCTCTCAT |
|  | qPrANS | F | ACTGGGAAGATTGCTGGGTAT |
|  |  | R | AGCCGATTGACAAGATTGATAAG |
|  | qPrOMT | F | AAGCACCCTACGATCAAGG |
|  |  | R | GGGAGAATACATTCGCACA |
|  | qPrMYBa1 | F | CAACCGCCTCACACCAAAAGTCAAG |
|  |  | R | GGTCATCATTACCAGTCACCGTCAA |
|  | qPrMYBa2 | F | GGCGAACAGACAATGAAATCAAGAA |
|  |  | R | AGATTCTGCTGCCACATTTCTGACC |
|  | qPrMYBa3 | F | GACCAAGAGAATGTCAAATCCACCC |
|  |  | R | ATTATCCAACAACCTTTCCCACCAC |
|  | qPrbHLH1 | F | GGTTGGTTGAACACCCATCTATCTGC |
|  |  | R | AAAGAAGGAACCATAGACCTAAGAATCAT |
|  | qPrbHLH2 | F | GCACTATCAAAGTGTACTGTCATCGCT |
|  |  | R | ACTTCAAACAGTGCCTTTTTCAATAAT |

| **Application** | **Name** | | **Sequence (5′-3′)** |
| --- | --- | --- | --- |
| **qRT-PCR** | qPstublin | F | GCACCAAAGAAGTGGACGAACAAAT |
|  |  | R | AGTAAACTGTTCACTCACACGCCTG |
|  | qNtCHS | F | TTGTTCGAGCTTGTCTCTGC |
|  |  | R | AGCCCAGGAACATCTTTGAG |
|  | qNtCHI | F | GTCAGGCCATTGAAAAGCTC |
|  |  | R | CTAATCGTCAATGCCCCAAC |
|  | qNtF3H | F | CAAGGCATGTGTGGATATGG |
|  |  | R | TGTGTAGTTTCAGTCCAAGG |
|  | qNtF3’H | F | TGTGCACCACGAATGCACTT |
|  |  | R | TCAAGAACGCGTCGAAACG |
|  | qNtDFR | F | AACCAACAGTCAGGGGAATG |
|  |  | R | TTGGACATCGACAGTTCCAG |
|  | qNtANS | F | TGGCGTTGAAGCTCATACTG |
|  |  | R | GGAATTAGGCACACACTTTGC |
|  | qNtFLS | F | CTTGAAGGGAAAAGGGGTTGG |
|  |  | R | CGCAACTTCTCGCAGCCTCT |
|  | qNtEF1*α* | F | TGGTTGTGACTTTTGGTCCCA |
|  |  | R | ACAAACCCACGCTTGAGATCC |
| **Y1H** | pLacZi-PF3H | F | **TTGATATTGGATCGGAATTC**TGAGAGACAAAACGTGTTAAATATGCG |
|  |  | R | **GAGGTCGACAGATCCCGGG**TGTTCCTTTATTTTCTTTCTCTACACA |
|  | pLacZi-PDFR | F | **TTGATATTGGATCGGAATTC**TGGTCAGGCAGAAAAGAGGCAGCGG |
|  |  | R | **GAGGTCGACAGATCCCGGG**TTGCTTTTGTTTTTTAACCACGATA |
|  | pLacZi-PANS | F | **TTGATATTGGATCGGAATTC**GATGGCATCTAACACGATATTGGTA |
|  |  | R | **GAGGTCGACAGATCCCGGG**TTTTGCAGCAACGTTTACTCTCTGTT |
|  | pJG4-5-U-22164 | F | **GATTATGCCTCTCCCGAATTC**ATGGGGAGGGCACCCTGTTGTGAGA |
|  |  | R | **AGAAGTCCAAAGCTTCTCGAG**TCAAGAAAGAAGCCAAGCAACCAT |
|  | pJG4-5-U-210213 | F | **GATTATGCCTCTCCCGAATTC**ATGGGTAGAAGTCCTTGTTGTTCTAAAG |
|  |  | R | **AGAAGTCCAAAGCTTCTCGAG**TCAATTCGGATAAACAACATTGAGATCA |
|  | pJG4-5-U-216927 | F | **GATTATGCCTCTCCCGAATTC**ATGGAGAGAGTATTAGGGGTGAAAA |
|  |  | R | **AGAAGTCCAAAGCTTCTCGAG**CTACATGTCCTGCCCCCATACGTTT |
|  | pJG4-5-U-184394 | F | **GATTATGCCTCTCCCGAATTC**ATGGGTAGGAATCCATGTTGTGACAAGA |
|  |  | R | **AGAAGTCCAAAGCTTCTCGAG**TCACTGATCACTATGGCCAATTTGCAAG |
| **Application** | **Name** |  | **Sequence (5′-3′)** |
| **Y1H** | pJG4-5-U-126241 | F | **GATTATGCCTCTCCCGAATTC**ATGGGGAGAAGCCCTTGTTGTTCAA |
|  |  | R | **AGAAGTCCAAAGCTTCTCGAG** TTAAACTCCTAGCCAGTCTTCTTCAGGA |
|  | pJG4-5-U-150752 | F | **GATTATGCCTCTCCCGAATTC**ATGAGAAACCCTACATCTGGGTCTGGTA |
|  |  | R | **AGAAGTCCAAAGCTTCTCGAG**TCAGAAATGCTTTTCAACATGCTCATCG |
|  | pJG4-5-U-210991 | F | **GATTATGCCTCTCCCGAATTC**ATGGGAAGGTCTCCTTGTTGTGAGAAAG |
|  |  | R | **AGAAGTCCAAAGCTTCTCGAG** TTACTTCATCTCCAATCTTCTGTAATCCAA |
|  | pJG4-5-U-186385 | F | **GATTATGCCTCTCCCGAATTC**ATGGGCAGATCTCCTTGTTGCGAGAA |
|  |  | R | **AGAAGTCCAAAGCTTCTCGAG**TTAGGGTCTGCAATATCTAAAAAGAG |
|  | pJG4-5-U-22762 | F | **GATTATGCCTCTCCCGAATTC** ATGGGACGCTCTCCTTGTTGTGAGAA |
|  |  | R | **AGAAGTCCAAAGCTTCTCGAG**TTAATTTGTCAAATCAAAAGAACCATTTTTT |
|  | pJG4-5-U-45879 | F | **GATTATGCCTCTCCCGAATTC**ATGAGAAAACCTTGCTGTGATAAAC |
|  |  | R | **AGAAGTCCAAAGCTTCTCGAG**CTATCTAAAAAGAATAAGAGTCG |
|  | pJG4-5-U-80390 | F | **GATTATGCCTCTCCCGAATTC**ATGAGGAAACCTTGCTGTGACAAGCA |
|  |  | R | **AGAAGTCCAAAGCTTCTCGAG**TCATCTGAAAAGAAGAAGCGTACTCG |
|  | pJG4-5-PsMYB12 | F | **GATTATGCCTCTCCCGAATTC**ATGGGAAGGGCTCCTTGTTGTTCAAA |
|  |  | R | **AGAAGTCCAAAGCTTCTCGAG**CTACTGCTGCTGCTGCTGCTTATTCTCT |
|  | pJG4-5-PrbHLH1 | F | **GATTATGCCTCTCCCGAATTC**ATGGTTACTGGGATGCAAAACCAAGAG |
|  |  | R | **AGAAGTCCAAAGCTTCTCGAG**TTAGTTCACACCAGTGATTTTCAAAAGTG |
|  | pJG4-5-PrbHLH2 | F | **GATTATGCCTCTCCCGAATTC**ATGGCAACTGGGCTCCAAATTCAGGA |
|  |  | R | **AGAAGTCCAAAGCTTCTCGAG**CTAACACTTGTGAATGACTCTCCGAA |
|  | pJG4-5-PrbHLH3 | F | **GATTATGCCTCTCCCGAATTC**ATGGCTGCGCCGCCCGTAAACAGCCGGTT |
|  |  | R | **AGAAGTCCAAAGCTTCTCGAG**TCAAGGAATTATTTGGTGTATTGCTCGCTT |
|  | LacZi-PAtLODX | F | **TTGATATTGGATCGGAATTC**TTAGTCCCATAGTTGATGATTACTTAGC |
|  |  | R | **GAGGTCGACAGATCCCGGG**CTTCTTTAGTCTTCTGTTTAAA |
|  | pJG4-5-AtPAP1 | F | **GATTATGCCTCTCCCGAATTC**ATGGAGGGTTCGTCCAAAGGGC |
|  |  | R | **AGAAGTCCAAAGCTTCTCGAG**CTAATCAAATTTCACAGTCTCT |
| **EMAS** | pGEX6p-PrMYBa1 | F | **GCCCCTGGGATCCCCGGAATTC**ATGGGGAGGGCACCCTGTTGTGA |
|  |  | R | **AGTCACGATGCGGCCGCTCGAG**TCAAGAAAGAAGCCAAGCAACCAT |
|  | pGEX6p-PrMYBa3 | F | **GCCCCTGGGATCCCCGGAATTC**ATGGAGAGAGTATTAGGGGTGAAAA |
|  |  | R | **AGTCACGATGCGGCCGCTCGAG**CTACATGTCCTGCCCCCATACGTTT |
| **LUC assays** | pGreenII-PF3H | F | **GGTCGACGGTATCGATAAGCTT**TGAGAGACAAAACGTGTTAAATATGCG |
|  |  | R | **CCGCTCTAGAACTAGTGGATCC**TGTTCCTTTATTTTCTTTCTCTACACA |
| **Application** | **Name** | | **Sequence (5′-3′)** |
| **LUC assays** | pGreenII-PF3H | F | **GGTCGACGGTATCGATAAGCTT**TGAGAGACAAAACGTGTTAAATATGCG |
|  |  | R | **CCGCTCTAGAACTAGTGGATCC**TGTTCCTTTATTTTCTTTCTCTACACA |
|  | pGreenII-PDFR | F | **GGTCGACGGTATCGATAAGCTT**TGGTCAGGCAGAAAAGAGGCAGCGG |
|  |  | R | **CCGCTCTAGAACTAGTGGATCC**TTGCTTTTGTTTTTTAACCACGATA |
|  | pGreenII-PANS | F | **GGTCGACGGTATCGATAAGCTT**GATGGCATCTAACACGATATTGGTA |
|  |  | R | **CCGCTCTAGAACTAGTGGATCC**TTTTGCAGCAACGTTTACTCTCTGTT |
|  | pSN1301-PrMYBa1 | F | **ACGGGGGACTCTAGAGGATCC**ATGGGGAGGGCACCCTGTTGTGAGA |
|  |  | R | **CGATCGGGGAAATTCGAGCTC**TCAAGAAAGAAGCCAAGCAACCAT |
|  | pSN1301-PrMYBa2 | F | **ACGGGGGACTCTAGAGGATCC**ATGGGTAGAAGTCCTTGTTGTTCTAAAG |
|  |  | R | **CGATCGGGGAAATTCGAGCTC**TCAATTCGGATAAACAACATTGAGATCA |
|  | pSN1301-PrMYBa3 | F | **ACGGGGGACTCTAGAGGATCC**ATGGAGAGAGTATTAGGGGTGAAAA |
|  |  | R | **CGATCGGGGAAATTCGAGCTC**CTACATGTCCTGCCCCCATACGTTT |
|  | pSN1301-PrbHLH1 | F | **ACGGGGGACTCTAGAGGATCC**ATGGTTACTGGGATGCAAAAC |
|  |  | R | **CGATCGGGGAAATTCGAGCTC**TTAGTTCACACCAGTGATTTTC |
|  | pSN1301-PrbHLH2 | F | **ACGGGGGACTCTAGAGGATCC**ATGGCAACTGGGCTCCAAATTCAG |
|  |  | R | **CGATCGGGGAAATTCGAGCTC**CTAACACTTGTGAATGACTCTCCG |
|  | pSN1301-PrbHLH3 | F | **ACGGGGGACTCTAGAGGATCC**ATGGCTGCGCCGCCCGTAAACAGCCGGTT |
|  |  | R | **CGATCGGGGAAATTCGAGCTC**TCAAGGAATTATTTGGTGTATTGCTCGCTT |
| **Y2H** | pGBK-PrMYBa1 | F | **ATGGCCATGGAGGCCGAATTC**ATGGGGAGGGCACCCTGTTGTGAGA |
|  |  | R | **CCGCTGCAGGTCGACGGATCC**TCAAGAAAGAAGCCAAGCAACCAT |
|  | pGBK-PrMYBa2 | F | **ATGGCCATGGAGGCCGAATTC**ATGGGTAGAAGTCCTTGTTGTTCTAAAG |
|  |  | R | **CCGCTGCAGGTCGACGGATCC**TCAATTCGGATAAACAACATTGAGATCA |
|  | pGBK-PrMYBa3 | F | **ATGGCCATGGAGGCCGAATTC**ATGGAGAGAGTATTAGGGGTGAAAA |
|  |  | R | **CCGCTGCAGGTCGACGGATCC**CTACATGTCCTGCCCCCATACGTTT |
|  | pGBK-PrbHLH1 | F | **ATGGCCATGGAGGCCGAATTC**ATGGTTACTGGGATGCAAAAC |
|  |  | R | **CCGCTGCAGGTCGACGGATCC**TTAGTTCACACCAGTGATTTTC |
|  | pGBK-PrbHLH2 | F | **ATGGCCATGGAGGCCGAATTC**ATGGCAACTGGGCTCCAAATTCAG |
|  |  | R | **CCGCTGCAGGTCGACGGATCC**CTAACACTTGTGAATGACTCTCCG |
|  | pGBK-PrbHLH3 | F | **ATGGCCATGGAGGCCGAATTC**ATGGCTGCGCCGCCCGTAAACAGCCGGTT |
|  |  | R | **CCGCTGCAGGTCGACGGATCC**TCAAGGAATTATTTGGTGTATTGCTCGCTT |
|  | pGAD-PrMYBa1 | F | **GCCATGGAGGCCAGTGAATTC**ATGGGGAGGGCACCCTGTTGTGAGA |
|  |  | R | **CAGCTCGAGCTCGATGGATCC**TCAAGAAAGAAGCCAAGCAACCAT |
| **Application** | **Name** | | **Sequence (5′-3′)** |
| **Y2H** | pGAD-PrMYBa2 | F | **GCCATGGAGGCCAGTGAATTC**ATGGGTAGAAGTCCTTGTTGTTCTAAAG |
|  |  | R | **CAGCTCGAGCTCGATGGATCC**TCAATTCGGATAAACAACATTGAGATCA |
|  | pGAD-PrMYBa3 | F | **GCCATGGAGGCCAGTGAATTC**ATGGAGAGAGTATTAGGGGTGAAAA |
|  |  | R | **CAGCTCGAGCTCGATGGATCC**CTACATGTCCTGCCCCCATACGTTT |
|  | pGAD-PrbHLH1 | F | **GCCATGGAGGCCAGTGAATTC**ATGGTTACTGGGATGCAAAAC |
|  |  | R | **CAGCTCGAGCTCGATGGATCC**TTAGTTCACACCAGTGATTTTC |
|  | pGAD-PrbHLH2 | F | **GCCATGGAGGCCAGTGAATTC**ATGGCAACTGGGCTCCAAATTCAG |
|  |  | R | **CAGCTCGAGCTCGATGGATCC**CTAACACTTGTGAATGACTCTCCG |
|  | pGAD-PrbHLH3 | F | **GCCATGGAGGCCAGTGAATTC**ATGGCTGCGCCGCCCGTAAACAGCCGGTT |
|  |  | R | **CAGCTCGAGCTCGATGGATCC**TCAAGGAATTATTTGGTGTATTGCTCGCTT |
| **BIFC assay** | YFP-PrMYBa1 | F | **TGGCGCGCCACTAGTGGATCC**ATGGGGAGGGCACCCTGTTGTGAGA |
|  |  | R | **CCCGGGAGCGGTACCCTCGAG**AGAAAGAAGCCAAGCAACCAT |
|  | YFP-PrMYBa2 | F | **TGGCGCGCCACTAGTGGATCC**ATGGGTAGAAGTCCTTGTTGTTCTAAAG |
|  |  | R | **CCCGGGAGCGGTACCCTCGAG**ATTCGGATAAACAACATTGAGATCA |
|  | YFP-PrMYBa3 | F | **TGGCGCGCCACTAGTGGATCC**ATGGAGAGAGTATTAGGGGTGAAAA |
|  |  | R | **CCCGGGAGCGGTACCCTCGAG**CATGTCCTGCCCCCATACGTTT |
|  | YFP- PrbHLH1 | F | **TGGCGCGCCACTAGTGGATCC**ATGGTTACTGGGATGCAAAAC |
|  |  | R | **CCCGGGAGCGGTACCCTCGAG**GTTCACACCAGTGATTTTC |
|  | YFP- PrbHLH2 | F | **TGGCGCGCCACTAGTGGATCC**ATGGCAACTGGGCTCCAAATTCAG |
|  |  | R | **CCCGGGAGCGGTACCCTCGAG**ACACTTGTGAATGACTCTCCG |
|  | YFP- PrbHLH2 | F | **TGGCGCGCCACTAGTGGATCC**ATGGCAACTGGGCTCCAAATTCAG |
|  |  | R | **CCCGGGAGCGGTACCCTCGAG**ACACTTGTGAATGACTCTCCG |
| **Pull-down assa** | pET28a-PrMYBa1 | F | **CAGCAAATGGGTCGCGGATCC**ATGGGGAGGGCACCCTGTTGTGA |
|  |  | R | **GTGGTGGTGGTGGTGCTCGAG**AGAAAGAAGCCAAGCAACCAT |
|  | pGEX6p-PrMYBa2 | F | **GCCCCTGGGATCCCCGGAATTC**ATGGGTAGAAGTCCTTGTTGTTCTAAAG |
|  |  | R | **AGTCACGATGCGGCCGCTCGAG**TCAATTCGGATAAACAACATTGAGATCA |
| **Co-IP assays** | pGFP-PrMYBa1 | F | **GACTCTAGTCTAGAAAGCTT**ATGGGGAGGGCACCCTGTTGTG |
|  |  | R | **CCCTTGCTCACCATGGTACC**AGAAAGAAGCCAAGCAACCAT |
|  | pFlag- PrMYBa2 | F | **GAGAACACGGGGGACTCTAGA**ATGGGTAGAAGTCCTTGTTGTTC |
|  |  | R | **GTCTTTGTAGTCCATGGTACC**ATTCGGATAAACAACATTGAGA |
|  | pFlag- PrMYBa3 | F | **GAGAACACGGGGGACTCTAGA**ATGGAGAGAGTATTAGGGGTGAA |
|  |  | R | **GTCTTTGTAGTCCATGGTACC**CATGTCCTGCCCCCATACGTTT |
| **Application** | **Name** | | **Sequence (5′-3′)** |
| **Co-IP assays** | pHA- PrbHLH1 | F | **ACGGGGGACTCTAGAGGATCC**ATGGTTACTGGGATGCAAAAC |
|  |  | R | **ATCGTATGGGTACATGGTACC**GTTCACACCAGTGATTTTC |
|  | pHA- PrbHLH2 | F | **ACGGGGGACTCTAGAGGATCC**ATGGCAACTGGGCTCCAAATTCAG |
|  |  | R | **ATCGTATGGGTACATGGTACC**ACACTTGTGAATGACTCTCCG |
| **Plant transformation** | pSN1301-PrMYBa1 | F | **ACGGGGGACTCTAGAGGATCC**ATGGGGAGGGCACCCTGTTGTGAGA |
|  |  | R | **CGATCGGGGAAATTCGAGCTC**TCAAGAAAGAAGCCAAGCAACCAT |
|  | pSN1301-PrMYBa3 | F | **ACGGGGGACTCTAGAGGATCC**ATGGAGAGAGTATTAGGGGTGAAAA |
|  |  | R | **CGATCGGGGAAATTCGAGCTC**CTACATGTCCTGCCCCCATACGTTT |
|  | p1302-MYBa1 | F | **GGACTCTTGACC**ATGGGGAGGGCACCCTGTTGT |
|  |  | R | **ACGAATTCGAGCTCGGTACC**CCGATCTAGTAACATAGATGACACC |
|  | p1302-MYBa2 | F | **GGACTCTTGACC**ATGGGTAGAAGTCCTTGTTGTTC |
|  |  | R | **GTTCTTCTCCTTTACTAGT**TTACTTATCGTCATC |
|  | p1302-MYBa3 | F | **GGACTCTTGACC**ATGGAGAGAGTATTAGGGGTGAA |
|  |  | R | **AGTTCTTCTCCTTTACTAGT**TTACTTATCGTCATC |
|  | p1302-bHLH-box | F | **CGACGGCCAGTGCCAAGC**TTGGTCCCCAGATTAGCCTTTTCAATT |
|  |  | R | **ATGACCATGATTACGAATTC**CCGATCTAGTAACATAGATGACACC |
| **Genome Walking PCR** | ProF3HR1 | | AAGTATGTCACTATTTCACGCCAATCTCGCAC |
|  | ProF3HR2 | | AGGGAGATGATTGGGATTTCGTTGCTGA |
|  | ProF3HR3 | | CACCTGCGAGAGCTGTAAGGGTAGTGGCCGT |
|  | ProDFRR1 | | ACATTCCATTTATTGTTGGCTTTATCACTTCATTC |
|  | ProDFRR2 | | ATATTCTCTGGATCCCGCACTGTGGCTC |
|  | ProDFRR3 | | ATGACAAGCCATGAACCGATGAACCCCG |
|  | ProANSR1 | | GCCATACCCAGCAATCTTCCCAGTCTCT |
|  | ProANSR2 | | CGAAGATGTTGGTGATGCTGGTTTGCTC |
|  | ProANSR3 | | TGGATTGGATCCCACTGATAGCCAAG |
|  | 2ProANSR1 | | GCGTGAAGAAGATGAAGCCAACCACCAGG |
|  | 2ProANSR2 | | TTTATGGGGAATTGAATAGTGAGGAGTTA |
|  | 2ProANSR3 | | ATCCTGTATTAACCGAATCATGTCAAGTCA |
|  | 3ProANSR1 | | GTCAAGTCATGAAAAATAATGGAAGTACAAGATC |
|  | 3ProANSR2 | | CTATTATCATTGTAACTAATTATAAATGCCCATGA |
|  | 3ProANSR3 | | TCAAATTTTTAATCATTTACACTGCATATAGATG |
|  |  | |  |
| **Application** | **Name** | | **Sequence (5′-3′)** |
| **Promoter validation** | YZ-ProF3H | F | TGAGAGACAAAACGTGTTAAATATGCG |
|  |  | R | TCACTATTTCACGCCAATCTCGCACAG |
|  | YZ-ProDFR | F | TGGTCAGGCAGAAAAGAGGCAGCGG |
|  |  | R | GACACACACGGTTTCAGCATCGCACTCG |
|  | YZ-ProANS | F | GATGGCATCTAACACGATATTGGTA |
|  |  | R | TTCCACAGGCTGCTGAAAAAAAACC |

**Table S5.** **Sequence information of primers used in McrBC-PCR analysis**

| **Name** |  | **Sequence (5′-3′)** |
| --- | --- | --- |
| M-PANS-PS1 | F | TACAGTTTAATACTTACACCATA |
|  | R | ATGACAATCTAATGTGGTATTTCTTT |
| M-PANS-PS2 | F | GGGGAGTTCAGTCGGTTGTCTAC |
|  | R | CTATTCAGTCTCACTCAATCCGTT |
| M-PANS-PS3 | F | AAATTTAACGGATTGAGTGAGAC |
|  | R | TTCTGGTTTGACCGAAAGTGTG |
| M-PANS-PS4 | F | TTACACAATAAGGAAGAACGAA |
|  | R | CTATTGATTGTTGTGTGAACTAAGA |
| M-PF3H-PS1 | F | AGAGACAAAACGTGTTAAATATGCG |
|  | R | TACTTCCAGAATAATGAAACTAACTA |
| M-PF3H-PS2 | F | GACTTCCGTTGAAACCTAACCTTG |
|  | R | AGCTGTAAGGGTAGTGGCCGTAG |

**Table S6. Sequence information of primers used in BSP analysis**

| **Name** |  | **Sequence (5′-3′)** |
| --- | --- | --- |
| M-PANS-PS2-1 | F | GGGGAGTTCAGTYGGTTGTYTAY |
|  | R | CTAATGTGGTATTTCTTTRRTTTR |
| M-PANS-PS2-2 | F | CTTCAAACCAAAGAAATAYYAYATTAGAT |
|  | R | CATTTTATCACTCTATTATCATTR |
| M-PANS-PS2-3 | F | GTGAATYATGGGCATTTATAATTAGTT |
|  | R | GATAAGTCRTTTTAARTCTRT |
| M-PANS-PS2-4 | F | GTAACAGAYTTAAAAYGAYTT |
|  | R | CTATTCARTCTCACTCAATCCRTTA |
| M-PANS-PS3-1 | F | YGGATTGAGTGAGAYTGAATAGAT |
|  | R | ATTTATGGGGAATTGAATARTRARRA |
| M-PANS-PS3-2 | F | ACTCCTCACTATTCAATTYYYY |
|  | R | TTCTGGTTTGACCGAAARTRTR |
| M-PF3H-PS1-1 | F | ATAGAGTGAYTCGYTTATAACCCA |
|  | R | GGTTAGRTTTCAACRGAARTCATAC |
| N-fragment 1 | F | ATGTTTGAATGTGATAACCGTCCTT |
|  | R | TCAGAAAGACGATGGGTCGCTAAT |
| T-fragment 1 | F | ATGTTTGAATGTGATAATTGTTTTT |
|  | R | TCAAAAAAACAATAAATCACTAAT |
| D-fragment 1 | F | AYGYYYGAAYGYGAYAAYYGYYYYY |
|  | R | TCRRRRRRRCRRTRRRTCRCTRRT |
| N-fragment 2 | F | TGAGCCGTAGCCACTGTCTGTCC |
|  | R | TAAGCGAGATTGCTACTTAGTCC |
| T-fragment 2 | F | TGAGTTGTAGTTATTGTTTGTTT |
|  | R | TAAACAAAATTACTACTTAATCC |
| D-fragment 2 | F | YGAGYYGYAGYYAYYGYYYGYYY |
|  | R | TRRRCRRRRTTRCTRCTTRRTCC |
